# Anode‐Free Cell Concepts: Critical Analysis and Development of Practical Batteries

**DOI:** 10.1002/smll.202513633

**Published:** 2026-03-09

**Authors:** Svetlana Menkin, Elixabete Ayerbe, Anna B. Gunnarsdóttir, Diego del Olmo, Pedro López‐Aranguren, Amna Rafique, Jokin Rikarte, Susana Chauque, Xiaorui Shi, Olivier Guillon, Robert Dominko, Philipp Schlee, Martin Finsterbusch, Andriy Kvasha, Dina Fattakhova‐Rohlfing

**Affiliations:** ^1^ Yusuf Hamied Department of Chemistry University of Cambridge Cambridge UK; ^2^ The Faraday Institution Didcot UK; ^3^ CIDETEC Basque Research and Technology Alliance (BRTA) Donostia‐San Sebastián Spain; ^4^ Science Institute University of Iceland Reykjavík Iceland; ^5^ Centre for Cooperative Research On Alternative Energies (CIC energiGUNE) Spain; ^6^ Research and Technology Alliance (BRTA) Vitoria‐Gasteiz Spain; ^7^ University of Basque Country (UPV/EHU) Leioa Spain; ^8^ ALISTORE‐European Research Institute Amiens France; ^9^ National Institute of Chemistry Ljubljana Slovenia; ^10^ Forschungszentrum Jülich GmbH Institute of Energy Materials and Devices: Materials Synthesis and Processing (IMD‐2) Jülich Germany

**Keywords:** anode free batteries, degradation mechanisms, electrolyte, Li plating, Na plating, practical batteries, solid state batteries

## Abstract

Anode‐free batteries (AFBs) are one of the most discussed battery concepts due to their potential advantages in terms of energy density, reduced manufacturing costs, and improved sustainability compared to conventional lithium‐ion and lithium metal batteries. However, many fundamental and practical challenges remain to be overcome in order to realize their full potential. This Perspective provides a critical overview of the latest advances in liquid and solid electrolyte AFBs, including an analysis of practical cell performance and an assessment of the advantages and challenges of this cell concept. Since the processes at the negative current collector are central to the electrochemical performance of AFBs, they are discussed in detail, with a focus on metal plating/stripping mechanisms and key degradation processes, as well as strategies for optimizing electrode properties to enable stable cycling. In addition, the associated processes in other cell components (positive electrode, electrolyte, and critical interfaces) are discussed to understand their influence on the overall performance of the cell. Finally, we identify critical gaps in understanding, data accessibility, reporting standards, and metrics that need to be addressed to guide future research and the transition from laboratory scale to practical, high‐performance devices.

## Graphical abstract

Anode‐free batteries (AFBs) are considered one of the most promising battery technologies due to their potential advantages in terms of energy density, manufacturing ease, and sustainability. This Perspective article provides a comprehensive overview of the latest advances in AFBs with liquid and solid electrolytes, a critical analysis of cell performance, and an assessment of the advantages and challenges of this cell concept. The insights gained are used to evaluate concepts for practical AFBs and guide the optimization of their performance.

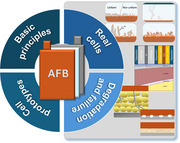

## 1 Introduction

The search for rechargeable batteries with higher energy density has led to a renaissance of interest in lithium (Li) metal anodes. However, potential safety issues due to Li dendrites growth and low Coulombic efficiency during multiple cycling are delaying the practical implementation of lithium metal batteries (LMB). Furthermore, the use of an excessively thick Li metal layer in complete battery cells reduces the practical volumetric energy density, increases material and production costs, raises safety concerns in battery manufacturing, and compromises sustainability, as Li is classified as a critical raw material by the European Commission [1].

“Anode‐free” batteries (AFB), also known as “Li‐free”, “anode‐less”, or “zero excess alkali metal” batteries, emerged as an attempt to overcome the challenges associated with LMBs. In an AFB configuration, the electrochemically refined lithium metal anode is formed in situ by the reduction of Li ions originating from the cathode at the negative current collector during cell charging. Upon discharging, the plated Li metal is stripped out in the form of Li^+^ ions into the electrolyte and intercalated into the cathode active material. This configuration is intended to significantly reduce the amount of Li in the cell (often referred to as excess Li), which should result in higher volumetric energy density. In addition, the use of in situ formed, extra pure Li metal anode can improve the electrochemical performance of the final cell. Another potential advantage of the AFB configuration is that it eliminates the need to handle reactive Li metal anodes during cell assembly, which simplifies supply chain, manufacturing, reduces production costs, and increases safety during production and operation.

**SCHEME 1:**
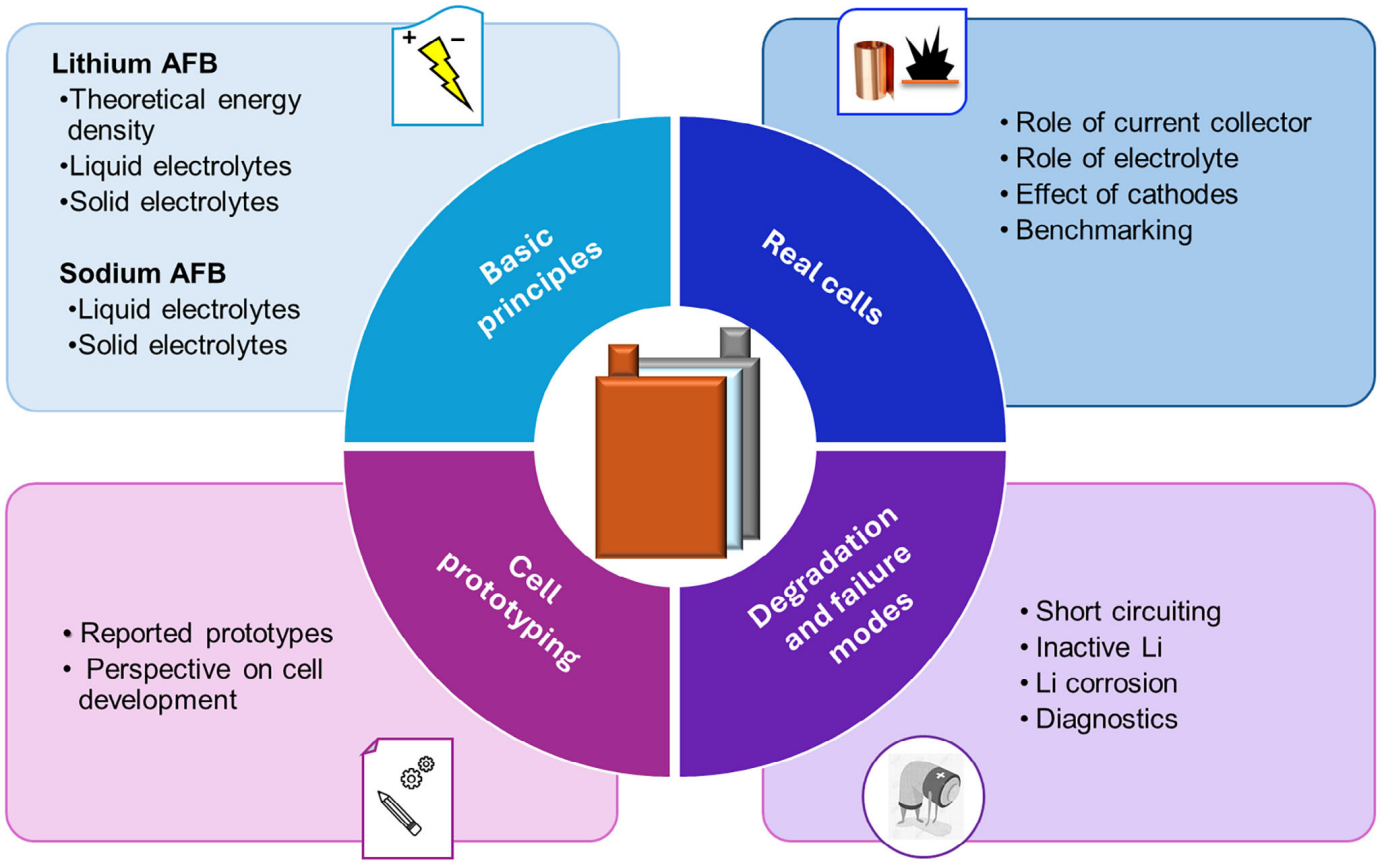
Scope of the Perspective article on anode‐free cell concepts.

Due to their unique advantages, AFBs have become one of the most promising and widely discussed battery concepts in recent years, triggering intensive research in both academia and industry [2, 3, 4, 5, 6]. This is reflected in a growing number of publications and patents dealing with material development, processing strategies, and mechanistic investigations, making it increasingly difficult to gain a clear overview of the field and evaluate promising directions for future research. However, since AFBs are still far from being a mature technology, a deeper understanding is important, and many fundamental and practical challenges still need to be solved in order to realize their full potential. From this perspective, we have set ourselves the goal of providing a comprehensive overview of the latest advances in various areas of anode‐free battery technology, with a critical analysis of practical cell performance and an assessment of the potential advantages and existing challenges of this cell concept. We analyze the processes that can limit the performance of anode‐free cells in a holistic approach that considers the interactions between all battery components and possible degradation processes. An important question here is what theoretical energy density can be expected from AFBs and how it is influenced by the cell configuration. Therefore, as part of our analysis, we present a model that takes into account not only the active cell components, which often overestimate energy density, but also passive components such as current collectors and various electrode modification layers. We then discuss the processes that occur in real cells with liquid and solid electrolytes and analyze their role in cell performance. Since the processes at the anode current collector are central to the electrochemical performance of AFBs, we discuss them in detail, focusing on Li plating/stripping mechanisms, various degradation processes, and strategies for optimizing electrode properties to enable stable plating/stripping without metal dendrites formation. In addition, the associated processes in other cell components (positive electrode, electrolyte, and critical interfaces) are discussed to understand their influence on the overall performance of the cell. Similar developments in sodium anode‐free batteries are also discussed. The insights gained from a comprehensive literature review are then used to evaluate concepts for anode‐free cells in practice and to guide measures for optimizing their performance. The scope of our Perspective paper is outlined in Scheme 1.

## 2 Basic Principles of AFBs and Evaluation of Theoretical Energy Density Values for Selected Cell Designs

**FIGURE 1:**
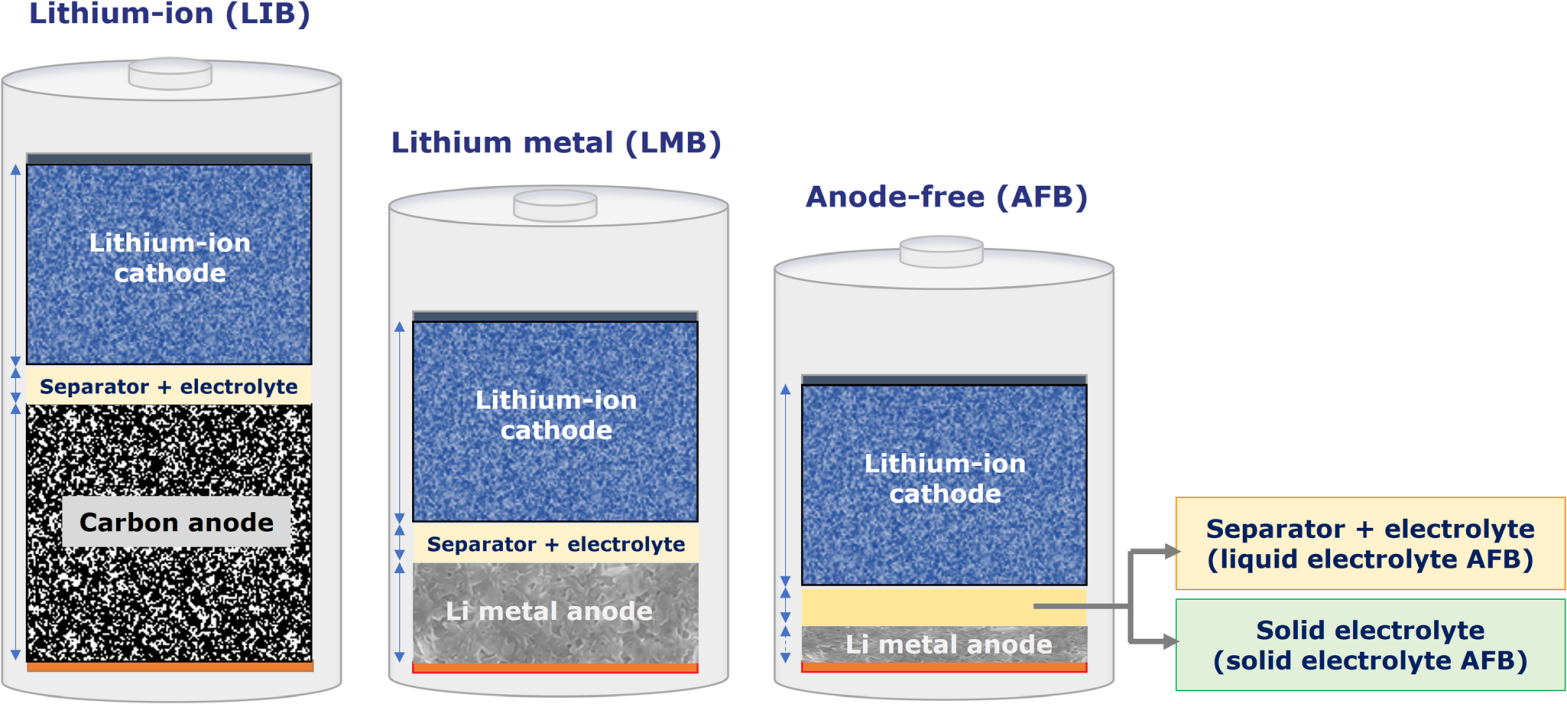
Schematic presentation of metal‐ion, metal and anode‐free batteries (in a fully charged state).

Lithium (Li) AFBs are similar to lithium metal batteries (LMBs), but with an important difference: no Li metal is present during cell assembly; it is only formed during charging by electroplating on a negative current collector. In a general configuration (Figure 1), AFBs consist of a positive current collector (e.g., Al) and a Li‐ion cathode, which serves as the sole source of lithium, as well as a negative current collector (e.g., Cu), onto which the Li metal anode is electrochemically plated during the first charge. Like their lithium metal analogues, AFBs can be generally divided into two categories, namely AFBs with liquid and solid electrolytes. In AFBs with liquid electrolyte, a porous separator soaked with the electrolyte is placed between the positive and negative electrodes. In solid electrolyte AFBs (AFSSBs), the solid electrolyte itself serves as the separator layer, although the design of practical cells can vary considerably.

In this section, we will discuss recent advances in the development of AFBs with liquid electrolytes. An overview of AFBs with solid electrolytes is provided in Section 4.

At first glance, it is reasonable to expect that the transition from conventional LIBs with graphite anodes to an anode‐free configuration will result in a significant improvement in energy density.

When the graphite anode is replaced by a metal foil, an increase in volumetric energy density of up to 63% can be expected due to the different energy densities of the anode materials. The switch to an anode‐free configuration is expected to increase the volumetric energy density by a further 80%–100%, as a much thinner Li layer (a few micrometers vs. ≤50 micrometers) can be electrodeposited during the formation cycles. However, the effects of the cell configuration, such as the cathode load and the parameters of the non‐active cell components, such as the thickness of the current collector, are often overlooked in the available literature, which frequently leads to exaggerated energy density values. To obtain more realistic theoretical energy density values at the cell level and determine the optimal cell configuration, we performed parametric studies of different AFB cell designs using CIDETEC´s proprietary PROTEO tool [7]. The gravimetric and volumetric energy density of the cells was estimated using a physical battery model that solves the standard Doyle‐Fuller‐Newman (DFN) equations and was adapted to account for the influence of the cell configuration [8].

As a basis for the calculations, we selected cell configurations based on components currently used in practical cells with liquid electrolytes. Bare copper foils (Cu, 8.96 g cm^−3^) with a thickness of 4 µm and 8 µm or, alternatively, 8 µm thick Cu foils with a 4 µm thick lithiophilic layer of Ag (10.49 g cm^−3^) or Zn (7.14 g cm^−3^) were selected as negative current collectors for the AFBs (the role of current collectors and lithiophilic layers in AFB performance is discussed in Section 3.2). Graphite anodes (Gr) with a density of 1.6 g cm^−3^, porosity 25%, 93 wt.% active material, a specific capacity of 370 mAh $g_{\mathrm{AM}}^{-1}$, and N/P ratio of 1.1 were selected as negative electrodes for lithium‐ion batteries (LIBs). The thickness of the Li metal anodes (0.534 g cm^−3^) was varied from 25 to 100 µm. For all cell configurations considered, the remaining components were kept constant: (i) cathode: NMC622 with a density of 2.7 g cm^−3^, a porosity of 31%, 95.5 wt.% of active material, and a practical specific capacity of 175 mAh $g_{\mathrm{AM}}^{-1}$, (ii) positive current collector: Al—15 µm (density 2.71 g cm^−3^), and (iii) separator: H2010 (Celgard), porosity 46%, density 0.95 g cm^−3^. We used 1 M LiPF_6_ in EC/DMC as a liquid electrolyte (density 1.24 g cm^−3^) in our calculations, but the use of other carbonate electrolytes should yield comparable results since their densities are similar. Furthermore, no electrolyte excess was assumed in the calculations: the amount of electrolyte was limited to the exact volume required to fill the pores of the separator and the porous electrodes.

For the study, the components were combined in different cell configurations, which are summarized in Table 1, and the influence of each component on the final results was evaluated.

**TABLE 1:** Cell configurations used for calculating the theoretical energy density.

Case number	Configuration	Description
Case 1	Cu/Gr/Sep/NMC622/Al	Li‐ion with anode current collector (standard)
Case 2	Li/Sep/NMC622/Al	Li‐metal without anode current collector
Case 3	Cu/Li/Sep/NMC622/Al	Li‐metal with anode current collector
Cases 4 and 5	Cu/Sep/NMC622/Al	Anode‐free with Cu current collector (4) 4 µm, (5) 8 µm
Cases 6 and 7	Cu(Zn|Ag)/Sep/NMC622/Al	Anode‐free with 8 µm Cu current collector and lithiophilic metal coating (6) 4 µm Zn, (7) 4 µm Ag

**FIGURE 2:**
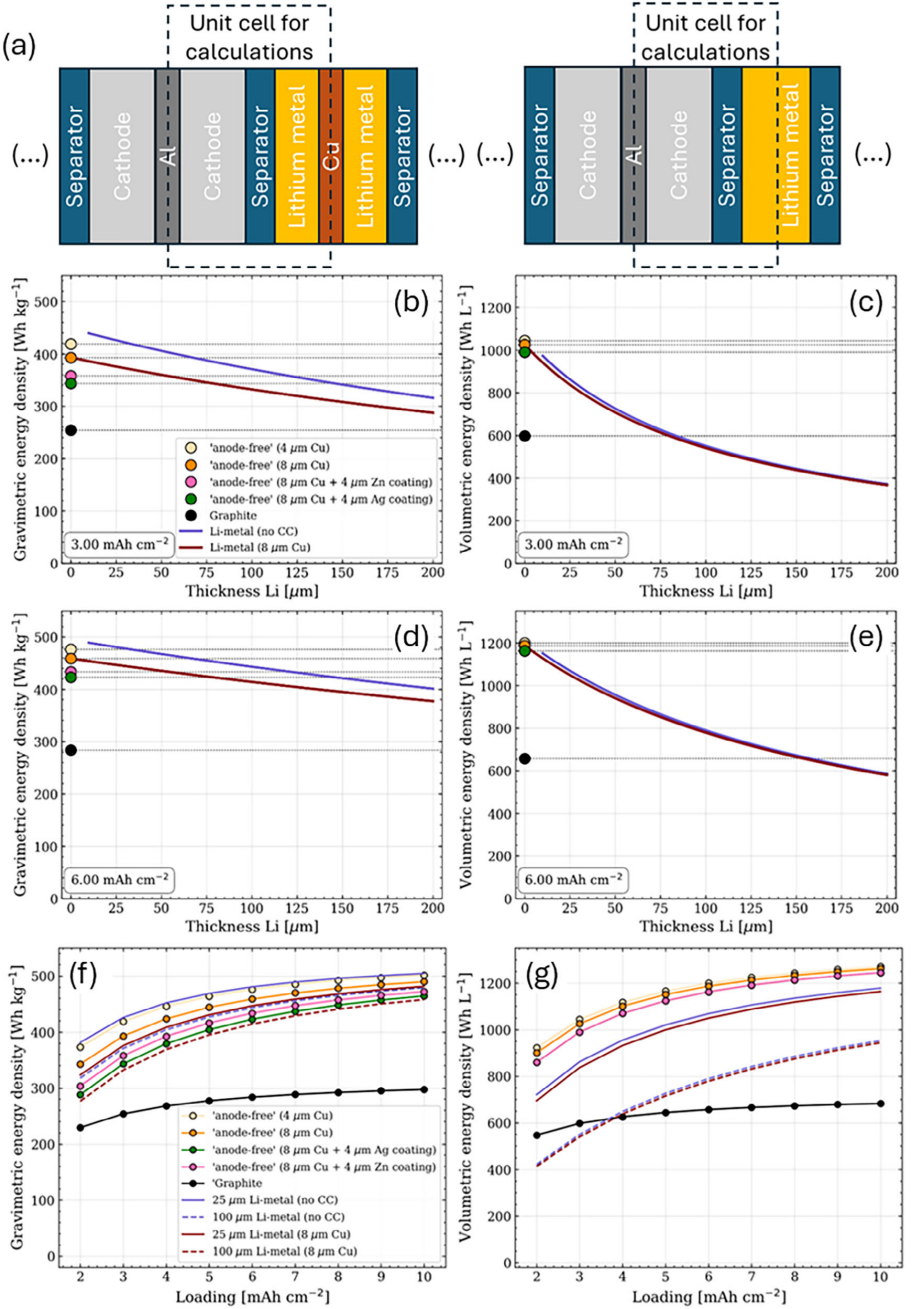
Top: (a) cell design scheme used for the calculations with and without negative current collector. Bottom: gravimetric energy density as function of Li thickness at a positive electrode loading of 3 mAh cm^−2^ (b) and 6 mAh cm^−2^ (d). Volumetric energy density as a function of Li thickness at a positive electrode loading of 3 mAh cm^−2^ (c) and 6 mAh cm^−2^ (e). Gravimetric (f) and volumetric (g) energy density as a function of NMC622‐based cathode loading.

First, we investigated how replacing graphite anodes with Li foil affects the gravimetric and volumetric energy of the cells. The calculations were performed for different Li foil thicknesses and different current collectors to determine how the energy density changes when Li is removed in different cell configurations. The entire process was repeated for both low and high positive electrode loadings (Figure 2).

Figure 2b–e shows that, as expected, the performance of the LIB configuration with graphite anode is worse in terms of both gravimetric and volumetric energy density. After switching to a system with Li foil (i.e., an LMB configuration), a significant improvement in energy density can be observed, especially in cases without a Cu current collector (although this configuration is considered impractical). In both scenarios, the influence of Li metal thickness follows the same trend: a linear relationship for the gravimetric density and a more pronounced effect for the volumetric density. However, when the Li metal was also removed (corresponding to the AFB configuration), our simulations showed virtually no further improvement in energy density. Furthermore, we found that increasing the thickness of the current collectors or adding lithiophilic components such as Ag or Zn (described in more detail in sections 3.2 and 4) further reduced the gravimetric energy density of anode‐free cells.

Compared to the influence of the anode, the loading of the positive electrode has a much greater influence on the energy density, which exceeds 450 Wh kg^−1^ and 1200 Wh L^−1^ at 6 mAh cm^−2^. This is not surprising, as the cathode loading generally determines the capacity and energy density of the entire cell.

Since we recognized the influence of electrode loading on the analyzed performance, we evaluated its impact on the results. Accordingly, a system was investigated in which the loading varied between 2 and 10 mAh cm^−2^, which corresponds to a relatively high loading of the positive electrode. As shown in Figure 2f,g, both the gravimetric and volumetric energy densities improve with increasing positive electrode loading. However, it is important to emphasize that this improvement is less pronounced in the LIB with a Gr anode than in a cell with a Li metal anode. This difference is due to the fact that the anode mass increases with the loading of Gr anodes, while it remains constant for the Li metal anodes and the AFBs. As for the thickness of the Li anode, it is evident that the increase is substantial when the Li metal is thin, with the energy density increasing from approximately 220 to 380 Wh kg^−1^ and from 550 to 780 Wh L^−1^. In addition, the thickness of the Li metal layer and the presence of a Cu collector with lithium metal coating also influence the overall energy density, as can be seen from the downward shift of the curve in both cases.

We have also found that removing Li metal (density 0.53 g cm^−3^) from the cell configuration does not significantly improve performance in terms of gravimetric energy density, while it improves the volumetric energy density. The best condition appears to be that in which the Cu collector with the least thickness is considered.

Finally, the primary contribution of each cell component, measured in weight and volume percentages, is illustrated for configurations with Li metal foil both with and without Cu current collector.

**FIGURE 3:**
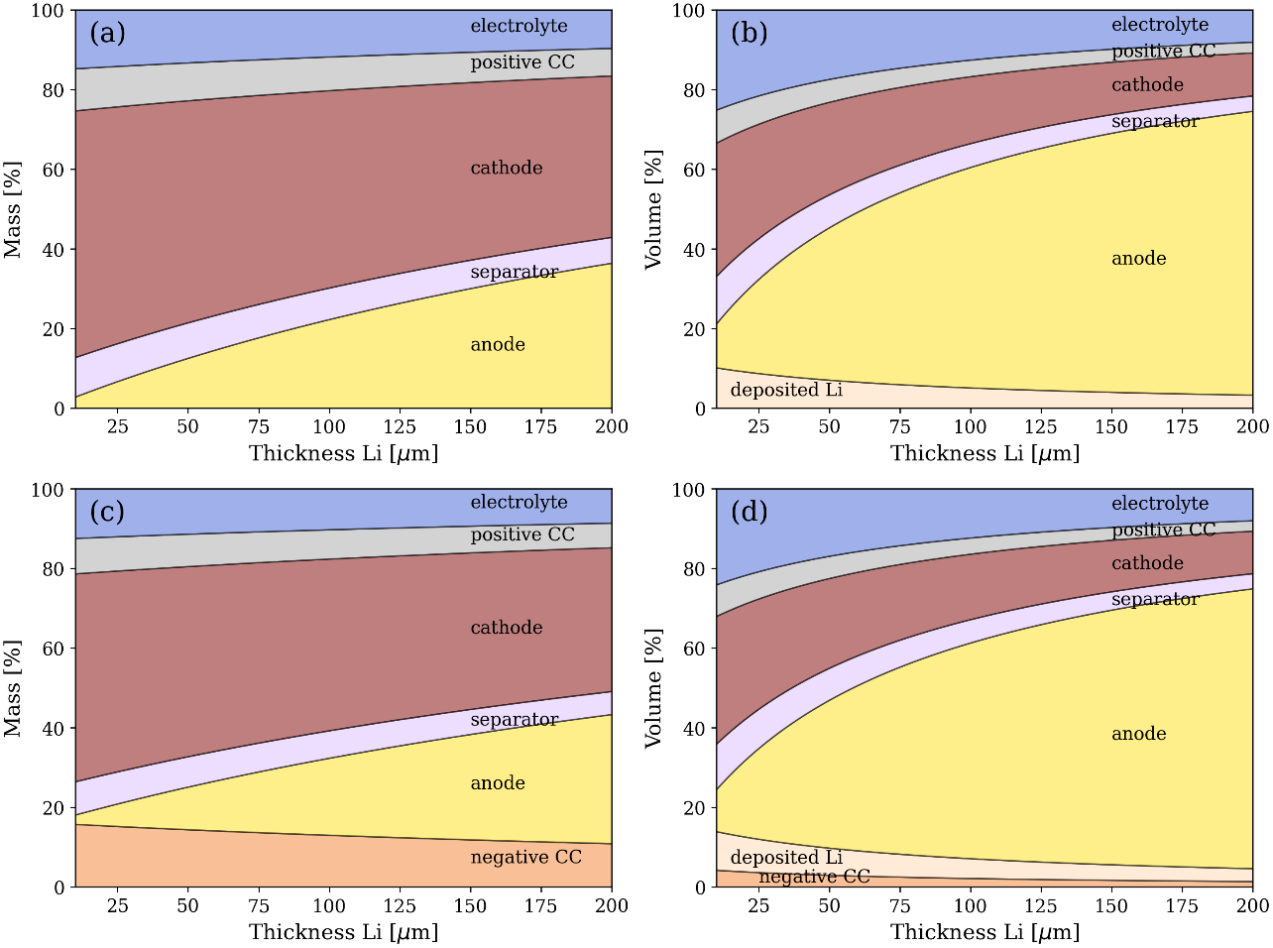
Li metal configuration without current collector (a and b) and with an 8 µm Cu current collector (c and d). Left column measured in mass (%), right column measured in volume (%).

Figure 3a shows that the positive electrode has a significant influence on the calculation of the total mass and, thus, on the gravimetric energy density of the cell. The contribution of the cathode remains dominant, although it decreases slightly with increasing Li layer thickness, just as the influence of the electrolyte (separator) decreases with increasing Li layer thickness. Conversely, the contribution of Li to the total weight of the cell increases with increasing Li thickness. In terms of volumetric energy density, the anode accounts for the largest share of the cell volume, as can be seen in Figure 3b. Its share of the total volume increases significantly with increasing of thickness.

After the anode, the cathode is the component with the second largest influence on the total volume, followed by the separator, then the current collector of the positive electrode, and finally the deposited Li.

The above observations also apply to Figure 3b,d, which show the mass fraction of each cell component when an 8 µm Cu current collector is included. In this scenario, the cathode continues to contribute the most to the weight, regardless of the thickness of the Li metal. However, it should be noted that the weight of the anode increases as the thickness of the Li increases. The contribution of the Cu current collector to the total weight is greater than that of the separator and the positive current collector (aluminum). However, the Cu current collector is hardly relevant for the volume calculation. The observations for the cell without current collectors still apply, with the anode, the cathode, and the separator being the main contributors to the volume.

The calculations performed show that an AFB configuration can lead to increased volumetric energy density at the cell level compared to an LMB. On the other hand, despite the use of thin 4–8 µm Cu current collectors, their relatively high density (8.96 g cm^−3^ compared to 0.53 g cm^−3^ for Li) significantly penalizes the gravimetric energy density. The latter would benefit from the development of lightweight lithiophilic current collectors (e.g., inactive polymer‐ or cellulose‐based films coated with a thin conductive lithiophilic layer)  to minimize the total weight. Moreover, the reduction in polarization caused by lithiophilic substrates due to the reduced real current density and reduced crystallization polarization could be offset by the additional cell weight, resulting in lower energy density values. Therefore, the thickness and weight of the lithiophilic coating or treatment of the current collector should be minimized to maximize cell energy density in an AFB cell configuration. The trade‐off between lower polarization and increased cell mass becomes the critical factor in the proper selection of lithiophilic current collectors, with consideration of the intended operating conditions of the system being of utmost importance.

## 3 Processes Affecting the Performance of Practical AFBs with Liquid Electrolytes

The development of anode‐free batteries has its roots in systems that use liquid electrolytes. For this reason, this chapter focuses on examining and discussing the most important phenomena associated with this type of anode‐free battery.

The performance, lifetime, and safety of AFBs depend crucially on the efficiency, morphology, and reversibility of the Li metal plating and stripping process. During the first charge, lithium metal nucleates on the current collector (CC, typically copper), which is covered by a native and electrochemically formed solid electrolyte interphase (SEI) [9]. The interface between the CC and the electrolyte is central to controlling the nucleation and morphology of the electroplated Li layer, as discussed in Section 3.2.

Successful nucleation requires overcoming an energy barrier that can be described using classical nucleation and growth theory based on Gibbs free energy. Thermodynamically, a higher overpotential (or current density) generally promotes higher nucleation density [10, 11], although temporal effects related to the evolving SEI are not captured by classical models. Electrodeposition follows two primary nucleation modes: instantaneous, in which nuclei form simultaneously, and progressive, in which new nuclei form and develop over time [12, 13]. These nucleation modes, together with interfacial energy, current density, and electrolyte composition, govern local deposition rates, Li morphology, and SEI evolution. The resulting morphology can range from a compact and uniform layer to a highly porous or dendritic. The high chemical reactivity of Li leads to the formation of an SEI that continuously evolves with cycling. Since virtually any freshly formed Li metal surface is covered with an SEI, this interface is the dominant factor in the performance of AFBs, influencing not only ionic transport and mechanical stability, but also the morphology and the reversibility of cycling.

**FIGURE 4:**
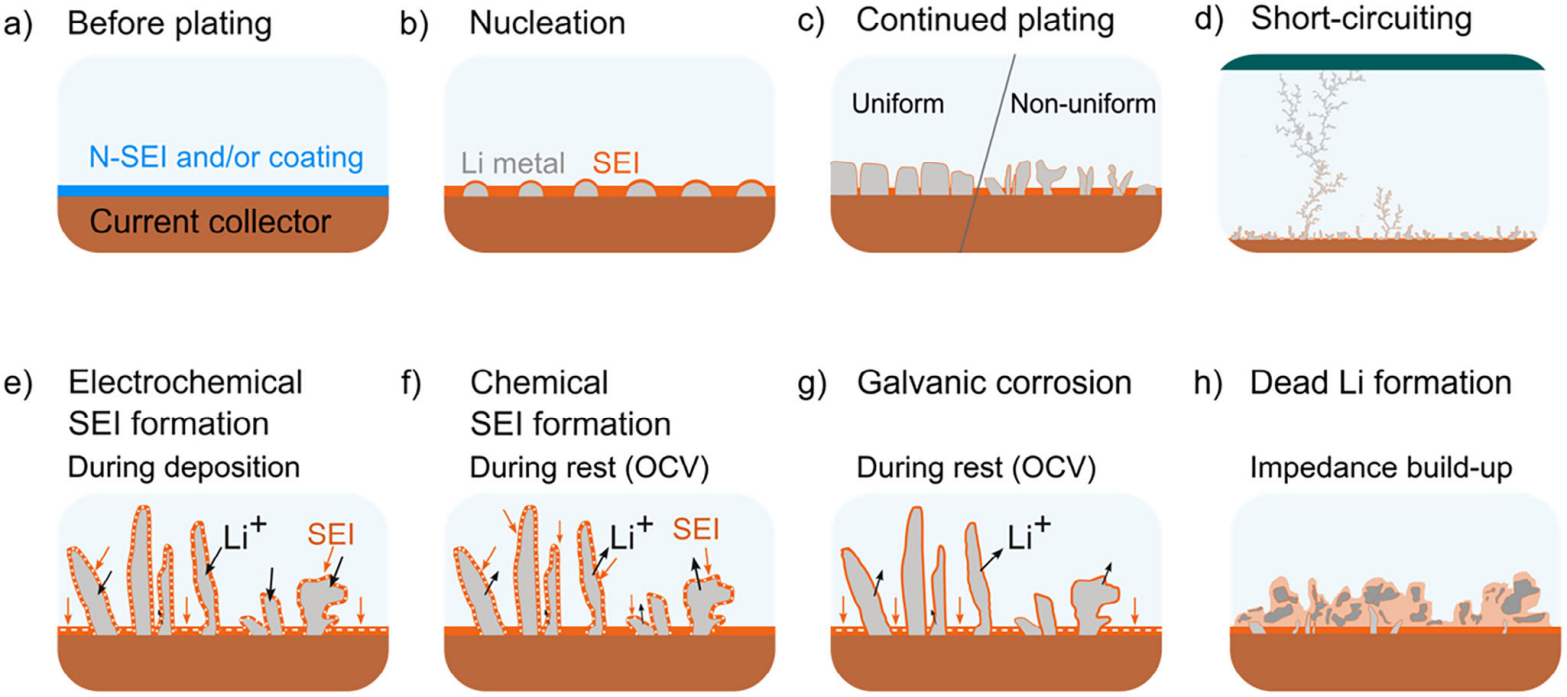
Schematic representation of lithium plating and the main degradation pathways in AFBs. (a) Before plating, the current collector is usually covered by a native SEI (N‐SEI) and/or an applied coating such as an artificial SEI or a lithiophilic interlayer. (b) During the first charge, nucleation occurs simultaneously with SEI formation. (c) With continued plating, the Li nuclei grow and coalesce, resulting in either uniform or non‐uniform Li morphology depending on interfacial and plating conditions. (d) Non‐uniform growth can lead to the formation of dendrites and short circuits between the anode and cathode (green). Such short circuits can be either transient (soft shorts) or permanent (hard shorts). (e) Electrochemical SEI formation occurs during Li electrodeposition when electrolyte reduction takes place at the metal/electrolyte interface. The black arrows represent the flux of Li^+^, the orange arrows represent the formation of SEI. The dotted line represents active SEI regions. (f) Chemical SEI formation occurs without net current flow in the circuit (open‐circuit voltage, OCV) and involves electrolyte reduction in conjunction with Li oxidation, resulting in thickening and compositional changes of the SEI. (g) Galvanic corrosion occurs at the OCV due to the Li oxidation in conjunction with a reduction of the electrolyte on the Cu surface. (h) Dead Li corresponds to inactive Li that no longer has electronic contact with the current collector. The accumulation of dead Li and the corresponding SEI leads to increased cell impedance and reduced electrochemical performance.

The main degradation pathways associated with Li plating and stripping in liquid electrolytes are summarized in Figure 4. In the following sections, we will discuss some important processes and strategies for improving the cell performance of AFBs.

### 3.1 Degradation and Failure Modes of AFBs

The biggest challenge with AFBs is maintaining capacity over extended cycling, which requires an average Coulombic efficiency exceeding 99.9%, something that has not yet been achieved for long‐term cycling in AFBs [14, 15, 16]. Even small inefficiencies become immediately apparent in anode‐free systems, as there is no excess Li is available to compensate for irreversible losses. Lithium is irreversibly lost through continuous SEI growth, galvanic corrosion at the Li/Cu interface, and the formation of electronically isolated dead Li. These processes are strongly coupled to the morphology of the deposited Li and the evolving properties of the SEI and ultimately determine capacity retention and cycle life. Therefore, improving capacity retention remains central to the development of high performant AFB systems.

#### 3.1.1 Short Circuiting

**FIGURE 5:**
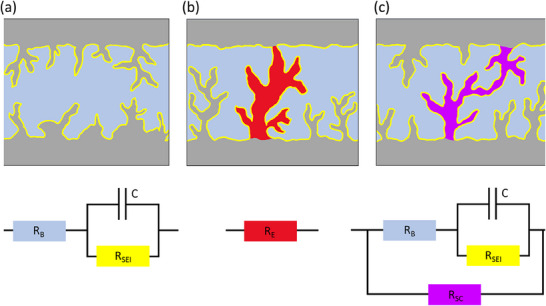
Illustrations and equivalent circuits for charge transport through the electrode–electrolyte interface (purely ion transport) (a), hard short circuit (electron transport) (b), soft short circuit (mixed electron and ion transport) (c). The electronic circuit elements represented are R_B_: the bulk electrolyte resistance, R_E_: electronic resistor from hard short, R_sc_: electronic resistor from soft short, R_SEI_: the SEI resistance and C: the SEI capacitance, a charge transfer resistor (R_ct_) defined as R_ct_ = R_SEI_ + R_B_. Reproduced with permission [17]. Copyright 2024, Royal Society of Chemistry.

Internal short circuits caused by non‐uniform Li plating represent a major degradation pathway in Li metal batteries and AFBs. A short circuit is an abnormal electrical circuit in which current flows through an unintended low‐resistance electronic pathway within the cell. In batteries with metal‐based anodes, such short circuits are typically classified as hard and soft shorts. Traditionally, research has tended to focus on mitigating hard shorts due to their immediate and often catastrophic effects. In this failure mode, Li forms a persistent metallic bridge between the electrodes, creating a low‐resistance electrical path comparable to a metal wire (Figure 5b). Although relatively rare, hard shorts can lead to a rapid increase in current, local heating, thermal runaway, and, in extreme cases, fire or explosion.

In contrast, soft shorts are transient and occur more frequently than hard shorts under standard battery cycling conditions. They involve a localized electrical connection between the electrodes that allows the coexistence of direct electron and ion transfer as well as interfacial reactions (Figure 5c). In equivalent circuit terminology, a charge transfer resistor (R_ct_ = R_SEI_ + R_B_), represented by the Randels equivalent circuit (Figure 5a), is parallel with another (less) resistive element defined as a short circuit resistor (R_sc)_, which allows electrons to flow through the shorting wire and be consumed by the surface reactions, i.e., SEI formation (Figure 5c). When the magnitude of R_ct_ is comparable to R_sc_, electronic and ionic charge transport coexist. Under such conditions, the electrochemical performance of a symmetrical cell may appear deceptively good, exhibiting low overpotentials and a rectangular voltage profile. In reality, however, little to no electrodeposition takes place; instead, the applied charge is predominantly consumed by excessive SEI formation. Illustrations and equivalent circuit models for a non‐shorted symmetric cell, a hard short, and a soft short are depicted in Figure 5 [17].

While hard short circuits can be predicted based solely on the shape of the voltage profile, soft shorts have proven more difficult to detect and measure in both liquid and solid electrolyte cells [17, 18, 19]. As early as 1990, Moli Energy identified soft shorts as a potential failure route for Li metal batteries [20]. Indeed, Albertus et al. suggested that numerous studies on stable lithium metal cycling are due to undetected soft shorts, which lead to artificially enhanced cycling stability [21]. The authors suggest that the transition to cells with a limited amount of excess Li could help identify soft shorts. Using coupled impedance spectroscopy, temperature‐dependent symmetric cell cycling, and operando NMR, Menkin et al. showed that soft shorts can form during AFB operation. The typical rectangular voltage waveform of symmetrical Li metal cells, often considered indicative of stable cycling, was instead attributed to soft shorts [17, 18].

Counihan et al. extended this analysis to solid‐state systems using temperature‐dependent impedance analysis [19]. They developed a computational model to describe the dynamics of these types of shorts, where the transient short triggered by Joule heating and chemical reactivity can be on the order of micro‐ to milliseconds. This demonstrates that ex situ results cannot be used to verify whether a cell has suffered a soft short during operation and that *in situ* measurements are essential for understanding this degradation mechanism. This concept has been supported by more recent studies [22].

These studies suggest that it is reasonable to assume that soft shorts can form in any battery configuration using metal anodes. In parallel, several papers have been published on the detection of soft shorts in pouch cells and battery stacks [23, 24]. These methods are designed for quality control on a pack level and need to be adapted for materials research, while the impedance methodology developed by Menkin et al. [17]. and Counihan et al. [19]. is aimed at basic research on single cells. We note that additional method development is required to enable operando quantitative detection of soft short circuits in commercial batteries.

#### 3.1.2 Inactive Li

The main cause of the low capacity retention in AFBs is the irreversible loss of active Li metal in the cell, referred to as inactive Li. This includes both Li^+^ compounds in the SEI, which form chemically on the Li metal (Figure 4f) and electrochemically during cycling (Figure 4e), and the formation of electronically isolated Li metal, typically referred to as dead Li (Figure 4h) [25, 26]. While the formation of SEI in liquid electrolytes cannot be avoided, controlling the SEI properties becomes paramount to satisfactory AFB performance. Better cycling efficiency is typically associated with a uniform and stable SEI [27, 28, 29, 30, 31, 32, 33, 34]. The SEI should be chemically and morphologically uniform, electronically insulating yet ionically conductive, mechanically stable, and insoluble in the electrolyte [35, 36]. Full and homogeneous coverage that effectively passivates both the Li metal and the current collector is essential to mitigate continuous electrolyte consumption and promote uniform deposition [37, 38]. Irregular microstructural growth has been linked to local inhomogeneities on the Li metal surface and within the SEI, leading to preferential deposition sites (known as “hot spots”) with high local current density [27, 33, 39, 40, 41]. These preferential deposition sites can form due to uneven transport properties in the SEI or due cracks in the SEI, where Li grows through protrusions caused by internal stresses beneath the SEI [42, 43, 44, 45].

Dead Li refers to Li that is no longer electronically connected to the current collector and is typically formed during the inhomogeneous stripping of Li microstructures (i.e., during discharge) [10, 26, 46, 47, 48]. Dead Li formation is attributed to the faster dissolution of Li metal at sites with low impedance, such as freshly deposited regions with a relatively thin SEI or areas where the SEI has ruptured [43]. Dachraoui et al. showed that dead lithium forms due to preferential lithium dissolution from the root of a dendrite [49]. Furthermore, it has been demonstrated that the thin, needle‐like dendrites often blamed for battery short‐circuiting tend to break down and form dead lithium [50]. The accumulation of dead Li not only leads to a direct loss of capacity but can also result in a highly tortuous layer that restricts mass transport and increases cell impedance, further accelerating capacity fade through premature voltage cut‐offs at the cathode. In addition, ongoing reactions at the electrically isolated Li structures lead to continuous SEI growth and continuous electrolyte consumption, even though these structures are not connected to the battery circuit [46, 47].

The chemical reactivation of inactive Li using soluble redox mediators has been explored as a strategy for recovering lost capacity. Redox shuttles such as iodide/triiodide (I^−^/I_3_^−^), bromide/tribromide (Br^−^/Br_3_^−^), polysulfide, and 2,2,6,6‐tetramethylpiperidine‐1‐oxyl (TEMPO) couples can oxidize inactive Li metal or dissociate Li_2_O to Li^+^, after which the reduced mediator is re‐oxidized at the cathode to reincorporate Li into the cathode [51, 52, 53, 54]. Although these systems can temporarily improve capacity retention, they suffer from parasitic side reactions, self‐discharge during rest, and poor selectivity towards different SEI components, which likely leads to accumulation of other SEI species such as LiF, LiOH, and ROLi [55]. The preferential removal of Li_2_O from the SEI, which has previously been associated with higher cycling stability in lithium metal batteries [16], could even be detrimental over extended cycling.

#### 3.1.3 Li Corrosion Processes

Corrosion processes represent another significant degradation mechanism in AFBs. Li metal is highly reactive and susceptible to multiple corrosion mechanisms in liquid electrolytes. Li corrosion refers to both chemical corrosion—the continuous formation of SEI on Li metal, accompanied by Li oxidation when no external current is applied (Figure 4f)—and galvanic corrosion, in which electrolyte reduction occurs at the current collector surface (Figure 4g). Both processes consume active Li, leading to capacity loss and increased cell impedance.

In general, electrochemical corrosion occurs when coupled anodic and cathodic half‐reactions take place at electronically connected surfaces. In AFBs, the anodic reaction involves the oxidation of Li metal: Li^0^ → Li^+^ + e^−^. This must be balanced by a cathodic reaction involving the reduction of the liquid electrolyte, e.g., the reduction of the organic solvent such as EC or DMC, which results in SEI formation. The corrosion rate is determined by the net rate of these oxidation‐reduction reactions, which depends on the electrochemical potential established across the metal/electrolyte interface.

Galvanic corrosion is particularly important in AFB configurations where Li is deposited directly on a metal current collector such as Cu. Under these conditions, electronic and ionic connections between the two metals can establish a local galvanic couple that accelerates lithium oxidation [56, 57, 58]. The ionic connection is maintained not only through the liquid electrolyte but also across the SEI. To suppress Li dissolution, a stable and uniform SEI must form on the plated Li, making electrolyte design and SEI engineering critical for mitigating this process [56, 57, 58]. One of the roles of the SEI is in AFB is therefore to inhibit Li corrosion.

One strategy is to apply an artificial SEI that chemically passivates both interfaces (Li and Cu) to reduce parasitic reactions with the electrolyte [59]. Furthermore, the morphology of the Li deposits has a strong influence on the corrosion rate: a smoother, more uniform plating results in greater surface coverage with Li, minimizing the areas where Cu is in contact with the electrolyte [57, 60, 61]. The critical conditions for galvanic corrosion are met when Cu is partially covered with Li, which is typical for the beginning of the plating stage and the end of the stripping stage in AFBs. Therefore, anode‐free cells resting in the last stages of discharge or in the first stages of charging could suffer rapid Li loss due to galvanic corrosion.

Several studies have explored the use of tailored cycling protocols to mitigate capacity losses, reporting apparent partial capacity recovery during resting [57, 60, 62, 63, 64]. Merrill et al. investigated calendar ageing in the charged state (i.e., with plated Li) and observed both capacity fade and apparent recovery. This was evidenced in a drop in CE after the rest, followed by efficiencies of over 100% in subsequent cycles [63], which they attributed to SEI healing. Zhang et al. also reported a similar apparent capacity recovery after resting cells in a discharged state (i.e., without plated Li), which was attributed to the dissolution of SEI shells around inactive dead Li, allowing them to be electronically connected during subsequent cycling [62]. The mechanisms underlying this recovery of inactive Li remain unclear, and we stress that this is likely related to the dissolution of soft shorts, which have been shown to result in CE greater than 100% [19, 48, 65, 66]. Although no direct evidence has been found for the contribution of dead Li to the formation of soft shorts (or vice versa), the authors suggest that dead Li and soft shorts often coexist, as both degradation modes result from needle‐like Li dendrites—and indeed, both worsen as Li corrosion intensifies. As a result, the signatures of dead Li and soft shorts are often recorded in proximate measurements. Therefore, it is likely that efficiencies above 100% (which typically result from the collapse of soft shorts) are mistakenly attributed to the reversibility of dead Li [49].

#### 3.1.4 Bridging the Gap Between Fundamental and Practical Diagnostics: Characterization of Degradation in AFBs

The ability to characterize degradation processes in AFBs remains a central challenge for both scientific understanding and practical application. As summarized above, AFBs suffer from spatially heterogeneous Li plating, buried and evolving interfaces, and dynamic SEI formation. Although significant progress has been made in understanding these phenomena with the help of operando and high‐resolution techniques, there remains a gap between fundamental insights and diagnostics relevant for real‐world applications. To bridge this gap, not only spatial and temporal resolution are required, but also cross‐validated metrics and standardized descriptors that can link nanoscale degradation features to macroscopic cell performance.

Fundamental high‐resolution techniques remain indispensable for post‐mortem analysis and benchmarking in AFBs, although they are often destructive. Scanning electron microscopy (SEM) is commonly used to analyze Li morphology, while X‐ray Photoelectron Spectroscopy (XPS) and time‐of‐flight secondary ion mass spectrometry (ToF‐SIMS) are used to investigate the chemical composition of the SEI and CC surfaces. However, ex situ methods are often qualitative and prone to artifacts during sample preparation, making cross‐study comparisons difficult. Recent calls in the literature advocate for standardized imaging protocols for SEM imaging (e.g., porosity, coverage, grain size) to improve the reproducibility of reporting [67]. Despite their limitations, these techniques play a crucial role in correlating structural and chemical features with electrochemical performance and in validating results from operando and macroscopic measurements.

To overcome the limitations of post‐mortem sample handling, cryo‐electron microscopy has emerged as a key technique for investigating SEI formation on alkali metals. Cryogenic sample preparation using liquid nitrogen enables cross‐sectioning of samples without decomposing air‐sensitive phases [68, 69]. For example, cryo‐focused ion beam scanning electron microscopy (cryo‐FIB‐SEM) was used by Janek et al. to characterize the microstructure of Li and Na metal grains in anode‐free solid‐state batteries [70]. These techniques preserve native interfaces that would otherwise degrade during sample preparation under ambient conditions. Cryo‐transmission electron microscopy (cryo‐TEM) allows direct imaging of the SEI nanostructure, identification of crystalline and amorphous regions of the SEI using selected area electron diffraction (SAED), and elemental mapping using energy dispersive spectroscopy (EDS) [16, 37, 71, 72]. Additionally, cryo‐TEM has also been used to investigate the effect of corrosion on Li microstructures and SEI [57, 60]. Furthermore, depth‐resolved cryogenic XPS can be used to determine the heterogeneous structure of the native SEI without drying the sample beforehand, which could otherwise alter its chemical composition [73].

For quantification, titration‐based methods such as titration gas chromatography (TGC) are powerful ex situ tools for quantifying inactive lithium, enabling differentiation between metallic dead Li and Li‐ions within the SEI. The seminal work by Fang et al. established the approach for quantifying dead Li on Cu CC, and, subsequently, protocols were developed to quantify different SEI components such as Li_2_O using similar strategies [16, 74, 75]. Similarly, solid‐state nuclear magnetic resonance (ssNMR) spectroscopy can identify different components of the SEI, but accurate quantification of different SEI components on Li metal and the current collector remains difficult due to sample handling and the low spectral resolution of ^7^Li NMR [9, 76, 77].

In situ and operando techniques are critical in tracking dynamic degradation in AFBs. In situ NMR is a non‐invasive technique that provides both quantitative and temporal information on capacity losses due to various degradation processes in AFBs (corrosion, SEI and dead Li formation) [56, 78, 79]. In situ microscopy techniques, including optical microscopy, in situ TEM, and environmental SEM, enable real‐time observation of lithium nucleation and growth, dendrite propagation, void formation, and SEI formation [78, 79]. Optical microscopy has proven particularly valuable in linking specific voltage signatures to degradation processes during plating and stripping and correlating impedance buildup with the formation of dead Li and voids.

However, many in situ studies on liquid‐based systems rely on modified or semi‐flooded Swagelok‐type cells, which are of limited relevance to practical battery geometries. The further development of nondestructive in situ techniques that can operate on realistic pouch cell configurations is therefore crucial for translating fundamental insights into degradation models. Among spatially resolved techniques, in situ X‐ray tomography has emerged as a powerful tool for three‐dimensional visualizing of lithium plating, void formation, and electrode thickness evolution [80, 81, 82, 83, 84]. Recent developments have enabled operando 3D imaging of Li morphology and CC interfaces directly in pouch‐cell formats. Although tomography often suffers from limited temporal resolution, it offers the potential for integration into battery fabrication lines for quality control and process optimization [85, 86]. Furthermore, acoustic methods have emerged as a cost‐effective and non‐invasive tool with significant potential for in situ monitoring of chemical and mechanical processes in electrochemical power devices [87, 88, 89]. Chang et al. investigated the influence of formation cycles and different electrolytes on the long‐term cycling stability of anode‐free cells using operando acoustic transmission. They showed how the mechanical response of a commercial pouch cell changed, likely due to the build‐up of degradation products (SEI and dead Li), before any signatures from electrochemistry appeared [89]. Thus, the technique demonstrates potential for early detection of failure in commercial AFBs.

To strengthen interpretability and cross‐compatibility between academia and industry, it is a priority to establish quantitative correlations between spatially resolved techniques and practical, scalable diagnostic methods such as EIS and voltage‐time analysis. Here, advanced techniques and modelling frameworks that have already been developed must be combined with detailed electrochemical analysis to translate indirect electrochemical signatures of degradation into mechanistic insights [90, 91].

### 3.2 The Role of the Current Collector (CC)

The electrodeposition of Li metal occurs at the surface of the current collector (CC). Understanding this process, from nucleation to growth, and optimizing the interfacial properties of CCs are crucial for improving the performance and stability of AFBs. Surface heterogeneity has a strong influence on the nucleation barrier for deposition, as variations in local impedance, surface composition, topography, or microstructure can lead to uneven nucleation and non‐uniform growth. Higher Li nucleation density promotes more uniform and compact deposition, minimizing active material loss and improving cycling stability [92]. The nucleation barrier (experimentally reflected by the overpotential) is influenced by the interfacial energies between Li and CC, which depend on factors such as lithiophilicity, lattice matching, and the SEI layer forming on the CC [12].

#### 3.2.1 Copper CC

We focus mainly on copper CC, the most commonly used anode substrate in LIBs and AFBs with liquid electrolyte. As with other beyond‐Li‐ion battery technologies, the development of AFBs relies heavily on the extensive knowledge and design principles established for LIBs. Accordingly, the Cu CC on the anode side was adopted directly from LIB systems. Copper offers high electrical conductivity, does not react with Li metal, and is generally considered electrochemically inert under both reducing and oxidizing conditions in LIBs. However, the Cu foils used in LIBs were developed to prevent the formation of Li alloys, maximize electronic conductivity, and improve the adhesion of the cast graphite electrode, not for Li metal deposition. These foils are usually roughened, which may promote the growth of Li dendrites, posing a problem for AFBs. Moreover, the heterogeneous native oxide layer on commercial Cu foils introduces spatial variations in impedance, resulting in uneven electrodeposition [9, 93, 94]. The surface chemistry of Cu has therefore emerged as a critical factor in controlling Li plating behavior in AFBs. Understanding Cu surface degradation and its reactions with the electrolyte is essential for improving the overall cyclability of AFBs.

The degradation of Cu CCs has received limited attention to date, as the thermodynamic equilibrium potential for Cu dissolution (3.92 V vs. Li^+^/Li) lies well above the operating voltage of the anode in standard LIBs (typically 0.0–1.5 V). However, under conditions such as over‐discharge—a common failure mode in stacked pouch cells—the potential on the anode can rise drastically, leading to oxidation and dissolution of Cu [95, 96]. This is concerning because the copper ions formed can later redeposit as Cu metal on the anode and even on the cathode, potentially forming Cu dendrites [97].

In conventional LIBs, the graphite coating on the Cu can act as a diffusion layer and suppress Cu dissolution [98]. In AFBs, where such protection is lacking, one might expect the bare Cu surface to be more susceptible to corrosion. However, the opposite trend was observed, with carbon‐coated copper tending to corrode more [99]. Although the cause of the increased corrosion on the carbon‐coated copper is not yet understood, this finding is encouraging as it suggests that copper corrosion in AFBs is not more severe.

Although Cu oxidation below 3.92 V vs Li^+^/Li is thermodynamically unfavorable, trace water and impurities in LiPF_6_‐based electrolytes can lead to significant Cu corrosion and potential safety issues, even at open circuit potential [100]. To mitigate this, commercial lithium‐ion cells are typically charged to 3.3–3.5 V (SOC30%) prior to shipment, driving the anode potential more negative and thus away from the Cu dissolution range [96, 101]. To our knowledge, there are no equivalent preconditioning protocols for AFBs. Therefore, dedicated strategies for corrosion protection in AFBs remain an open and important area for future research.

Apart from Cu oxidation processes, the reactivity of Cu in organic electrolytes and the influence of its native oxide layer on Li plating have been largely overlooked. However, recent studies have revived interest in Cu surface chemistry as a potential approach to controlling Li nucleation and growth in AFBs [9].

Under ambient conditions, Cu oxidizes readily and forms a heterogeneous native oxide layer. Commercial battery‐grade Cu foils are passivated by a mixture of Cu oxides and hydroxides, and controlled reoxidation to form a more homogeneous oxide layer has been shown to be beneficial for more uniform Li plating. [9, 102] The oxidation of Cu proceeds in three main stages: First, a Cu_2_O layer forms, followed by a layer of metastable Cu hydroxide Cu(OH)_2_, which later transforms into the more stable CuO [103]. The total oxide thickness is typically 2–15 nm on single‐crystal Cu and up to 20 nm on polycrystalline Cu foil. On rough battery‐grade foils, a mixed Cu_2_O/CuO layer usually forms, while on very smooth Cu surfaces, a predominant Cu_2_O layer may form [104].

Yoon et al. suggest that the presence of heterogeneous Cu_2_O on the Cu CC leads to the formation of Li_2_O upon initial plating, introducing interfacial heterogeneity with both lithiophilic (Cu) and lithiophobic (Li_2_O) regions that promote uneven Li nucleation and growth [93]. However, Hobold et al. showed that for Li plating and stripping on Cu CCs across a variety of electrolytes, high CE correlates most strongly with high Li_2_O content in the SEI, rather than with LiF, a commonly proposed CE descriptor for Li metal systems. Moreover, the ionic conductivity of Li_2_O on Li metal is an order of magnitude higher than that of LiF, which could explain its beneficial effect on reversible cycling [16, 105]. Crucially, cryo‐TEM analysis revealed that high‐CE electrolytes tend to form a uniform, highly ordered Li_2_O layer at the outer SEI‐electrolyte interface, while low‐CE systems form dispersed Li_2_O nanoparticles. In summary, these studies show that uniform spatial distribution of Li_2_O in the SEI is beneficial, while the heterogeneous formation of Li_2_O resulting from localized Cu_2_O reduction impairs the uniformity of Li plating in anode‐free systems.

When the Cu surface comes into contact with a LiPF_6_‐based electrolyte, a native SEI‐like layer (N‐SEI) consisting mainly of LiF, CuF_2_ and Cu_x_O forms spontaneously, even without applied potential [9, 16]. The breakdown of the LiPF_6_ electrolyte is unique to Cu and is attributed to the catalytic decomposition of HF on the copper surface, resulting in the formation of LiF [106]. The composition and homogeneity of this N‐SEI evolve during storage at open‐circuit potential, with the rest time influencing the morphology of the subsequently plated Li. Freshly formed N‐SEI (approximately 1 h after assembly) promotes dendritic Li growth, while aged samples (18 h of rest time) exhibit a flatter Li morphology. This effect has been attributed to either thickening or rearrangement of the N‐SEI while resting in the electrolyte [9]. However, thicker inorganic layers on Cu can also increase the plating overpotential, leading to spatially inhomogeneous nucleation and island‐like Li growth [107].

Investigation of the N‐SEI formed on Cu during the rest period (typically >2.5 V vs. Li^+^/Li) and the electrochemical SEI suggests a different chemical composition than the SEI formed on Li metal anodes [9]. Upon polarization (<2.5 V vs Li^+^/Li), the chemically formed N‐SEI and copper oxides undergo electrochemical reduction, resulting in the formation of the electrochemically formed SEI. The electrochemically formed SEI consists of N‐SEI as the primary layer, reduction and lithiation products of Cu oxide (Li_x_CuO, Li_2_O, and LiOH), and solvent reduction products (see Table 2 for the formation reactions and potential of typical SEI components). Carbonate solvents are reduced at potentials below 0.5 V vs. Li^+^/Li, producing short‐chain organic species such as lithium ethylene decarbonate (LEDC) and poly(vinylene carbonate), while the reduction of fluorinated solvents such as fluoroethylene carbonate (FEC) generates LiF and Li_2_CO_3_ [108, 109, 110]. The distribution of these SEI compounds (LiF, LiOH, Li_2_O) with relatively low ionic conductivity determines Li nucleation and growth rather than the electronically conducting compounds (Cu, CuO, Li_2_C_2_) [9]. The authors therefore hypothesized that the reason for the preferential plating lies in the considerable variance in the ionic conductivity of the SEI components, which leads to a heterogeneous current density distribution on the Cu surface and thus to heterogeneous Li coverage and morphology [9].

**TABLE 2:** The precursors (electrolyte/electrode component), products (SEI component) and formation potentials of SEI compounds on Cu.

Electrolyte/ electrode component	SEI component	Formation potential vs Li^+^/Li [V]	Formation reaction and notes	Refs.
LiFP_6_, HF, free F^−^	LiF	3‐2.5	2Li^+^ +2e^−^ + CuF_2_ → Cu + 2LiF	[9]
Li_2_O			Li_2_O + HF → LiF + LiOH	[9]
LiPF_6_ (in EMC)	LiF	1.2		[108]
Cu_x_O	Li_2_O	<2.0	Cu_2_O + 2 Li → Li_2_O + 2 Cu CuO + 2 Li → Li_2_O + Cu	[9, 93]
	Li_2_O, Li_z_CuO, nano Cu	1.1, 1.2	Reduction, Irreversible Cu_x_O lithiation and Cu_2_O formation	[111, 112, 113]
Carbonate solvent	Li_2_CO_3_		Solvent (DMC/PC/EC) reduction	[114]
EC, EMC PC	Lithium ethylene di‐carbonate (LEDC)/ lithium propylene di‐carbonate (LPDC) and ethylene gas	0.8 (EC, EMC) 1.0 (PC) 0.3 (EMC)	Electrochemical reduction, lithiation and polymerization	[108, 109, 110]
FEC	LiF, poly(VC), Li_2_CO_3_,	1.1		[109]
DME		0.82, 1.5		[108, 115]
LiDFOB		1.86		[108]
LiTFSI	LiF	1.33, 1.4, 1.6		[108, 111]
LiFSI	LiF	1.12		[108]
Li_2_O	Li_2_O_2_, LiO_2_		Li_2_O oxidation on Li metal	[9, 111]
Li_2_CO_3_	Li_2_C_2_	−0.5 on Li metal	Li_2_C_2_ formation on Li metal	[9, 111]
LEDC	Li_2_CO_3_, CO_2_, Li_2_O		Thermal decomposition, reaction with water traces	[109]

Taken together, these results suggest that the relative abundance and distribution of SEI compounds, rather than the presence of a single compound such as Li_2_O, govern the uniformity of the interface and Li morphology. This highlights the importance of understanding and controlling the chemistry of the Cu surface, either through pretreatments or by developing preformed artificial SEIs, to improve Li wetting and achieve more homogeneous deposition.

#### 3.2.2 Modification of CCs

The large number of publications on the effects of CCs on Li deposition demonstrates the importance for AFB performance [116, 117, 118, 119]. Common current collectors such as Cu and Ni suffer from uneven and dendritic electrodeposition. Although a mechanistic understanding of Li electrodeposition at the CC surface is extremely important for controlling this process and progress has been made in improving performance, the current problems are still significant for enabling the manufacture of reliable AFBs with liquid electrolytes. In particular, the lithiophobic nature of Cu CC (i.e., copper metal naturally covered with Cu oxides) remains the biggest challenge. Therefore, modification of the current collector surface has become another intensively investigated research direction.

The evolving nature of the Cu surface and the ambiguous effects of various Cu surface treatments have motivated the use of protective coatings, which are expected to improve Li–Cu interfacial wetting and promote more stable cycling behavior. However, the need for such coatings also poses a challenge for the commercialization of AFBs, as their additional volume and mass reduce the overall energy density and thus limit practical implementation.

The modification strategies can be classified into two main groups according to their operation mechanisms: (1) electron‐conducting metal and alloy coatings to reduce Li nucleation overpotential/barrier (i.e., improving Li‐Cu solid‐solid wetting) and (2) ion‐conducting inorganic and polymer coatings to improve and even Li‐ion transport across the Li/electrolyte interface (i.e., artificial SEI). However, recent studies have shown that these systems are more complex. For example, when using a thin tin and strontium coating, the improved reversibility is not primarily due to improved lithium deposition morphology or reduced lithium deposition. Instead, the alloying layer enhances reversibility by improving interfacial transport, particularly towards the end of lithium dissolution [120, 121, 122, 123, 124].

The lithiophilic layers are typically composed of different type of metals (e.g., Ag and Au), metals alloys such as CuZn or nitrides, or metal oxides (such as ZnO and CuO) [125, 126, 127, 128]. Other studies have developed 3D Cu current collectors to ‘trap’ Li dendrites and prevent short circuits [129]. Some studies have combined these approaches and developed pre‐treated, lithiophilic, 3D Cu current collectors [130]. Examples of the use of lithophilic coatings in sodium and solid‐state anode‐free batteries are discussed in more detail in Sections 4 and 5.

It has been demonstrated that metals that alloy with Li improve the deposition and performance of anode‐free cells, such as thin layers of Zn, Al, Ag, Mg or In [131, 132, 133, 134, 135, 136]. Other examples include alloying interlayer materials such as sputtered Au, chemically pre‐lithiated Li‐Sn alloy, a cast Zn layer, and Ge‐based layers [137, 138, 139, 140]. For example, Liu et al. induced atomically distributed Zn defects on commercial Cu foil by magnetic sputtering of Cu_99_Zn. Due to its improved lithiophilic properties, the Cu_99_Zn substrate exhibited lower overpotential during plating and maintained a CE of over 98% at a Li plating capacity of 10 mAh cm^−2^ [141].

In addition, carbon‐based layers and hosts have also been explored as suitable CCs for AFB. Several examples can be found in literature, ranging from relatively simple methods based only on carbon‐based materials such as graphene particles to more complex strategies to improve Li plating/stripping [142]. Some of these approaches involve the addition of metals in nanoparticles form or nanowires together with carbonaceous materials to reduce the nucleation overpotential [143, 144, 145]. Typically, carbonaceous coatings lead to more uniform lithium electrodeposition, as lithium first lithiates the carbon phase, reducing the nucleation overpotential for subsequent metal plating.

An important aspect on the practicality of AFBs is stoichiometric or N/P ratio (defined as the capacity of the negative electrode to the positive electrode, that is the Li inventory of the cathode) when the lithiophilic material contributes to capacity. Table 3 gathers three mains possible scenarios of the balance between the anode and cathode. The most desired scenario is when N/P<1 (ultimate goal N/P≪1) resulting in a two‐stage process starting from (i) lithiation of the lithophilic coating and (ii) continued electrodeposition of Li metal on the fully lithiated phase. In this scenario, the amount of lithiophilic coating is minimum to increase the capacity of the cell, however, it will also introduce typical problems associated with alloy anodes, such as pulverisation due to large volume changes and rapid degradation. Ultimately, the addition of a significant mass and volume of coating will also result in a decrease in energy density, as we demonstrated in Section 2.

**TABLE 3:** Stoichiometric ratio between cathode and lithiophilic coating in AFB.

Scenario	N/P ratio (cathode Li inventory / lithiophilic material capacity)	Capacity contribution by the lithiophilic coating
Uncoated CC	0	None
CC coated by lithiophilic material (e.g., Zn etc.)	<1 (Li deposition onto CC)	Minimal
	1 (no Li deposition onto CC)	Moderate
	>1 (no Li deposition onto CC)	Higher

### 3.3 Effect of Electrolytes on the Performance of AFBs

As with lithium‐ion and lithium‐metal batteries, one of the most important ways to improve the cyclability of anode‐free batteries is to carefully adjust the composition of the liquid electrolyte. Although electrolytes do not participate in the charge and discharge reactions, they are very important for AFB performance as they play a pivotal role in the formation and stabilization of electrode‐electrolyte interfaces. Therefore, the most relevant results showing the impact of electrolyte formulation on AFB performance are discussed.

A standard liquid electrolyte consists of one or more polar aprotic non‐aqueous solvents, lithium salt, and potentially some additives to enhance performance. While recent studies have demonstrated that the carbonate‐based electrolytes used in typical LIBs are not necessarily ideal for Li metal anode cells, they serve as a starting point for further modifications to improve the performance of Li metal anodes and enable high‐voltage Li batteries with high energy density.

A variety of solvents have been employed in LMB, including carbonates, ethers, sulfones, and nitriles, all of which have their benefits and drawbacks. Carbonate‐based electrolytes are typically a blend of two categories of solvents: cyclic (including ethylene carbonate (EC), propylene carbonate (PC), and fluoroethylene carbonate (FEC)) and linear (including ethyl methyl carbonate (EMC), dimethyl carbonate (DMC), and diethyl carbonate (DEC)). Cyclic carbonates are typically included in electrolyte blends as they provide better stability on the cathode surface, strong solvation of lithium salts and good passivation of the graphite anode surface. Studies have shown that the SEI generated is generally dominated by the reduction products of the cyclic carbonate used rather than by reduction products of linear carbonates [109].

Carbonate‐based electrolytes typically result in a rigid SEI that is prone to fracture and thickening, resulting in an uneven interface that is susceptible to dendrite growth and active material loss. Ether‐based electrolytes (e.g., dimethoxyethane (DME) or 1,3‐dioxolane (DOL)) are known to improve the performance of Li metal anodes in terms of cycle life, Li metal plating, and CE due to the highly flexible SEI formed. However, the instability of ethers at high potential greatly limits their application in high energy density cells with high‐voltage cathodes such as NMC [109].

The main strategies for the development of liquid electrolytes for AFB are quite similar to those of LIB and LMB and aim at the stabilization of the anode/electrolyte interface by SEI design. Several strategies utilize cell components that exhibit strong SEI forming ability, namely: (i) combination of solvents, functional additives, and Li salts, (ii) highly concentrated electrolytes, and (iii) fluorinated compounds. In addition, the safety aspects are addressed by flame retardants and non‐flammable components. However, compared to LIBs and LMBs, the development of AFBs is not yet mature and poses major challenges as it is hampered by the high reactivity of liquid electrolytes towards Li metal, absence of a host material, and the zero excess of available Li in the system. Nevertheless, several groups have reported promising results in optimizing the liquid electrolyte formulations.

The Li salt selected for the electrolyte contributes significantly to the anion decomposition products that make up the SEI. For example, some Li salts are preferentially reduced during the first Li plating cycle, while others only begin to decompose after the formation cycle is complete. Webber et al. compared seven electrolytes based on a blend of FEC: DEC (1:2 vol.) with different Li salts compositions and concentrations and demonstrated that the dual‐salt LiDFOB and LiBF_4_ electrolyte had the best performance of all electrolytes tested, with a capacity retention of 80% after 90 cycles [146]. This electrolyte is considered the benchmark electrolyte for AFBs.

**FIGURE 6:**
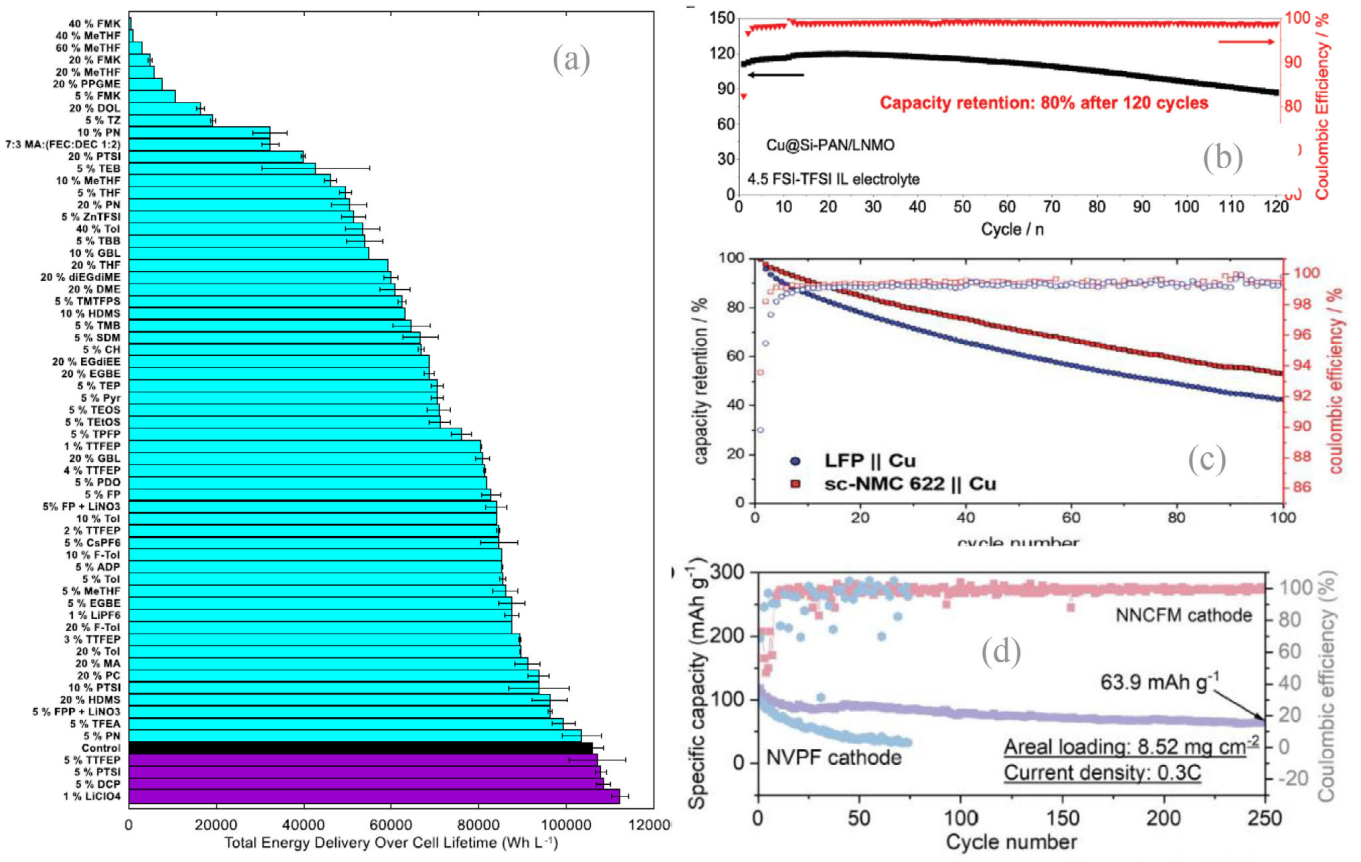
(a) Total energy delivery over 140 cycles for anode‐free cells with NMC811 cathode in 65 electrolyte mixtures tested by Dahn et al., demonstrating a strong effect of electrolyte nature and composition on the performance of AFBs. The cycling was performed at 40ºC between 3.55–4.40 V at 0.2C/0.5C. Average and range of pair cells is shown when available. Reproduced with permission [147]. Copyright 2021, IOP Science. (b) Cycling stability of the LNMO anode‐free batteries with Cu@Si‐PAN current collector and 4.5 FSI‐TFSI IL electrolyte. Reproduced with permission [148]. Copyright 2022, Wiley‐VCH. (c) Capacity retention and CE for LFP (blue) and sc‐NMC622k (red) on p‐Cu cell with P1222‐FSI/LiFSI electrolyte at C/2 (1.75 mA cm^−2^) at 50°C. Reproduced with permission [149]. Copyright 2016, Royal Society of Chemistry. (d) The cyclic stability of anode‐free Al‐NNCFM (O3‐NaCu_1/9_Ni_2/9_Fe_1/3_Mn_1/3_O_2_) and Al‐NVPF (Na_3_V_2_(PO_4_)_2_F_3_) full cells with 3Å zeolite modified electrolytes. Reproduced with permission [150]. Copyright 2022, Wiley‐VCH.

Dahn et al. published a very systematic work describing results of screening of 65 carbonate‐ and ether‐based electrolytes in Cu/NMC811 pouch cells cycled at 40°C with 0.2C/0.5C charge/discharge rates between 3.55–4.40 V [147]. In an earlier report by the same group, a dual‐salt electrolyte containing 0.6 M LiDFOB and 0.6 M LiBF_4_ in FEC and DEC (1:2 vol. ratio) was used as a control electrolyte system with a dramatically different composition of the anode SEI formed in LiPF_6_ based electrolytes [146]. Based on the total energy delivered over 140 cycles, only four electrolytes, namely tris(2,2,2‐trifluoroethyl) phosphate (TTFEP), p‐toluene sulfonyl isocyanate (PTSI), 1,5‐dicyano pentane (DCP), and LiClO_4_ showed a marginal improvement over the baseline, while the other electrolytes were not competitive (Figure 6a).

While maximizing battery performance is crucial, safety, sustainability, and cost must also be considered. For example, the use of perchlorates poses fire and explosion hazards, while fluorinated additives and solvents, particularly per‐ and polyfluoroalkyl substances (PFAS), are known for their toxicity, significant environmental impact, and high cost.

High‐concentration electrolytes (HCE) show promise for AFB, as they are reported to stabilize the Li/electrolyte interface by forming a denser SEI and modifying the lithium‐ion solvation shell to improve cation transport. For example, a very common HCE employs a high concentration of lithium bis(fluorosulfonyl)imide (LiFSI) in dimethoxyethane (DME), which leads to the formation of a novel solvation structure containing contact ion pairs (CIPs) and cation‐anion aggregates (AGGs) instead of solvent‐separated ion pairs (SSIPs) that are common in low concentration electrolytes [151]. This unique solvation structure has been shown to contribute to the formation of a robust inorganic SEI. However, the high salt concentration also results in high viscosity and high cost. To circumvent these issues, the concept of localized high‐concentration electrolyte (LHCE) was introduced [152, 153].

LHCE is based on the idea of introducing a co‐solvent, known as a diluent, which hardly solvates Li ions and thus reduces the overall concentration of the lithium salt while maintaining the HCE solvation structure. Commonly used diluents include fluorinated ethers such as bis(2,2,2‐trifluoroethyl) ether (BTFE) and 1,1,2,2‐tetrafluoroethyl 2,2,3,3‐tetrafluoropropyl ether (TTE). These diluents successfully reduce the viscosity of the HCE while maintaining its advantages. However, the high cost of BTFE and TTE limits their use in practical cells [150]. For example, NMC622/Li metal and anode‐free (NMC811 vs Cu) cells using a LHCE [LiFSI]:[DME]:[TTE] ratio of 1:2:1.2 demonstrated a prolonged cycle life and ultra‐fast charging capabilities [154]. However, studies on LHCE in AFBs are scarce, and critical issues such as oxidative stability of ether‐based solvents, corrosion of the current collectors, formation of soft shorts and sustainability and safety of the diluent need to be investigated before LHCEs can be considered for commercialization [154, 155, 156].

Liang et al. developed a non‐flammable ionic liquid electrolyte consisting of 4.5 M LiFSI in PYR_13_FSI with 1 wt% LiTFSI as an additive. The anode‐free cell, composed of Cu foil coated by silicon–polyacrylonitrile and LiNi_0.5_Mn_1.5_O_4_ cathode with an optimized liquid electrolyte, showed a capacity retention of 80% after 120 cycles within 3.0–4.85 V cycling range at 22°C (Figure 6b) [148].

Pathirana et al. incorporated phosphonium bis(fluorosulfonyl)imide (P1222‐FSI) super‐concentrated ionic liquid electrolyte (LiFSI, 3.2 mol.kg^−1^) into Cu/NMC622 (4.6 mAh cm^−2^) anode‐free cell, which achieved average CE of 99.4% with 53% capacity retention after 100 cycles (Figure 6c), probably due to a favorable crosstalk between the cathode and the anode leading to a better SEI composition [149].

Looking ahead, data‐driven screening approaches are expected to play an increasingly important role in identifying electrolytes with high CE for AFBs. Recent work by Ma et al. demonstrated the use of active learning to accelerate electrolyte‐solvent screening for AFBs, highlighting how iterative experiments guided by machine learning can efficiently navigate large formulation spaces and converge on high‐performance electrolytes [157]. Such approaches offer a promising way to complement mechanistic understanding and reduce the experimental efforts associated with traditional trial‐and‐error electrolyte development, especially given the sensitivity of anode‐free systems to electrolyte composition and operating conditions.

### 3.4 Effect of Cathodes on the Performance of AFBs

For the practical implementation of AFBs, the full cell must be considered. The cathode influences the performance of AFBs in several ways, which is discussed here in particular with reference to its effects on the anode. Since AFBs do not contain Li on the anode side, the cathode primarily determines the available Li inventory, i.e., the total amount of cyclable Li [158]. In commercial cells with low electrolyte volume, the contribution of the Li salt in the electrolyte is negligible, so the cathode is the Li source. Two main strategies are used to increase the Li inventory: over‐lithiation of the cathode active material (CAM) and the addition of Li‐containing compounds into the cathode. The latter, often referred to as cathode pre‐lithiation, involves incorporating simple Li‐transition metal (TM) oxides or Li salts into the cathode, which release Li during the initial charge (or beyond) within the desired potential range of the battery [159]. Examples of Li‐TM‐oxide compounds with high capacities that are stable in air include Li_6_CoO_4_, Li_2_NiO_2_, Li_5_FeO_4_, Li_2_CuO_2_, Li_2_S and Li_2_MoO_3_. However, many of them lead to detrimental side reactions with the liquid electrolyte, TM dissolution, or oxygen release upon de‐lithiation [160]. Alternatively, sacrificial salts such as LiN_3_, Li_2_C_4_O_4_, Li_2_C_2_O_4_, Li_2_C_3_O_5_, and Li_2_C_4_O_6_ prevent TM dissolution but tend to cause significant gas evolution during de‐composition, further prolonging and complicating the formation process [161].

In contrast, over‐lithiation of the CAM itself does not lead to detrimental gas release or residual/increased TM‐dissolution, but not all CAMs can be over‐lithiated without problems. The two main strategies for achieving this over‐lithiation are chemical or electrochemical lithiation of the CAM, which have been studied since the early 1990s by e.g. Tarascon et al. [162]. While this early research focused on the lithiation of Li_1+x_Mn_2_O_4_ spinel, successful application to industrially produced NMC has been demonstrated. Here, chemical lithiation of LiNi_0.5_Mn_0.3_Co_0.2_O_2_ (NMC532) using a 50% mole excess of a 0.1 M lithium naphthalide solution to obtain Li_1+x_NiMnCoO_2_ resulted in an extra 20–70 mAh g^−1^ of Li in the CAM [162]. With an observed nearly twofold increase in cycle life compared to lithiation of the CAM itself, this is a promising avenue for addressing Li inventory loss in AFBs, which can benefit from the already significant advances in this area.

The second key factor affecting the AFB performance is the operating voltage of the cathode materials. Oxidative and reductive electrolyte decomposition pathways at different voltages influence SEI formation on both the current collector and the Li metal, affecting Li electrodeposition and dissolution [163]. Zhang et al. found that the dehydrogenation of ethylene carbonate (EC) to vinylene carbonate (VC) begins at 3.8 V vs Li^+^/Li. The acidic species generated during this process decompose the SEI, increasing its porosity and decreasing its stability [164]. Kwon et al. demonstrated the influence of cathode operating voltage on battery performance by comparing the electrolyte and SEI composition of LPF/Cu cells and NMC811/Cu cells under different voltage conditions. While LFP/Cu cells operate at a lower charging voltage of 3.5 V, NMC811/Cu cells reach 4.2 V [79]. The higher voltage of NMC811 leads to different electrolyte decomposition products, as observed by solution NMR and SEI analysis on Li, and results in denser and more reversible Li deposition in LFP/Cu cells compared to NMC811/Cu cells [165].

The third factor is that the composition of cathode materials influences the composition of the SEI through crosstalk. Rinkel et al. identified decomposition products and uncovered two distinct routes for electrolyte decomposition when they are in contact with the NMC811 cathode, each with a different onset potential [114]. At low potentials (i.e., <80% SOC), EC is dehydrogenated to VC, which may be coupled to the reduction of transition metal ions at the anode surface, but without the release of gaseous decomposition products. Second, more destructive mechanisms occur when the material reaches 80% SOC and ^1^O_2_ is released from the lattice: ^1^O_2_ chemically oxidizes the electrolyte solvent (EC) to produce H_2_O, CO_2_ and CO. The water formed then hydrolyses the electrolyte solution and triggers a series of reactions that were previously unknown in LIBs: the oxidation of alcohols to their corresponding aldehydes and carboxylic acids, the hydration of aldehydes to acetals, and the formation of FEC from VC. The increased parasitic reaction at the negative electrode reduces the available Li inventory and may contribute to the observed capacity fading when the cell is cycled to higher voltages. The release of ^1^O_2_ drives the electrolyte decomposition reactions at the NMC electrode, a process that is inextricably linked to achieving high SOC and thus higher cell capacities. Understanding the mechanism by which oxygen is released and developing strategies to prevent this process are valuable for improving the lifetime of lithium‐ion batteries, especially those containing the more environmentally friendly Ni‐rich NMC positive electrode materials [79].

The crosstalk‐based degradation of anode‐free cells is extensively studied for various electrolytes (carbonate and ether) and battery casing (pouch and coin cells). All review studies showed TM dissolution from the cathode and stainless‐steel casing, as well as electrodeposition of these TMs on the anode, leading to increased corrosion, interface heterogeneity, dendritic lithium growth, and short circuit‐induced degradation [166].

The fourth factor influencing the design of the negative electrode in AFBs concerns the targeted cathode loading and the rate performance of the cell. The cathode capacity determines how much Li is plated onto the CC during charging and how thick Li metal layer is at 100% SoC. Since the Li plating is accompanied by a volume expansion of approximately 100%, this directly contributes to cell swelling and pressure distribution within the cell [167]. The external cell pressure must be carefully optimized to preserve uniform Li morphology and ensure long‐term cyclability, especially in large‐scale pouch cells [168, 169].

Typically, industrial cells are designed with a low‐loading cathode for high power performance or with thick, high‐loading cathodes for high energy content. This has a direct impact on both the absolute amount of plated Li (thickness of the Li metal anode) and the kinetics of the cell, i.e., the maximum C‐rates. In addition, the amount of plated Li and the corresponding rate can have a profound impact on the morphology of Li layer, with Li dendrites and dead Li being the most unfavorable mechanisms leading to rapid cell failure (see Section 6) [170]. The choice of suitable modification strategies for the anode therefore also depends on the desired cathode properties and the expected performance.

In summary, cathode specifications are of great importance and must be carefully evaluated and matched to the performance of the anode design to achieve low degradation during cycling and maximum performance of AFBs. In this regard, research on Li inventory in conventional LIBs can be of great benefit.

### 3.5 Benchmarking Anode‐Free Batteries in Full‐Cell Configurations

Comparative evaluation of performance metrics is essential throughout all stages of technology development, from concept to commercialization. Table 4 summarizes the reported performance metrics of lithium anode‐free full cells, enabling comparison of cathode materials, current collector designs, electrolyte chemistries, and cycling conditions that govern Coulombic efficiency and capacity retention. Although the comparison of numerical parameters should, in principle, facilitate straightforward analysis and down‐selection of electrode and electrolyte chemistries and battery architectures, meaningful comparison across the current state of the art remains challenging.

**TABLE 4:** Summary of cathode characteristics, lithium inventory strategies, cell design parameters, and full‐cell electrochemical performance for anode‐free battery systems reported in the literature.

Cathode	Cathode areal capacity (mAh/cm^2^)	Current collector	Current collector modification	Electrolyte	Initial plating capacity	Lithium reservoir	C—rate (charge/discharge)	Coulombic efficiency	Capacity retention	Reference	Comment
NMC532	3.0	Cu	N/A	1 M LiFSI in FDMB	100%DOD	NO	0.2C/0.3C	—	80% after 100 cycles	[171]	Pouch cell, pressure of 250 kPa
LFP	1.6	Cu	Immersed in HCl (1 M) for 10 min	4 M LiFSI in DME	1.71 mAh/cm^2^	NO	0.2 mAcm^−2^/0.2mAcm^−2^	>99% after 50 cycles	60% after 50 cycles	[172]	
LFP	1.6	Cu	Immersed in HCL (1 M) for 10 min	1 MLiPF_6_ in EC/DMC (1/2 V/V)	1.71 mAh/cm^2^	NO	0.2 mAcm^−2^/0.2mAcm^−2^	—	First discharge only recovers 25% of total plated Li	[172]	
NMC111		Cu	N/A	1 MLiPF_6_ in EC/DMC (3/7 V/V)	—	NO	0.1C (0.14 mAcm^−2^)/0.1C	—	First discharge only recovers 23% of total plated Li	[173]	
NMC532	2.4	Cu	N/A	0.6 M LiDFOB+0.6 M LiBF_4_ in FEC/DEC (12 v/v)	80% DOD	NO	0.2C/0.5C	—	80% after 90 cycles	[146]	Pouch cell, pressure of 75 kPa
LFP	1.66	Cu	N/A	1 M LiPF_6_ + 0.5 M in FEC/DME blended with PVDF‐co‐HFP		NO	0.3 mA cm^−2^/ 0.3 mA cm^−2^	Average 99.7% after 100 cycles	56.1% after 100 cycles	[174]	Gel polymer electrolyte
LFP	1.8	Sn@Cu	Sn was plated by substitution reaction	1 M LiTFSI in 1:1 (v/V) DOL/DME with 1 wt% LiNO_3_	—	10 mAh cm^−2^ pre‐plated Li, N/P ratio: 5.6	0.5C/0.5C	Nearly 100% over 300 cycles	88.5% capacity retention after 300 cycles	[175]	Surface modification type: alloy
LFP	2.07	Zn‐N‐CNF (carbon nanofiber)	Current collector was repaired by electrospinning and high temperature carbonization	1 M LiTFSI in DME	>98% DOD	NO	0.5C/0.5C		91% after 120 cycles	[176]	
LFP	1.6	a‐RF@3D CM	pyrolysis of resorcinol formaldehyde on 3D engineered copper mesh	1 M LiTFSI in 1:1 (v/v) DOL/DME with 2 wt% LiNO3		cycled 2 times at 0.1C as formation cycle	0.2C/0.3C	Average CE >99.5% after 100 cycles	60.66% capacity retention after 100 cycles	[130]	
LFP	1.6	HPC‐1	HCl‐doped polyaniline modification layer through chemical polymerization	1 M LiTFSI in 1:1 (v/v) DOL/DME with 2 wt% LiNO_3_		NO	0.2C/0.3C	Initial CE of 63.81%	63.81% capacity retention after 100 cycles	[177]	Surface modification type: polymer
NMC333	11.57 mg/cm^2^	AOP@Cu	Milled Al_2_O_3_/polyacrylonitrile (PAN) composite layer (AOP) coated on Cu	1 M LiPF_6_ 1:1 EC:DEC (v/v) with 0.005 M KNO_3_		NO	0.2 mA cm^−2^/ 0.2 mA cm^−2^		30% after 82 cycles	[178]	Surface modification type: metal oxides
NCA	3.24	Cu	N/A	0.95 M LiPF_6_ + 0.05 M LiBOB in EMC:DMC:FEC:PC 3:3:3:1 (v/v) with 1% In_2_O_3_ nanoparticles		NO	0.1C/0.1C	Average CE 99.6% from 2 to 46 cycles	70% after 46 cycles	[179]	Surface modification type: metal oxides

A major limitation identified in this comparison is the lack of consistency in reporting of Coulombic efficiency. There is no consensus on the calculation methodology or the appropriate cycle number for reporting, whether during formation, after extended cycling, or at end of life. Furthermore, Coulombic efficiency is often conflated with capacity retention, and inconsistencies in cathode active mass loading, areal capacity, and depth of charge across studies making quantitative comparison impossible. As a result, the field is largely limited to qualitative, case‐by‐case comparison of reported studies.

While Table 4 may suggest superior performance for LFP‐based AFBs with ether‐based electrolytes and modified current collectors, this conclusion may be misleading as it does not take into account for fundamental limitations related to cathode energy density and electrolyte oxidative stability.

## 4 Anode‐Free Solid‐State Batteries (AFSSBs)

### 4.1 Overview of Solid Electrolytes and Interfacial Constraints in AFSSBs

Anode‐free batteries based on liquid electrolytes continue to demonstrate improved performance, but key challenges remain, including limited capacity retention, lithium corrosion, and safety concerns related to the high reactivity and flammability of liquid electrolytes [180, 181, 182]. In this context, solid electrolytes (SEs) have emerged as promising alternatives for the development of anode‐free solid‐state batteries (AFSSBs) [183, 184]. By replacing volatile liquid electrolytes with non‐flammable, ion‐conducting solids, AFSSBs offer a fundamentally different approach that could mitigate several intrinsic limitations of liquid‐based AFBs. However, unlike liquid systems, SEs impose rigid electrochemical and mechanical constraints at the electrolyte/lithium interface, making interfacial phenomena a central determinant of performance in anode‐free configurations.

Several classes of SEs for solid‐state batteries are currently being intensively investigated, with each class offering distinct advantages and limitations that are particularly relevant in the absence of excess lithium. From an anode‐free perspective, these materials can best be distinguished not only by their bulk ionic conductivity, but also by their electrochemical stability toward lithium metal, their mechanical compliance, and their ability to form and retain low‐resistance solid–solid interfaces during repeated lithium plating and stripping [185].

Sulfide‐based SEs such as Li_10_GeP_2_S_12_ (LGPS) and argyrodite‐type Li_6_PS_5_X (X = Cl, Br, I) have initially revitalized the field of solid‐state batteries due to their high room‐temperature ionic conductivity, which can exceed 10^−^^2^ S cm^−^^1^ and can compete with that of liquid electrolytes [186]. In addition to their favorable transport properties, sulfides exhibit good mechanical formability, enabling cold processing without high‐temperature sintering and facilitating intimate initial contact with the electrode materials. These properties are attractive for AFSSBs, where homogeneous lithium nucleation and low interfacial resistance are essential. However, sulfide SEs suffer from limited chemical and electrochemical stability, as they readily react with lithium metal and ambient moisture, releasing toxic H_2_S gas. Moreover, their relatively narrow electrochemical stability window and pronounced chemo‐mechanical degradation often necessitate the application of external stack pressure during operation to maintain interfacial integrity, which complicates their practical implementation in anode‐free designs [187, 188, 189].

Oxide‐based SEs form another important class of potential SEs, with garnet‐type Li_7_La_3_Zr_2_O_12_ (LLZO), NaSICON‐type Li_1_._5_Al_0_._5_Ti_1_._5_(PO_4_)_3_ (LATP), and perovskite‐type Li_3__x_La_2_/_3__−__x_TiO_3_ (LLTO) being the most extensively studied materials. These SEs exhibit moderate room‐temperature ionic conductivities, typically in the range of 10^−^^4^ to 2 × 10^−^^3^ S cm^−^^1^, combined with excellent thermal stability, non‐flammability, and wide electrochemical stability windows that are compatible with high‐voltage cathodes. From an anode‐free perspective, oxides such as LLZO are particularly attractive due to their superior chemical stability towards lithium metal [190, 191]. At the same time, their high stiffness and brittle nature pose significant challenges for interface formation and contact retention. The need for high‐temperature sintering (>1000°C) during processing further complicates the control over surface chemistry and often leads to insulating surface layers and poor wetting, which are particularly disadvantageous in AFSSBs, where lithium must nucleate directly at the SE interface [192, 193].

More recently, halide‐based SEs have emerged as promising candidates that combine several advantageous properties of sulfides and oxides [194]. Materials such as Li_3_InCl_6_ and Li_3_YCl_6_ exhibit relatively high ionic conductivity, high oxidation stability up to about 5 V vs. Li^+^/Li, and improved tolerance to ambient air compared to sulfide electrolytes. In addition, halides can typically be processed by cold compaction, avoiding high‐temperature sintering. These properties make halide SEs attractive for AFSSBs from both interfacial and manufacturing perspectives. However, the majority of halide electrolytes are thermodynamically unstable at low potentials and are reduced by lithium metal, leading to the formation of poorly performing interphases [195, 196]. Furthermore, the dependence on rare or expensive elements in some compositions raises concerns about cost and scalability.

Polymer‐based SEs, including systems based on poly(ethylene oxide), poly(methyl methacrylate), and poly(vinylidene fluoride), represent a fundamentally different class of solid electrolytes that has long been researched for solid‐state batteries [197]. Among these, poly(ethylene oxide)‐based systems have achieved commercial introduction, notably by Bolloré in 2011 [198]. Polymers offer inherent mechanical flexibility, ease of processing, and excellent interfacial contact with electrodes and current collectors, which is advantageous for adapting to lithium volume changes in anode‐free cells. However, their practical application is limited by low ionic conductivity at room temperature (typically 10^−^^8^–10^−^^6^ S cm^−^^1^) and limited oxidation stability, often necessitates elevated operating temperatures (60–80°C) to achieve acceptable performance, these operating temperatures are likely to exacerbate electrolyte oxidation. Current research efforts are therefore focused on improving the conductivity and stability of polymers through molecular design strategies that reduce crystallinity, such as the incorporation of flexible side chains, and through hybrid polymer–inorganic composites that promote ion transport via multiple mechanisms [198].

**FIGURE 7:**
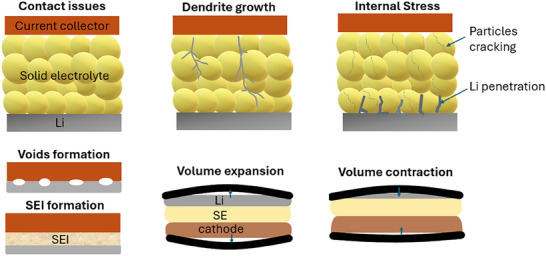
Schematic illustration of the key interfacial and chemo‐mechanical constraints governing anode‐free solid‐state batteries.

Overall, while each class of solid electrolyte offers distinct advantages for anode‐free solid‐state batteries, none simultaneously meets all the interfacial and chemo‐mechanical requirements necessary for lithium‐free operation (Figure 7).

Without excess lithium, stable cycling depends on maintaining close contact at the interface between the solid electrolyte and the CC during lithium plating and stripping, regulating lithium nucleation and current distribution, and preventing both the formation of interface voids or delamination and penetration of lithium through the solid electrolyte. At the same time, reactions between lithium metal and many SEs lead to the formation of high‐impedance interphases, which impair cell performance and cycling stability. In this context, sulfides and polymers favor initial contact and mechanical compliance but suffer from chemical instability with formation of interphases with unfavorable properties; oxides such as LLZO offer superior electrochemical stability but are limited by rigid interfaces resulting in loss of contact during cycling; and halides could occupy an intermediate position that is promising but issues with reduction stability still remain unresolved. These interrelated constraints underscore that electrolyte selection alone is insufficient, leading to interface‐centered design strategies discussed in the following section.

### 4.2 Interface‐Centered Design Strategies for AFSSBs

In anode‐free solid‐state batteries, lithium is not plated from a pre‐existing Li reservoir but must nucleate and grow in situ at a buried solid–solid interface. Consequently, the dominant failure modes (poor nucleation, void formation upon stripping, inhomogeneous current distribution and Li penetration) are primarily interfacial and chemo‐mechanical in nature. Rather than being dictated by bulk electrolyte conductivity alone, the performance of AFSSB is therefore governed by how the interface regulates (i) the nucleation barrier, (ii) the spatial distribution of current and Li flux, and (iii) the ability to maintain interfacial contact during repeated plating/stripping.

**FIGURE 8:**
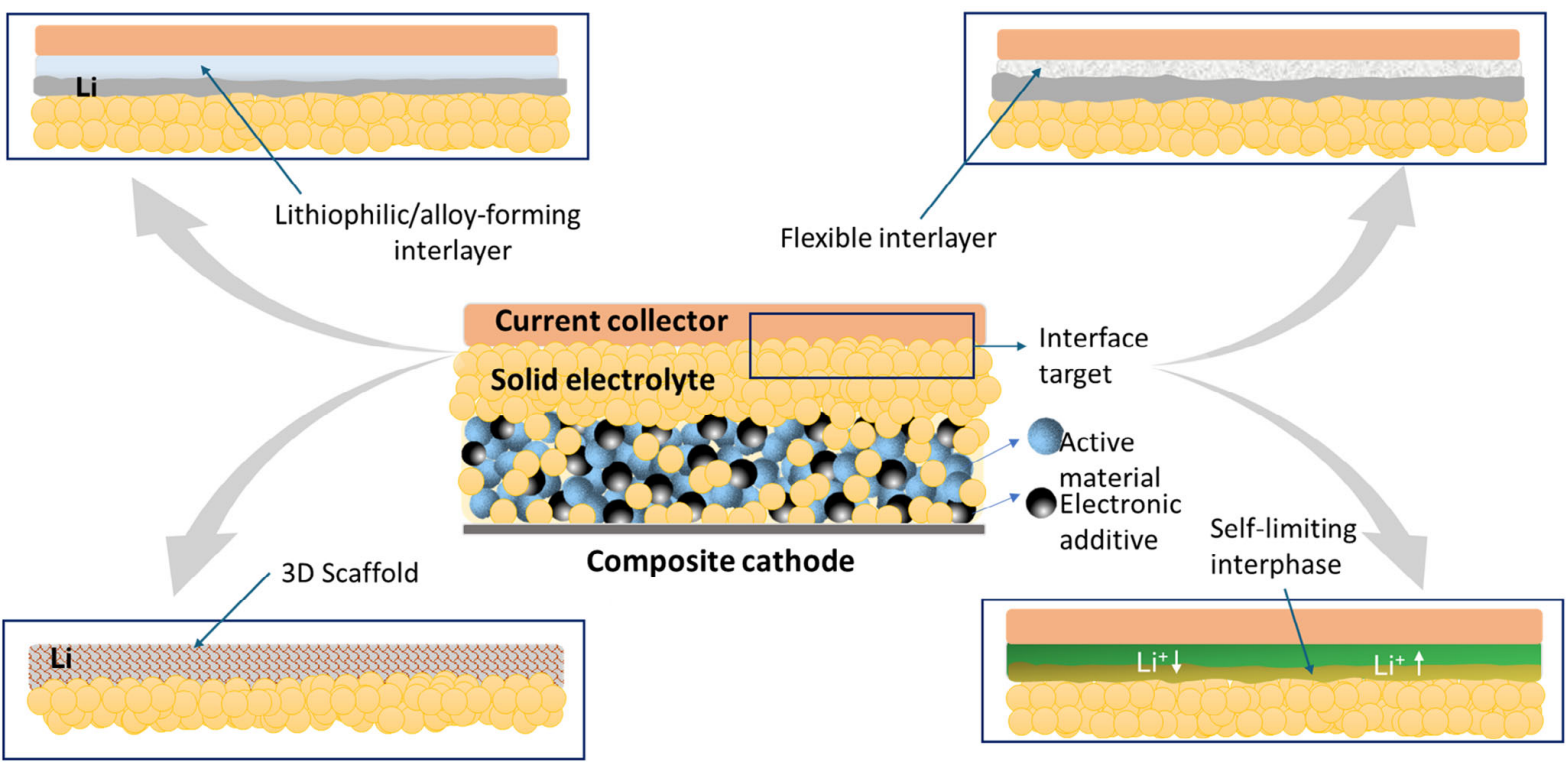
Schematic illustration of interfacial engineering strategies for anode‐free solid‐state batteries. (a) regulation of lithium nucleation and current distribution using lithiophilic and alloy‐forming interlayers; (b) preservation of interfacial contact through mechanically adaptative and compliant buffers to accommodate volume changes; (c) architectural control of lithium growth using thin‐film and three‐dimensional interface designs to distribute current and confine deposition; and (d) control of interphase chemistry to form stable, ionically conductive, and electronically insulating layers.

In Figure 8 we have organized the reported approaches according to key interfacial design strategies.

#### 4.2.1 Regulating Li Nucleation and Current Distribution Using Lithiophilic/Alloy‐forming Interlayers

A recurring mechanistic motif in AFSSBs is that, for stable cycling, the Li nucleation barrier must be lowered and localized current hotspot must be avoided. Similar to liquid‐based configurations, this is typically achieved by introducing lithiophilic and/or alloy‐forming interlayers that provide energetically favorable nucleation sites and redistribute current at the interface. Importantly, with solid electrolytes, Li nucleation often occurs at the SE surface, and lithiophilic layers are therefore typically applied to the surface of the SE separator to promote homogeneous deposition, rather than to the CC surface.

A representative example is the use of Ag–C composite buffer layers (BLs) between the CC and the SE [199]. Wang et al. demonstrated that Ag–C interlayers enable stable lithium plating in anode‐free sulfide‐based solid‐state batteries by exploiting the reversible alloying of Ag with Li, which forms a homogeneous solid solution beyond AgLi_2_._32_ and maintains a positive potential even at high degrees of lithiation [200]. Functionally, Ag–C interlayers bias lithium deposition toward the BL|CC interface when the interfacial resistance at BL|CC is lower than at BL|SE, while the substantial Ag–Li volume expansion during lithiation assists in maintaining interfacial conformity [201]. Lee et al. showed how CCs composed of Ag nanoparticles in a composite with carbon black lead to the improvement on CE and cycling performance of 0.6 Ah solid‐state pouch cells with sulfide electrolyte and high loading cathodes [136]. Similarly, solid AFB with oxide electrolytes benefitted of using thin Ag layers on the Cu CC [135]. Other metals proved less effective due to higher nucleation overpotentials, premature termination of lithiation, or insufficient volume expansion to maintain favorable growth geometry.

This strategy has been demonstrated in sulfide and oxide systems. For example, argyrodite Li_6_PS_5_Cl(LPSCl) exhibited high cyclability (>1000 cycles) and an average Coulombic efficiency (CE) of 99.8% when combined with a thin Ag–C interlayer, with partial Ag alloying at the onset of charging promoting dendrite‐free Li plating. Xie et al. subsequently elucidated the microscopic mechanism and showed that Ag clusters continuously lithiate and delithiate during cycling, maintaining interfacial lithiophilicity and suppressing dead‐Li formation [201]. Similar concepts were extended by Kim et al. to garnet electrolytes, demonstrating improved cycling stability in Ta‐doped LLZO using Ag–C interlayers [202].

Beyond Ag, alloying/conversion layers can similarly create nucleation seeds and ionically favorable interphases. Wang et al. demonstrated that pretreatment of the CC can enable nucleation control without creating thick inactive layers. Thus, in a AFSSB with Li_6_PS_5_Cl electrolyte, exposure of Cu CC to Te vapor followed by in situ lithiation resulted in a formation of lithiophilic Li_2_Te layer, which enabled uniform lithium deposition without voids or dendrites, as confirmed by cryogenic FIB analysis. Full cells with NMC811 cathodes achieved an initial Coulombic efficiency of 83% and a cycling efficiency of 99% at 0.2C, highlighting the critical role of CC surface chemistry in AFSSBs [68].

**FIGURE 9:**
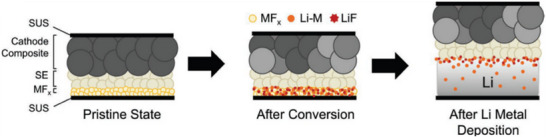
Schematic representation of the conversion reaction of metal fluoride (MF_x_), followed by Li plating in the bulk metallic form. Reproduced with permission [203]. Copyright 2022, Wiley‐VCH.

A broader chemical space for nucleation control was explored by Lee et al., who investigated the conversion–alloying reactions of metal fluorides (MF_x_, M = Ag, Sn(II), In, Zn, Bi, Al, Sn(IV), Mg, Ca, Cs). They showed that the conversion (xLi + MF_x_ → M + xLiF) generates metallic nanodomains and LiF followed by alloying (Li + M → Li–M), which enables reversible lithium plating. Among the investigated systems, AgF was identified as the most effective, as the resulting metallic nanodomains reduced the nucleation potential, while LiF mitigated dendrite growth and suppressed Ag agglomeration, enabling uniform plating at high areal capacities (Figure 9) [203].

**FIGURE 10:**
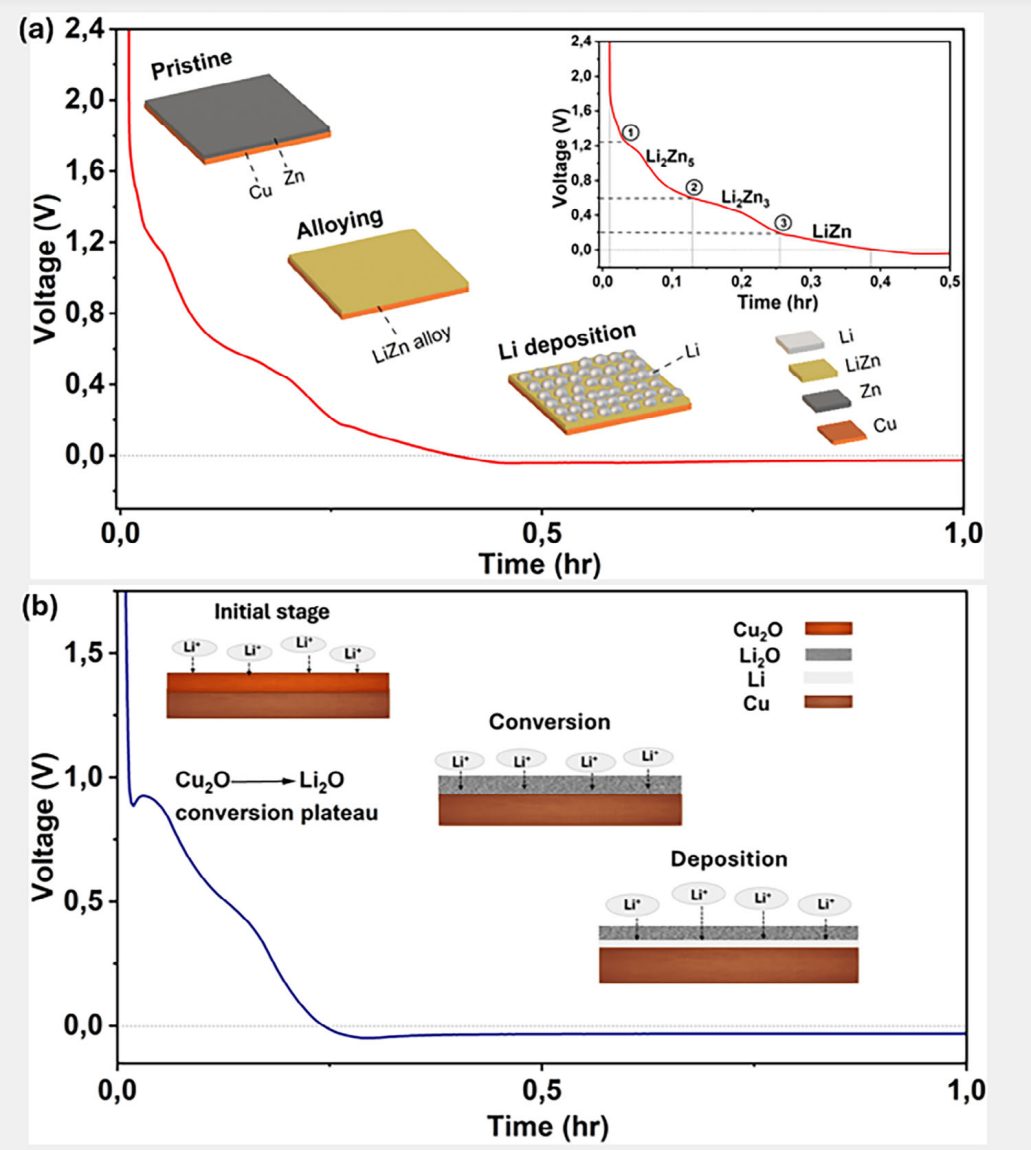
Li plating at 0.05 mA/cm^2^ of (a) 600 nm Cu|50 nm Zn|LLZO|Li and, (b) 600 nm Cu|50 nm Cu_2_O|LLZO|Li half‐cells, inset figure present the conceptual schematic of the structural and compositional evolution during Li deposition at the Cu/Cu_2_O/LLZO interface. Reproduced with permission [205]. Copyright 2025, Wiley‐VCH.

In oxide‐based AFSSBs, where lithiophobicity and contact issues are pronounced, ultrathin alloying layers have been shown to reduce the nucleation barrier while minimizing energy losses. Fallarino et al. demonstrated that sputtered Ag interlayers of up to 100 nm on Nb‐doped LLZO enabled a pressure‐free cycling through reversible Ag–Li alloying, although the average Coulombic efficiency remained limited (∼88%), suggesting that nucleation control alone is not sufficient to ensure high reversibility [204]. More recently, Rafique et al. investigated ultrathin (50 nm) Zn and Cu_2_O interlayers sputtered onto LLZO beneath a 600 nm Cu current collector. They showed that Zn forms in situ Li–Zn alloys that lower the nucleation barrier and accelerate plating kinetics, promoting columnar growth, while Cu_2_O interlayer reacts with the formation of Li_2_O as one of the reaction products, creating a lithiophilic, ion‐conducting interface that promotes more homogeneous deposition and improved resistance to surface protrusions and fractures (Figure 10) [205]. Related studies on sputtered interlayer by Ko et al. further demonstrated that alloying interlayers (Ag, Au, Zn) can act either as nucleation seeds or as buffers that favor the growth of lithium toward the current collector, with Ag providing the most robust interfacial stability [206].

#### 4.2.2 Maintaining Interfacial Contact During Li Stripping Through Compliant Layers and “Mechanically Adaptive” Buffers

Even if Li can be deposited homogeneously in the first plating step, AFSSBs often fail during Li stripping because solid–solid interfaces do not self‐heal. The dissolution of Li can create voids, partial delamination, and local resistance buildup, leading to formation of current hot spots (current focusing) and ultimately Li penetration/shorting. A second important strategy therefore aims to maintain dynamic contact by incorporating mechanically compliant and ductile components that accommodate volume changes and keep intimate contact with the SE during both plating and stripping.

Oh et al. addressed the persistent problem of volume change by combining Ag–C nucleation control with an elastic polymer binder (Spandex). They demonstrated that the synergistic effect of soft and hard segments, which enable hydrogen bonding and elastic network adaptation, significantly improved Coulombic efficiency and long‐term cycling stability compared to PVdF‐based analogues (100 cycles, cumulative efficiency of 86.0% vs. 59.2%). This work clearly demonstrates that nucleation control strategies benefit from coupling with mechanical compliance to mitigate void formation caused by stripping [207].

**FIGURE 11:**
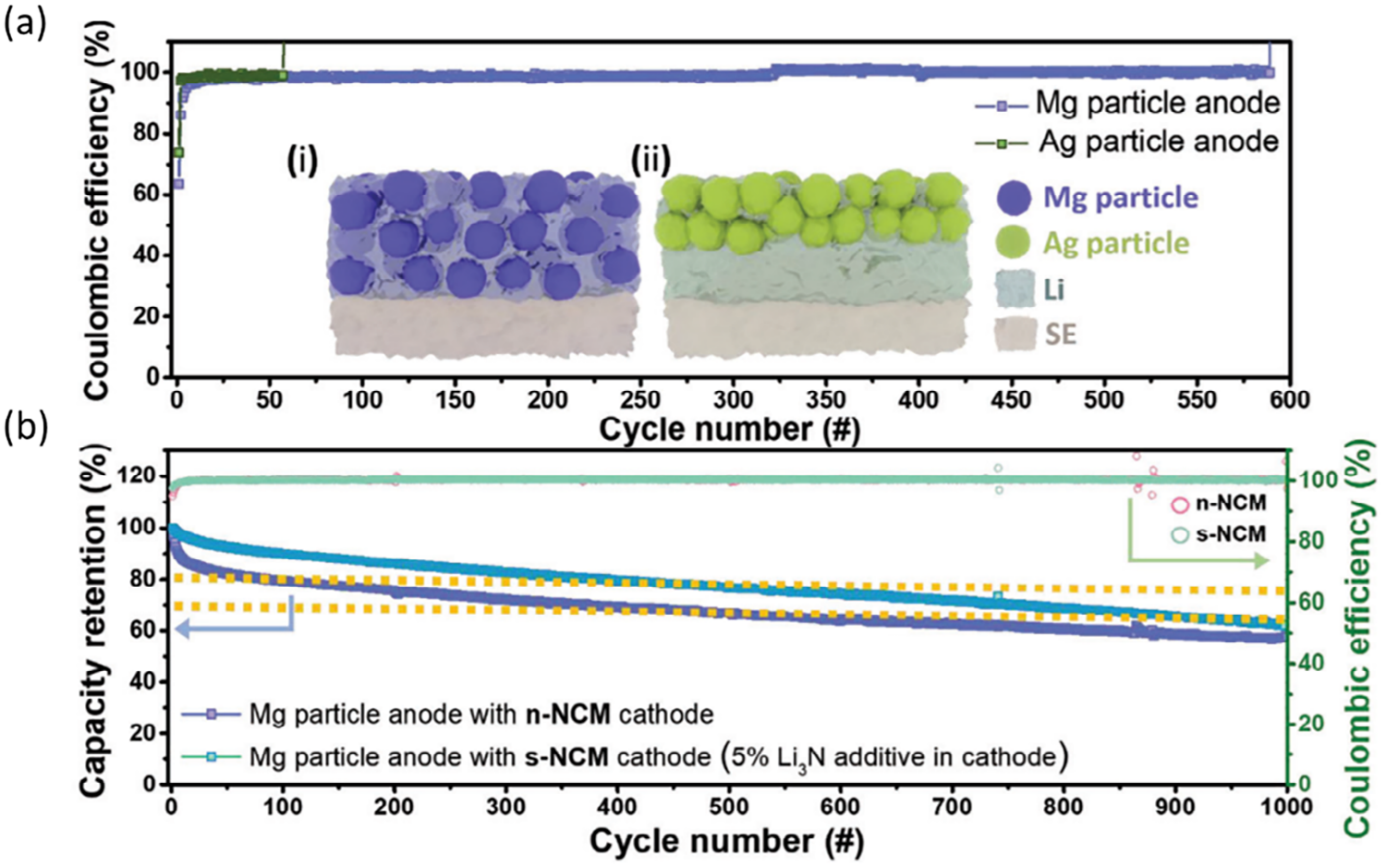
(a) Coulombic efficiency variation during the repeated Li plating and striping on Mg and Ag particles in half cells. The deposition was at 0.5 mA cm^−2^ to a capacity of 2 mAh cm^−2^ at 30°C and 30 MPa. (b) Cycling performance of full‐cell SSB with NCM811 cathode and Mg particle anode with (s‐NCM) and without (n‐NCM) 5 wt.% Li_3_N as cathode sacrificial agents. Reproduced with permission [209]. Copyright 2024, Wiley‐VCH.

Similarly, Oh et al. demonstrated that ductile buffer layers enable stable cycling at reduced stack pressure. In an anode‐free sulfide system, a thin Ti_3_C_2_T_x_ MXene layer under a Mg film maintained close interfacial contact even at low pressure. The ductility of the MXene buffer supported stable lithium plating and stripping, resulting in improved lifespan and Coulombic efficiency at room temperature and 2 MPa compared to pressure‐intensive baselines [208]. The performance of AFSSBs with a “breathing” Mg anode has been reported by D. Jun et al. (Figure 11) [209]. These results are consistent with more general observations that external pressure can suppress void formation, but practical AFSSBs require self‐adaptive interfaces that do not rely on sustained high mechanical loads.

#### 4.2.3 Engineering Interface Architecture to Control Li Formation and Li Growth (From 2D planar to 3D host structures)

A third strategy addresses the fact that in solid‐state systems, the location and geometry of Li growth are dictated by the availability of electrons, the length of triple‐phase boundary (TPB), and the local heterogeneity of the interface. Instead of relying solely on chemical interlayers, architectural control can (i) distribute current over a larger effective area, (ii) confine Li growth to predetermined volumes, and (iii) reduce stresses by providing space for Li deposition without forcing large planar displacement [210].

In oxide systems with rigid interfaces, Rafique et al. have shown that the deposition of ultrathin current collectors is an alternative to high temperature joining and high stack pressure. By sputtering nanometric Cu collectors directly onto LLZO at room temperature, they enabled the operation of AFSSBs for hundreds of hours with Cu layers only 50 nm thick [211]. Using microscopy and in situ impedance spectroscopy, they showed that minimizing interfacial roughness and improving electronic uniformity via conformal CC deposition can provide a scalable path for oxide‐based AFSSBs.

The morphology of the interlayer itself can also act as an architectural variable. Haslam et al. compared flat and clustered Au seed layers on Cu in garnet‐based cells and showed that clustered Au seeds promote reversible alloying with lithium, significantly lowering the nucleation overpotentials and enabling stable plating of several micrometers of lithium with a Coulombic efficiency of about 97% at 60°C and 2.5 MPa. In contrast, flat Au layers failed rapidly, underscoring the importance of interlayer topology over chemistry alone [191].

A more structural approach was demonstrated by Yang et al., who engineered a tri‐layer porous/dense/porous garnet framework [212]. By coating one side with Cu and the opposite porous side with conformal ZnO, followed by melt infiltration of lithium, they enabled lithium to be shuttled and plated into the initially empty side starting at the Cu–LLZO–Li triple‐phase boundary. This design enabled uniform, dendrite‐free lithium growth within the 3D framework at current densities of 0.5 mA cm^−^^2^. In a follow‐up study, Yang et al. extended this concept by introducing a mixed ionic–electronic conducting garnet, Li_6_._4_Ga_0_._2_Pr_3_Zr_1_._8_Ce_0_._2_O_12_, combined with ZnO deposited on both sides by ALD, enabling conformal lithium plating throughout the porous network [213]. This architecture delivered exceptionally high critical current densities and stable long‐term cycling while simultaneously accommodating lithium volume changes within the host structure.

#### 4.2.4 Controlling Interphase Chemistry and Resistivity to Avoid Continuous Growth of Electronically Conductive Interphases

Since many SEs (except LLZO and LiPON) are thermodynamically unstable with Li, the formation of interphases is unavoidable in most AFSSBs. The critical design question is therefore not whether an interphase forms, but whether it is self‐limiting, ionically conductive, and electronically insulating. If the interphase is electronically conductive, parasitic reactions can proceed continuously, consuming plated Li and preventing stabilization. If it is highly resistive, it can cause large polarization and lead to inhomogeneous deposition.

Operando interphase analysis using virtual electrode plating (formation of a “virtual electrode” following exposure of a grounded and Li‐backed SE surface to an electron beam) XPS shows that Li_10_GeP_2_S_12_ (LGPS) forms a rapidly growing, mixed ionic–electronic SEI that impedes the stable formation of Li metal during plating, while argyrodite Li_6_PS_5_Cl (LPSCl) forms a more passivating interphase with reduced electronic conductivity that stabilizes the interface and allows Li plating with less dynamic SEI evolution. These differences in the chemistry and electronic properties of the interphase are revealed by distinct decomposition products identified using in situ/operando XPS techniques [214].

This framework also explains why halides and sulfides often require coatings (e.g., LiNbO_3_, Li_3_PO_4_) and why surface contamination layers (e.g., Li_2_CO_3_ on LLZO) are so detrimental: in both cases, interfacial resistance is created and nucleation behavior is altered. This further emphasizes that interlayer strategies based solely on alloying must be combined with interphase control, otherwise the interfacial resistance will remain high and reversibility will be limited.

#### 4.2.5 Cross‐Cutting Considerations: Pressure, Interlayer Thickness, and Measuring the Interface Under Realistic Cycling States

Two practical limitations repeatedly arise with these strategies. First, many demonstrations rely on high stack pressures (often several MPa to tens of MPa) to maintain contact and suppress void formation. While pressure can improve cycling, it complicates packaging and is unlikely to be sustainable in commercial formats, motivating a shift toward mechanically compliant interfaces and architectures that maintain conformity at lower pressure [215, 216, 217].

Second, the thickness of the interlayer directly reduces energy density as has been shown in our calculations in Chapter 2. Therefore, ultrathin coatings (tens of nm) and conformal deposition routes (e.g., sputtering, ALD) are attractive, but their mechanistic effects may be sensitive to the morphology and location of the final Li deposition (SE/interlayer vs. CC/interlayer). Studies comparing Cu, Au, and pre‐lithiated Cu collectors in LLZO systems confirm that a pre‐existing Li reservoir or alloy layer can suppress Li penetration and that interface roughness and contact heterogeneity are important factors in whisker/dendrite formation at high currents.

Finally, despite many reported improvements, interfacial resistances in AFSSBs often remain very high (several hundred to >1000 Ω in some reported cases). In contrast, anode‐free cells with highly concentrated liquid electrolytes and engineered Cu coatings can achieve values well below 100 Ω, and conventional anode SEI resistances in LIBs often start in the double‐digit to low triple‐digit ohm range, depending on ageing (for a typical 1–2 cm^2^ geometric area of coin or Swagelok cells). Although the protocols and cell formats differ substantially, this comparison highlights the gap that needs to be closed in order to realize AFSSBs with competitive power capability and lifetime. Since the interfacial resistance in AFSSBs is expected to depend heavily on the state of charge (degree of plating/stripping) and the changing contact area, future work should focus on operando impedance measurements in conjunction with chemical/microstructural analysis during cycling to directly link the interphase evolution, morphology, and electrochemical reversibility [218].

### 4.3 Thin Film ASSBs

**FIGURE 12:**
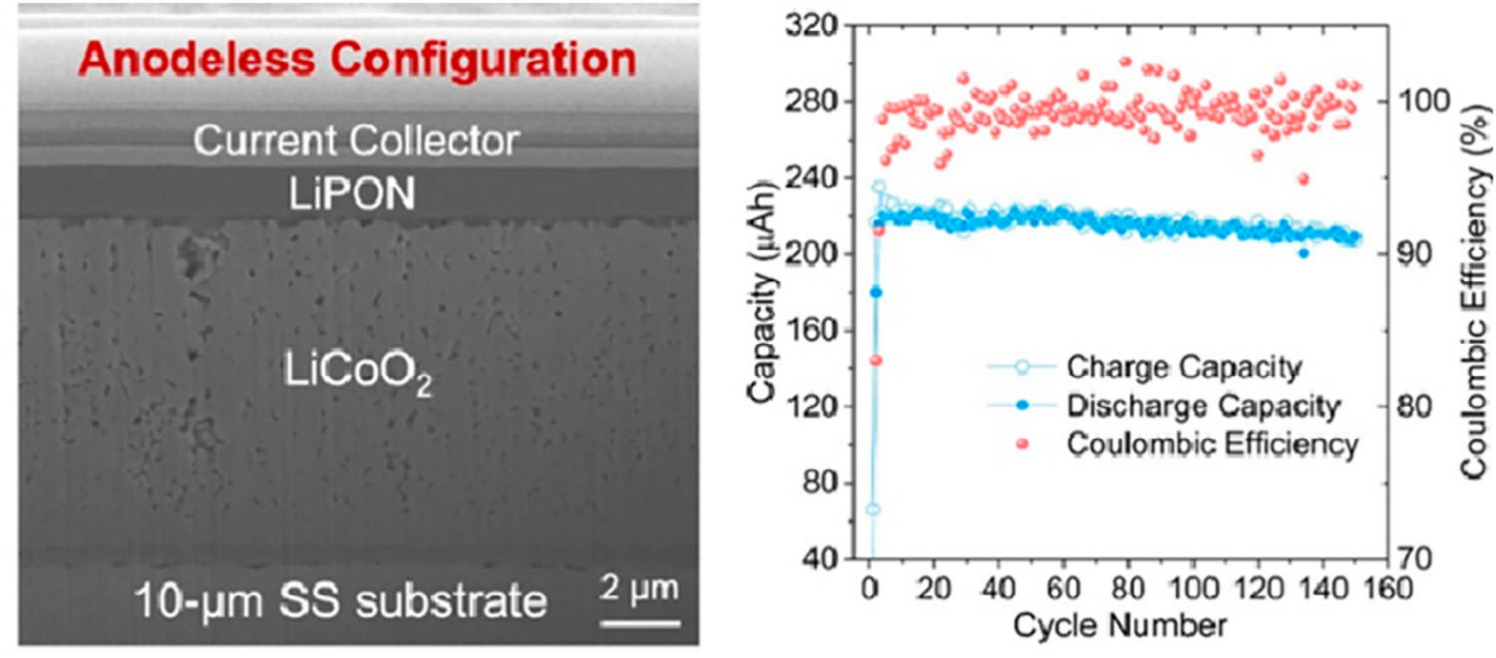
Cross‐section SEM view of the stacking of components of the thin‐film (left) and cycling performance of an anode‐free full cell (right). Reproduced with permission [225]. Copyright 2023, ACS.

The concept of the anode‐free thin‐film battery was first introduced in the 1990s by Bates et al., who used physical vapour deposition (PVD) to fabricate micro‐batteries with LiCoO_2_, amorphous LiPON SE, and Cu as the current collector [219]. Compared to crystalline electrolytes, amorphous electrolytes offer a viable option for stabilizing Li metal deposition due to their isotropic ionic conduction, negligible electronic conductivity, and, importantly, the virtually complete absence of resistive grain boundaries, which can be related to the very small size of the grains (in the nm range) [220]. LiPON has a low ionic conductivity of about 10^−6^ S cm^−1^ at 25°C and is therefore limited to thin film batteries. However, LiPON has remarkable properties such as high oxidation stability of up to 5 V, making it compatible with high‐voltage cathodes, and forms a stable SEI with Li metal, ensuring stable lithium plating and stripping and high capacity retention over thousands of cycles [221, 222]. Westover et al. have confirmed that the resistance of LiPON to Li dendrites growth is due to its defect‐free, grain boundary‐free structure, highlighting the need for further research on amorphous electrolytes [223]. Furthermore, the nanoscale size of the electrolyte can mitigate problems associated with the solid‐solid interface (electrolyte‐electrode), e.g., reducing contact loss at the Li metal/electrolyte interface, thereby extending battery lifetime. Di. Cheng et al. demonstrated the successful use of an anode‐free configuration in a thin‐film battery using a LiCoO_2_ cathode and a thin stainless‐steel substrate on LiPON SE (Figure 12) [224, 225]. The cell with an areal capacity of 0.55–0.66 mAh cm^−2^ was cycled for 150 cycles and maintained a capacity retention of approximately 95%. Neudecker et al. reported on a thin‐film battery with an anode‐free design using amorphous LiPON electrolyte that operated for over 1000 cycles at 1 mA cm^−^^2^ [226].

A. Müller et al. investigated Au, Pt and C as seed layers placed between a Cu CC and a LiPON thin‐film SE. These layers can improve Li plating and stripping dynamics, with reversible plating demonstrated for currents up to 8 mA cm^−2^. Au and Pt seed layers alloyed with Li early in the plating process, enabling uniform plating on the CC. Among all seed layers, C enabled a significant reduction in the plating potential from 300 to 50 mV [227].

Thin‐film microbatteries offer a unique platform for investigating the role of interfaces and interphases in electrochemical performance, particularly due to the stability of the Li/LiPON interphase and the precision of PVD fabrication. These factors contribute to their superior performance compared to conventional AFSSBs. It is noteworthy that the first thin‐film battery based on amorphous LLZO already showed resistance to Li dendrite growth and increased cycle stability without any structural optimization [158].

However, the practical application of thin‐film AFSSBs is hampered by significant limitations, including significantly lower areal capacity (0.5–0.7 mAh cm^−^^2^) compared to conventional cells (3–5 mAh cm^−^^2^) and challenges related to the production process. The deposition of LiPON, which typically has a thickness of about 1 µm in thin‐film microbatteries, is inherently slow, and cathode processing follows a similarly thin and time‐consuming approach. Furthermore, high manufacturing costs due to expensive and slow deposition processes, as well as low areal capacity, limit their suitability for large‐scale applications such as electric vehicles. Despite these challenges, insights from thin‐film microbatteries provide valuable knowledge for improving the interface stability and performance in conventional battery technologies. By understanding and adapting these design principles, particularly in optimizing interface stability and deposition methods for the construction of intermediate layers, conventional batteries could benefit from improved efficiency, longevity, and safety.

## 5 Anode‐Free Sodium Batteries (AFSBs)

Research into sodium‐ion batteries as a sustainable and cost‐effective alternative to lithium‐ion batteries has gained momentum again since 2010 [228, 229, 230]. Following the discovery of fast Na‐ion conductivity in β‐alumina in 1967 [231], which led to the development and commercialization of high‐temperature cells (300–350°C) using molten metallic sodium as anode, β‐alumina as separator/electrolyte, and sulfur (Na‐S‐battery) or transition metal (e.g., Ni) chlorides (ZEBRA) as the cathode [232]. Shortly thereafter, in 1976, the discovery of the Na superionic conductor Na_1+3x_Zr_2_(Si_x_P_1−x_O_4_)_3_ (NASICON) by Goodenough et al. was published [233]. However, due to the rapid development of Li intercalation compounds, which led to the commercialization of the first Li‐ion battery by Sony in 1991 [234], work on sodium‐ion batteries was discontinued and the research community focused on improving Li‐ion batteries [230].

The uneven distribution of Li resources and production—which is largely concentrated in a small number of countries [235]—as well as increasing pressure on supply chains for other critical battery materials, such as battery‐grade graphite and refined transition metals, have driven research into Na‐ion batteries [236, 237]. Compared to Li (20 ppm), Na is not only more abundant in the upper Earth's crust (about 23,600 ppm, >2%), but also more evenly distributed. Na‐ion batteries do not rely on graphite, but on more readily available hard carbons, and the composition of the cathode material contains lower concentrations of cobalt and nickel, such as the P2‐type layered cathode Na_0.67_Ni_0.23_Fe_0.1_Mn_0.67_O_2_ instead of LiNi_0·8_Co_0·1_Mn_0·1_O_2_ (NMC811) [238, 239]. Therefore, there is the potential to diversify the battery supply chain by developing Na‐ion batteries. Despite the proximity of Li and Na in the periodic table, the physicochemical differences lead to distinct differences in their electrochemical properties [239, 240]. Since Na has a significantly higher molar mass than Li (23.0 g mol^−1^ vs. 6.94 g mol^−1^), the theoretical specific gravimetric capacity of Na (1166 mAh g^−1^) is lower than that of Li (3861 mAh g^−1^) [241]. The standard electrode potential is higher for Na (−2.71 V) than for Li (−3.04 V) [238], which reduces the energy density but allows the use of Al instead of Cu as the negative CC [242, 243].

The ionic radius of Na (102 pm) is larger than that of Li (76 pm), resulting in a significantly higher molar volume (∼24 vs. ∼14 cm^3^ mol^−1^). This means that a Na metal layer deposition on the anode current collector of a certain capacity (mAh) will require more space than a Li metal layer of the same capacity [244]. While the density Li metal is 0.53 g cm^−3^, that of sodium metal is almost twice as high (0.97 g cm^−3^), resulting in an inherently lower specific energy for sodium AFBs.

In their metallic state, both lithium and sodium crystallize at room temperature as soft body‐centered‐cubic metals [245]. However, the larger size of sodium results in lower cohesive energy (Li: 36.5 kcal mol^−1 ^vs. Na: 26 kcal mol^−1^) [246] and thus in a lower melting point (97.8°C) compared to lithium (180.5°C) and a higher plasticity [247]. This drastic difference could be reflected in the nucleation and growth behavior during plating, especially at elevated stack pressures and temperatures. The yield strength—the maximum stress a material can withstand before it undergoes permanent deformation—is different for both alkali metals, with polycrystalline lithium ranging between 0.73 and 0.81 MPa and polycrystalline sodium ranging between 0.19 and 0.28 MPa [248, 249]. Although these fundamental properties were measured on polycrystalline metal foils, this difference in plastic flow behavior is expected to be particularly relevant for AFSBs with SEs, which are described in section 5.3.

The reversible electrodeposition of sodium at room temperature from various salt solutions prepared with propylene carbonate on a Pt substrate was already described in 1975 by J. Jorné and C.W. Tobias [250]. It was shown that relatively thick deposits (0.5 mm) could be formed at high current densities of 10 mA cm^−2^. Unlike Li, Na does not alloy with the Pt substrate. Experiments were conducted in both the anodic and cathodic directions to demonstrate a high degree of reversibility of the electrochemical deposition and dissolution process of Na at room temperature. However, the morphology of the deposits and long‐term stability were not the focus of this work [250].

The obstacles to stable plating and stripping of sodium metal layers in liquid electrolytes are similar to those described in Section 3 for lithium‐based systems, namely non‐uniform sodium deposition and SEI formation on the negative CC either during the initial cycle or as a result of repeated cycling, yielding inactive sodium that no longer has electrical contact with the current collector (i.e., ‘dead sodium’). Over the course of several tens to hundreds of charge‐discharge cycles, this leads to the formation of dendritic structures that can cause short circuits, reducing the Coulombic efficiency and cycle life and raising serious safety concerns [251].

While Na electrodeposition is frequently analysed by analogy to Li systems, new findings point to important mechanistic differences. In Li systems, SEI losses are the primary failure modes for high CE systems [75], while Na systems tend to be limited by the accumulation of dead sodium and associated mass transport limitations, which are exacerbated by the comparatively good solubility of SEI [252, 253, 254]. To reduce the loss of active Na during cell cycling, the modification of current collectors and the optimization of the electrolyte have been tested as the most important mitigation strategies, as discussed below [255, 256, 257].

### 5.1 Modification of Current Collectors (CCs) for Anode‐Free Sodium Batteries

**FIGURE 13:**
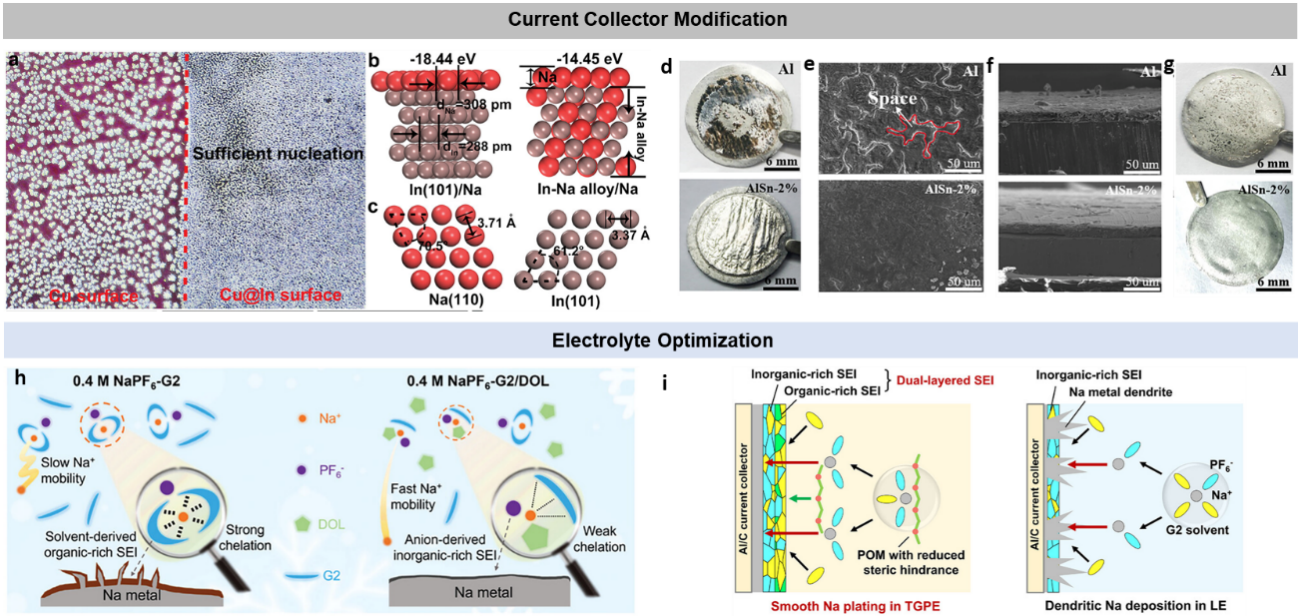
(a) Differences in sodium deposition between Cu and Cu‐In surfaces; (b, c) the models for the dominant facets of Na(110) and In(101), as well as the trends in the epitaxial growth of sodium on In(101) and Na‐In alloy; (d) optical images; (e) top‐view SEM images; (f) cross‐sectional SEM images of Al and AlSn‐2% after Na metal deposition of 1 mAh cm^−2^; (g) optical images of Al foil and AlSn‐2% after 100 cycles at 1 mAh cm^−^^2^; (h) schematic illustration of the mechanism of improved low temperature (LT) Na reversibility by the DOL‐diluted electrolyte; and (i) schematic illustration of utilizing a polymer with reduced local steric hindrance and Na^+^ solvation to control the formation of a weakly solvating polymer‐stabilized anion‐rich Na^+^ solvation structure and inorganic–organic dual‐layered interphase for smooth Na metal deposition. Adapted with permission from [259]. Copyright 2025, Wiley‐VCH; [265]. Copyright 2025, Wiley‐VCH; [270]. Copyright 2017, Wiley‐VCH; [271] Copyright 2025, Wiley‐VCH.

Similar to Li systems, modification of the CC with a sodiophilic layer is often performed to reduce the nucleation barrier (overpotential) of Na deposition and to support the growth of a uniform sodium layer stabilized by an SEI [258]. Both Cu and Al CCs have been used for AFSB assembly. In the case of Cu CC, the rapid formation of inactive Na and dendrite growth was attributed to the crystal mismatch and weak lattice force between the Cu CC and Na metal deposits. An indium coating on Cu CC with favorable crystal matching and lattice force lowered the nucleation barrier and resulted in homogeneous Na deposits from a 1 M NaPF_6_ in diglyme electrolyte, as shown in Figure 13a–c [259]. The potential of this approach was also demonstrated in full cells with a Na_4_Fe_2.91_(PO_4_)_2_(P_2_O_7_) (NFPP) cathode, which achieved 78.3% capacity retention after 320 cycles at a 2C charge rate [259].

Furthermore, it was shown that coating a Cu CC with a pre‐sodiated tin layer (Na_15_Sn_4_) reduces the overpotential from 31.8 mV to 11.3 mV, improves sodiophilicity, and regulates the Na plating from 1 M NaPF_6_ in EMC/PC (1:1 vol%) with 4% FEC. Operando optical microscopy at 5 mA cm^−2^ showed that Na dendrites appeared on bare Cu after 5 min, while dendrite‐free deposition of Na metal occurred on the Na_15_Sn_4_‐modified copper CC. A full cell with a NaNi_0.5_Mn_0.5_O_2_ cathode delivered a reversible capacity retention of 86.7% after 200 cycles at 0.5C [260].

Hu et al. developed a Cu CC coated with Prussian blue analogue (PBA). PBA acts as a fast Na‐ion conducting interlayer, regulating the Na^+^ flux to achieve uniform Na plating/stripping. The anode‐free full cell, consisting of a PBA‐modified negative CC and a Na_3_V_2_(PO_4_)_3_ cathode, achieved a reversible capacity retention of 86.84% after 150 cycles at 5C with a 1 M NaPF_6_ – diglyme electrolyte [261].

In another example, a coating based on a CeF_3_‐nanocarbon composite comprising a mesoporous nitrogen‐doped carbon shell was shown to effectively stabilize the plating and stripping of Na metal on a Cu CC. The mechanism was attributed to the formation of a NaF‐rich interphase that remains stable even under high depth of discharge (DOD) cycling conditions (90%). Furthermore, the metallic Ce sites with their strong PF_6_^−^ affinity facilitate Na^+^ desolvation by lowering the energy barrier for anion extraction, thereby increasing Na^+^ conductivity at the electrode/electrolyte interface. A full cell exhibited a reversible capacity retention of 83.6% after 100 cycles with a 1 M NaPF_6_ ‐diglyme electrolyte [262].

Modified Al CC were developed either using (inter)metallic materials that alloy with Na, such as tin, antimony and zinc, or using carbon coatings, in particular to disrupt the strong passivating layer of Al_2_O_3_ on the surface of Al CC [263].

Shi et al. showed that an interphase layer of Sn‐Cu nanoparticles on an Al CC produced by magnetron sputtering improves Na plating/stripping behavior because the nanoparticles provide uniform seeding sites with high sodiophilicity and support fast ion transport. The anode‐free full cell with Sn‐Cu‐coating on Al CC and an NVP cathode with 1 M NaPF_6_ in diglyme as electrolyte delivered 79% reversible capacity retention after 100 cycles at 5C [264].

Instead of a coating process, Li et al. showed that incorporating small amounts (2%) of tin into aluminum by melting and hot rolling in air significantly improves the performance of an AFSB with Al CC [265]. This was attributed to the formation of an eutectic Al–Sn alloy in which elemental Sn is present. It was suggested that elemental Sn acts as a lubricant, reducing volume changes during alloying and dealloying, smoothing the surface of the CC, and disrupting the passivation Al_2_O_3_ layer on the Al surface as shown in Figure 13d–g. The overpotential of Na plating was reported to be 11 mV, which is consistent with pre‐sodiated tin on Cu CC [260]. The full cells made of Al‐NVP (Na_3_V_2_(PO_4_)_3_)—AlSn‐2%‐NVP with 1 M NaPF_6_ in diglyme delivered a reversible capacity retention of 78.1% after 100 cycles at 5C [265].

The 3D structuring of CC is another approach to achieve stable sodium plating and stripping. 3D structures based on heteroatom‐doped (e.g., N, P, F, etc.) carbon fibers decorated with sodiophilic metal nanoparticles such as Ag, Zn and Sn have been reported [266]. The micro‐, meso‐, and macroporosity of carbon fiber‐based CCs has been achieved through templating with metal‐organic frameworks (MOFs), resulting in reduced structural stress during plating and stripping, as well as inhibition of dendrite growth by reducing local current density and homogenizing Na^+^ flux [267, 268]. In another work, 3D architectures were produced by edging Al foil with a slurry containing carbon nanotubes (CNTs) (3D architecture), carboxymethyl cellulose (CMC) (flexible binder), and NaOH (corrosive sodium source), resulting in a 3D‐structured NaAlO_2_ interphase with a high Na^+^ conductivity that is firmly attached to the electrically conducting CNT scaffold [269].

Wang et al. recently published a theoretical guide based on diffusion‐limited‐aggregation (DLA) theory, in which they identified the dielectric coefficient, ion mobility, and concentration changes as three key parameters for designing ion‐conductive current‐collector interfaces with high plating/stripping efficiency and long cycle life. The authors have experimentally demonstrated that a CNT‐based current collector with an interface with minimized dielectric coefficient, enhanced concentration changes (nano‐/micro‐Sb particles), and high ion mobility (sodium carboxymethylcellulose, CMC‐Na) exhibits a reversible capacity retention of 93.9% over 175 cycles at a rate of C/3 in a multilayer pouch cell with an NVP cathode and 1.0 M NaPF_6_ in monoglyme electrolyte [266].

### 5.2 Optimization of Liquid Electrolytes for Anode‐Free Sodium Batteries

In addition to modifying the negative CC, extensive work has been published on optimizing liquid electrolytes to promote stable plating and stripping of sodium [257, 272]. The typical solvents for Na‐metal and anode‐free cells are either ether‐based solvents such as 1,2‐dimethoxyethane (DME), diethylene glycol dimethyl ether (DEGDME, G2), tetraethylene glycoldimethyl ether (TEGDME, G4) and 1,3‐dioxolane (DOL), or ester‐based solvents such as ethylene carbonate (EC), diethyl carbonate (DEC), propylene carbonate (PC) and dimethyl carbonate (DMC). However, there are obstacles to the widespread use of carbonate‐based electrolytes in sodium metal batteries (SMBs), as a soluble, continuously growing SEI forms and electrode stability is insufficient. In contrast, ether‐based electrolytes, which are characterized by their stable solvation structure and chemical stability, are capable of forming high‐quality SEI and exhibit excellent compatibility with electrodes, resulting in highly reversible reactions and fast reaction kinetics. However, they have low oxidation stability and are therefore incompatible with most cathodes. Zhang et al. developed a novel electrolyte (1 M NaPF_6_ in a G2 and G4, 9:1 by volume) that enabled flat, dendrite‐free, and planar growth of sodium metal on the negative CC [257]. The introduction of G4 into the G2‐based electrolyte resulted in a new solvation structure that favored the formation of more stable SEI components [255, 270]. Hu et al. showed that the solvation structure of conventional 1 M NaPF_6_ in diglyme electrolyte can be modified by dilution with cyclic ether (1,3‐dioxolane, DOL). DOL diluents shield the Coulomb interaction between Na^+^ and PF_6_^−^ as well as the intermolecular forces of diglyme, resulting in anomalously high Na^+^‐ion conductivity (Figure 13h). DOL is part of the solvation shell and weakens the chelation of Na^+^ by diglyme, thereby enhancing desolvation. This results in a desired inorganic‐rich SEI with uniform composition and high Na^+^ conductivity [255]. The multilayer AFSB pouch cells, consisting of a NVP cathode, Al/C foils, and 0.4 M NaPF_6_‐G2/DOL electrolyte, retained 95% of their initial capacity over 100 cycles at −25°C. Overall, ether‐based electrolytes are the most promising for improving the performance of liquid electrolyte‐based AFSBs [270].

The most common salts for Na‐metal and anode‐free cells are NaPF_6_, NaClO_4_, NaTFSI, NaFSI, NaBF_4_, and NaSO_3_CF_3_. The choice of salt is influenced by its solubility in the chosen solvent, cost, expected performance, and environmental impact. Hu et al. developed an electrolyte containing boron salts (0.1 M NaBF_4_ and 0.9 M NaPF_6_ in G2). They observed that its decomposition products formed an amorphous SEI consisting of a mixture of organic and inorganic compounds. The strong and resilient SEI film effectively inhibits the formation of dead sodium and sodium dendrites and repairs the cracks formed during sodium metal deposition and stripping [255]. Recently, fluorine‐free salts such as Na tetrakisphenoxyborate (NaBO_4_C_24_H_20_) have been shown to enable highly reversible Na stripping and plating an Al CC even at high current densities [273, 274].

Although ether‐based electrolytes have shown promise in AFSBs, the multiple ether bonds in diglyme (G2) molecules strongly chelate Na^+^, reducing its mobility and increasing the desolvation energy barrier, which in turn limits cell kinetics [271]. Xu et al. developed a 1,3,5‐trioxane (TXE)‐based gel polymer electrolyte (TGPE) synthesized by in situ radical polymerization of TXE in the presence of G2 solvent and NaPF_6_. TXE polymerization results in polyoxymethylene (POM) with reduced local steric hindrance and Na^+^ solvation capability compared to G2 molecules, enabling the formation of an immobile TGPE with a weakly solvating, polymer‐stabilized, anion‐rich Na^+^ solvation structure. The resulting dual‐layered SEI consists of a NaF‐rich inner layer that homogenizes Na^+^ fluxes and an organic‐dominated outer layer that strengthens the interface to achieve highly reversible, dendrite‐free Na metal deposition based on POM, thereby reducing the irreversible loss of sodium during repeated plating and stripping (Figure 13i) [271].

In order for AFSB to become a mature technology in the future, its stability (both cycle life and calendric lifetime) must be significantly improved. Various failure modes and strategies for mitigating them have been presented in the current literature. However, the cycle numbers reported are still an order of magnitude lower (>100) than for sodium‐ion batteries with liquid electrolytes (>1000). In order to further improve the reversibility of sodium plating and stripping in liquid electrolytes, the reviewed approaches for modification of CCs and electrolytes should be further optimized. In addition, the investigation of favorable cycling and activation conditions (voltage window/ depth of discharge, current densities and temperatures) for AFSBs should be systematically investigated, along with the detailed chemical and optical analyses of the interphases formed during cell cycling and better understanding of sodium electrodeposition mechanism.

### 5.3 Anode‐Free Sodium Batteries (AFSBs) with Solid Electrolytes

As described for lithium systems in Section 4, replacing the liquid electrolyte in AFSBs with inorganic solid electrolytes has been investigated due to the inherent advantages, namely reduced flammability, higher mechanical modulus, and improved thermal stability. However, the somewhat lower ionic conductivity at room temperature and the high rigidity pose significant challenges for AFSBs with solid electrolytes. The main classes of inorganic solid electrolytes for AFSBs are: i) oxides (e.g., Na‐beta alumina, NASICONs, N5‐electrolytes glasses Na_5_SmSi_4_O_12_), ii) sulfides (e.g., Na_3_PS_4_, Na_3_SbS_4_), iii) halides/oxyhalides (NaM(O)Cl_4_ (M = Nb,Ta)), and iv) borohydrides (NaBH_4_). The advantages and disadvantages of the various solid electrolytes, as well as the latest developments in this field, have been discussed in various review articles [275, 276, 277, 278]. For the different classes of electrolytes, the inherent volume changes during sodium plating and stripping must be addressed by different strategies, as the volume changes lead to poor mechanical stability at the CC/SE interfaces. The void formation next to fracturing of the SE induced by the volume fluctuations during cycling have been identified as the most critical interfacial degradation mechanisms in AFSBs with SE [279].

Compared to anode‐free lithium all solid‐state batteries, anode‐free sodium batteries with SE have only recently been investigated experimentally [280]. In 2023, Ortmann et al. reported on the sodium plating at the copper‐Na_3.4_Zr_2_Si_2.4_P_0.6_O_12_ (NZSP) interface for the first time [281]. The authors demonstrated that the deposition of dense sodium layers with thicknesses of several micrometers at the Cu‐NZSP interface were comparable in quality to electroplated lithium films. However, the growth of small islands and whiskers could be observed leading to gap formation. Overall, the whisker growth did not lead to fracturing of the SE near the interface. However, it could not be excluded that sodium does nucleate inside the SE causing a high mechanical stress that leads to the formation of cracks, which was suggested to arise from the partial electronic conductivity of the SE. Moreover, it was found that the homogeneity of the sodium distribution on the copper electrode improves with increased current densities, whereas the stack pressure had a minor impact on the sodium distribution under the chosen experimental conditions [281].

In 2024, Deysher et al. published the following design principles for anode‐free sodium solid‐state batteries guiding future research in this nascent field [282, 283]: (i) an electrochemically stable or highly passivating electrolyte is needed to avoid the consumption of active sodium inventory due to the formation of an SEI; (ii) a robust solid–solid interface contact between the solid electrolyte and the current collector is needed for repeated sodium plating/stripping. Any void between the materials will prevent electron transfer hindering sodium metal deposition; (iii) a dense solid‐state electrolyte layer is needed to prevent the formation of pores, as cracks can promote the growth of metal filaments through the separator resulting in cell short circuiting, and (iv) the current collector needs to be highly dense unlike the emerging 3D structured, porous current collectors for liquid electrolytes.

Based on the design principles, the authors demonstrate that a full cell with a Na‐borohydride solid electrolyte, a pelletized aluminum current collector, and the application of stack pressure and elevated temperature enable highly reversible sodium plating and stripping.

The sodium anode‐free solid‐state battery full cell with a NaCrO_2_ cathode, Na_0.625_Y_0.25_Zr_0.75_Cl_4.375_ solid catholyte, Na‐borohydride electrolyte and pelletized aluminum current collector, exhibits stable cycling for several hundred cycles at 0.8 C and at 10 MPa and 40 °C. However, at room temperature the polarization was reported to be very high.

The wide variety of solid electrolytes, such as oxides, sulfides, halides/oxyhalides, borohydrides, for AFSBs with solid electrolytes recently reviewed by Huang et al. in combination with effective solid‐solid‐interface engineering paves the way for further optimization in the nascent field of AFSBs with SEs [284]. The external operational parameters, such as applied pressure and temperature during plating and stripping, will need to be investigated in detail and operando analytics to measure geometrical (e.g., dilatometry), structural, and chemical changes during cycling (plating and stripping) should be applied, to investigate failure mechanisms and to overcome the high polarization at room temperature [282]. A critical material property to be considered for future research into anode‐free batteries with solid electrolytes is the plastic flow during plating and stripping which causes the compression forces at the anode‐solid‐electrolyte interface to change. Fundamental works on the elastic, plastic, and creep mechanical properties of both lithium and sodium were reported by Masias et al. [248] and Wang et al. [249], respectively. Specifically, it was determined that Na is a significantly softer and weaker metal than Li mainly manifested in the experimentally measured elastic moduli, yield, and flow stresses. Sodium has a significantly higher tendency to creep (stress‐driven deformation). Two independent studies showed then that solid‐state metal batteries with Na‐β‐alumina could be cycled up to a much higher critical current density or at a lower stack pressure compared to Li/LLZO and Li/Li_6_PS_5_Cl cells [285, 286], which was mainly attributed to the higher creep rate of sodium but considering that other factors might play a role as well.

These studies are especially relevant for solid‐state batteries applying metallic foils as anodes. Anode‐free batteries share similar challenges with distinct differences, which are yet to be fully elucidated [287]. In anode‐free batteries, the plating and stripping of the alkali metals take place between the current collector and the solid electrolyte and the repeated plating and stripping during charging and discharging is highly dependent on the microstructure resulting from the initial plating cycle. In this case, additional factors like the dependence of the mechanical properties on the aspect ratio of the alkali metal coating and interfacial forces like friction and/or adhesion between the current collector and the solid electrolyte, which may hinder plastic flow of the electroplated metals, have to be considered and controlled besides the fundamental difference in plasticity of both alkali metals [249]. To date, it remains to be investigated how operando formed alkali metal films at the interface of specifically engineered substrates and various solid electrolytes in anode‐free batteries behave at various temperatures and stack pressures. The difference in melting point of both lithium and sodium leads to different plasticity at elevated temperatures and at different strain rates (current densities). Consequently, different mitigation strategies must be found to manage the ∼100% volume change upon fully charging and discharging by minimizing the external pressure (<1 MPa) and hence to arrive at practical and scalable solutions in the future. Initial results on metal anode batteries suggest that sodium‐based anode‐free batteries in combination with oxide solid electrolytes could benefit from its high plasticity, which may allow for operation at lower stack pressure and higher current densities. However, additional work is needed to stabilize the interface and hence attain long cycle and calendar life.

## 6 Anode‐Free Prototype Cells

The demonstration of prototype AFBs in multilayer (stack) format is a crucial factor in successfully bridging low TRL2‐3 knowledge generated and published by academic community to higher TRL4‐6 (A‐B‐samples) to attract industry players (battery manufactures, OEMs and end users). Reviewing publicly available information on AFB prototypes, the scientific and technological details are often not reported due to the high commercial interest and sensitivity of the topic to intellectual property. Table 5 provides an overview of the state of the development of AFB prototypes.

**TABLE 5:** Summary of anode‐free battery cell prototypes.

Company/Lab	Chemistry/System	AnodeCC	Modification	Cathode	Electrolyte/Separator	Areal capacity mAh/cm^2^	Cell description	Formation	Energy density	Electrochemical performance	Refs.
Samsung SDI	Li/SSB	SUS foil	Ag NP (D50 60 nm)/C (1:3), 5–10 µm, 8–16 mg Ah^−1^	LZO coated, LiNi_0.9_Co_0.05_Mn_0.05_O_2_, composite, 6.8 mAhcm^−2^, 100 µm	Li_6_PS_5_Cl, 40 µm	—	0.6 Ah pouch (stack 67×112 mm^2^)	0.1C/0.2C, 2.5‐4.25 V, 60°C, 2MPa	>900 Wh/L	60°C, 0.5C/0.5C, 1000 cycles, 2MPa	[136]
Samsung SDI	Li/SSB		Functional layer	Ni‐rich NCA	Sulfide based	—	Prismatic >20Ah	—	450 Wh/kg, 900 Wh/L	—	
Advanced Batteries Research Center	Li/SSB	10‐µm‐thick SUS foil (SUS430)	Ag materials, carbon black, and PVDF	Nd‐doped and coated by boric acid Li[Ni_0.89_Co_0.10_Al_0.01_]O_2_, Li_6_PS_5_Cl, PTFE, carbon black (80:18:1:1 wt%), 7.10 mAh cm^−2^, (32.0 mg_CAM_ cm^−2^)	Li_6_PS_5_Cl	7.10	Pouch (stack 20×20 mm^2^)	—	—	0.5 C (90 mA g^−1^) between 2.5 and 4.35 V (vs Li^+^/Li) at 60°C, 4 MPa	[288]
Quantum Scape	Li/Hybrid	—	—	CLL up to 5.6 mAh cm^−2^, catholyte with plasticizers	LLZO‐based film	5.6	24‐layer pouch (stack 60×75 & 70–85 mm^2^)	—	800 Wh/L (projection)	CLL 3.1, 25°C, 0.33C/0.5C, >1000 cycles (CR95%), 0.34 MPa CLL 3.1, 25°C, 1C/0.5C, 2000 cycles (CR80%) 0.0 MPa CLL 5.6, 45°C, 1C charge 0–80% SOC, <15 min, 0.07MPa	[297]
Quantum Scape	Li/Hybrid	—	—	CLL up to 6.2 mAh cm^−2^, catholyte with plasticizers	LLZO based film	6.2	Pouch, 21.6 Wh (84.5×65.6 & 4.6 mm^3^)	—	844 Wh/L, 301 Wh/kg	Charging from 10–80% SOC in <15 min at 45°C, low temperature performance down to 30°C, high‐power discharge up to 10C rate.	[289]
Lui et al.	Li/Liquid	Bare Cu	—	NMC811, 5.0 mAh cm^−2^, 23 mg cm^−2^.	2.0 M LiDFOB and 1.4 M LiBF4 in FEC/DEC (vol. ratio 1:2) 2.0 gAh‐1/12 µm polyethylene (PE) separator.	5.0	0.46 Ah, pouch.	Seed layer formation: 0°C, 36 s, 1C (0.05 mAh)	437 Wh/kg at stack level	74 cycles (CR 70%), 0.2C/0.5C, 25°C	[293]
Garcia‐Calvo et al.	Li/SSB	Cu	Ag NP / Carbon black (1:3 wt), PVdF	LiFePO_4_/C45/PEO‐LiTFSI, 0.54‐0.65 mAhcm^−2^, 2.0 g cc^−1^	PEO‐LiTFSI‐Al_2_O_3_, nanoparticles, 55–75 µm	0.54‐0.65	16 mAh (up to 0.45 Ah), stack size 50×60 cm^2^	—	—	60°C, 1 N m, DoD 100%, 2.5–3.8 V, 0.1C–0.1C, and charge cut off current 0.05C, 50 cycles (CR 54%)	[290]
Dahn et al.	Li/liquid	Bare Cu	—	Polycrystalline LiNi_0.8_Mn_0.1_Co_0.1_O_2_ (16 mg cm^−2^, 3.36 g cm^−3^)	1.2 M LiDFOB, 0.2 M LiBF4 (FEC:DEC vol.) 0.55 g 2M	3.47	0.25 Ah, 403025	—	—	40°C, 75 kPa, C/5 charge and C/2 discharge, 3.6‐4.5 V, 90 cycles (CR 80%)	[147]
Louli et al.	Li/liquid (high concentration)	Bare Cu	—	Single‐crystal LiNi_0.5_Mn_0.3_Co_0.2_O_2_ (16 mg cm^−2^, 3.5 g cm^−3^)	2 M LiDFOB. 1.4 M LiBF_4_ FEC:DEC (1:2 vol.), 0.5 mL (∼2.6 g Ah^−1^, 2.2 mL Ah^−1^)	3.1	0.23 Ah, 403025	40°C. 75 kPa, 1.25‐4.5 V, 0.1C/0.1C	900 Wh/L	40°C, 1170 kPa, C/5 charge and C/2 discharge, 3.6‐4.5 V, 200 cycles (CR 80%)	[170]
Wang et al.	Na/liquid	Al/C	Plasma treated	Na_3_V_2_(PO_4_)_3_, 15 mg/cm^2^, >100 µm	0.6 M NaOTF, 0.4 M NaBF_4_ in diethylene glycol dimethyl ether, 7 g Ah^−1^/polypropylene separator	0.5	1.79 Ah, 17 cathode layers of 5×9.5 cm^2^, 18 layers of 5.5×10 cm^2^ of anode	10 cycles, 0.05 mAcm^−2^, 1‐2 V	110 Wh/kg	−40°C, 0.05C, 2.8‐3.8 V, 50 cycles (CR 93.8%)	[294]
Willow et al.	Na/liquid	Al	4:1, Super‐P–Carboxymethylcellulose	Na_2_Fe[Fe(CN)_6_], 3.3–4.2 mg cm^−2^	1 M NaPF_6_ in diglyme, 550 µL	—	<0.1 Ah, 3 layers, 56×43 mm^2^	—	3 Ah projection: 282 Wh/kg and 454 Wh/L	25°C, 200 kPa, 2–3.8 V, 0.5C/0.5C, 200 cycles (63% CR),	[295]

*Note*: CR—capacity retention, CLL—cathode loading level (mAh cm^−2^). Areal capacities, expressed in mAh/cm^2^, were added when the information is available.

One of the most advanced solid‐state AFB prototypes to date was presented by Samsung SDI [136]. The multilayer solid‐state AFB in pouch cell format with a nominal capacity of 0.6 Ah based on surface‐modified NMC955, argyrodite Li_6_PS_5_Cl electrolyte and stainless steel current collector with lithiophilic Ag‐C nanocomposite coating demonstrated the volumetric energy density of 900 Wh L^−1^, 1000 depth cycles and an outstanding safety, being fully charged at a temperature of 210°C. Recently, Samsung SDI unveiled information on the plans for start of mass production in 2027 of solid state and AFB based on a high‐Ni NCA cathode, sulfide solid electrolyte and a novel anode with a functional layer. Cells will be produced in prismatic format with high energy density (>450 Wh kg^−1^, >900 WhL^−1^) and safety. Very recently, Park et al. demonstrated anode‐free pouch cell with dry processed cathode with ultrahigh loading of 7.10 mAh cm^−2^ (32.0 mg_CAM_ cm^−2^) based on Nd‐doped and coated by boric acid Li[Ni_0.89_Co_0.10_Al_0.01_]O_2_, Li_6_PS_5_Cl solid electrolyte and Ag‐C coated current collector. The multilayer pouch cell retained 80.2% of initial capacity among previously reported ASSBs using layered cathodes with Ni contents of 90% [288].

In 2023, QuantumScape reported the development of a 24‐layer Li‐free pouch cell prototypes based on high loading cathode (probably, containing a layered oxide), and LLZO‐based separator and undisclosed negative electrode, which demonstrated promising electrochemical performance in terms of cyclability and C‐rate capability over a wide temperature range. Late in 2025, the company presented QSE‐5 B‐sample with a very promising functionality (charging from 10%–80% state‐of‐charge in <15 min at 45°C, low temperature performance down to −30°C and high‐power discharge up to a 10C rate) [289]. The prototype preliminary passed safety tests with safety standards with hazard levels (HL) <4. On top of that, for the first time, the company revealed energy density (844 Wh L^−1^ and 301 Wh kg^−1^) values of the cell prototypes showing slightly superior values comparing to conventional LIB [136]. Interestingly, the QuantumScape cell prototypes can operate under minimal or even no applied stack pressure, likely because, to the best of our knowledge, the company has never claimed that their cells have all solid‐state architecture [289].

Noteworthy, reviews of recent patent applications show that many companies are working on the development of AFB in a stealth mode without revealing relevant technical details about the state of the development of prototypes.

In parallel, the academic community has also been intensively engaged in understanding and optimizing multilayer anode‐free battery cells, especially in pouch format. Garcia‐Calvo et al. reported on single‐layer anode free pouch cells with LiFePO_4_ cathode PEO‐LiTFSI‐Al_2_O_3_ composite polymer electrolyte and various coatings on Cu current collector [290]. The best performance was achieved with a 15 µm thick composite layer on the Cu containing Ag nanoparticles and carbon black in a 1:3 wt. ratio, doubling the initial discharge capacity (from 46 to 93 mAh g^−1^) compared to the bare Cu, and CE of over 99% after 50 cycles (capacity retention of 54%). Later, the same authors upscaled the developed cell design to a 0.45 Ah multilayer configuration (6 doubled‐sided LFP cathodes, 6 anodes with Ag/C coating). However, contrary to expectations, the electrochemical performance of the upscaled cell remained the same [290].

Dahn et al. published a series of articles in which they pointed out the importance of optimizing the liquid electrolyte formulation, pressure, formation conditions, cycling conditions, cathode choice, and other important aspects of the AFB technology [146, 147, 170, 291, 292]. The first breakthrough in electrochemical performance was achieved in the anode‐free 403025 pouch cell with dual salt electrolyte, which retained 80% of the original capacity after 90 cycles [147]. Later Louli et al. demonstrated an anode‐free Cu/NMC532 pouch cell with an extended cycle life up to 200 cycles at 40°C due to the use of high‐concentration dual‐salt electrolyte (2 M LiDFOB, 1.4 M LiBF_4_, FEC:DEC (1:2 vol)). Remarkably, 50 times cycled fully charged AFB with dual‐salt electrolyte successfully passed the nail penetration tests [170].

In 2023, Liu et al. demonstrated a 0.46 Ah prototype pouch cell based on NMC811 high loading cathode, bare Cu foil and polyethylene (PE) separator impregnated with liquid electrolyte composed by 2 M LiDFOB and 1.4 M LiBF_4_ in FEC/DEC (vol. ratio 1:2), with energy density of 437,8 Wh kg^−1^ at stack level reaching 70% of initial capacity after 74 cycles. As main innovation, authors proposed to apply an in situ implantation of a uniform and dense Li seed layer on the Cu current collector by reducing the temperature and increasing the current density to augment the nucleation overpotential, thereby forming an ultrathin and dense Li seed layer that facilitates subsequent high‐areal‐capacity Li plating/stripping with high reversibility [293].

**FIGURE 14:**
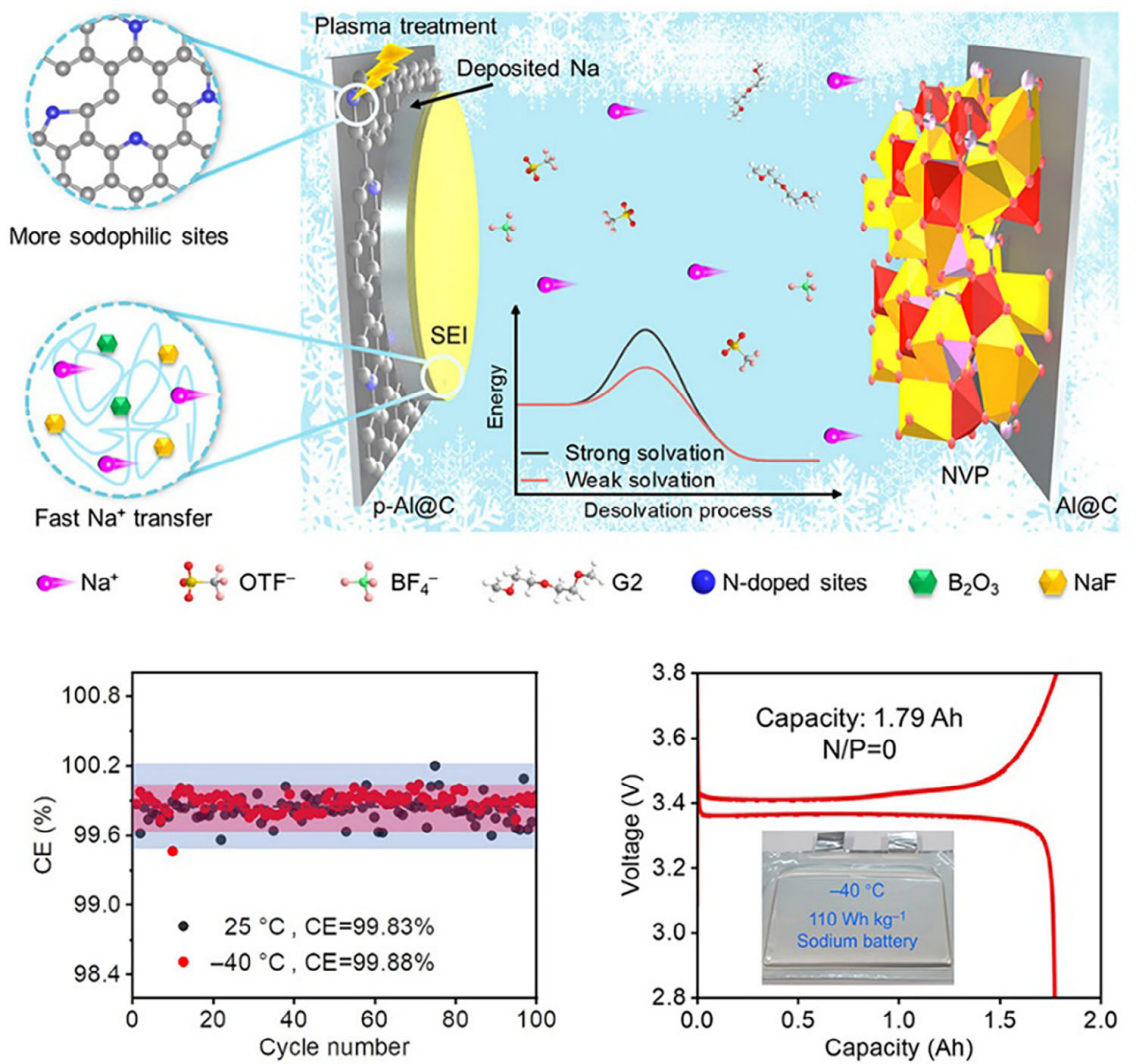
Design and electrochemical performance at −40°C of sodium anode‐free cell reported by Wang et al. Reproduced with permission [294]. Copyright 2024, Elsevier.

In 2024, Wang and coworkers reported a 1 Ah‐level anode‐free sodium battery (Figure 14) that delivers 110 Wh kg^−1^ at ‐40°C. The pouch cell is equipped with a plasma‐treated current collector (pAl@C), which has a sodiophilic N‐doped carbon surface that enables uniform sodium nucleation and growth, a weak‐solvation electrolyte, and a Na_3_V_2_(PO_4_)_3_ cathode that has capacity retention of 93.8% after 50 cycles [294].

Very recently, Willow et al. demonstrated single‐ and few‐layer format pouch cells (0.1 Ah) sodium‐ion anode free pouch cells based on a Prussian white cathode, anode coated by a mixture of carbon black and CMC, and a liquid electrolyte. 3‐layer pouch cells demonstrated promising cyclability maintaining ca. 63% of initial capacity after 200 cycles [295]. However, electrode characterization to probe the underlying operation and degradation mechanisms was not conducted, so while the results are promising, the viability of this AFSB chemistry still requires further validation.

Bree et al. [296]. demonstrated the production of 0.2 Wh Li AFB pouch cells based on LiMn_x_Fe_1−x_PO_4_ (LMFP) cathodes and Cu CC, using 0.6 M LiBF_4_ + 0.6 M LiDFOB in 1:2 FEC:DEC electrolyte, providing a 35% boost to energy density. However, a very short cycle life (approximately 30 cycles). The authors identified that Li inventory as the major capacity loss mechanism, demonstrated that the variation of the test protocol significantly affected cell degradation characteristics, while utilizing a higher Mn of 80% provided a further enhancement to energy density, at the cost of more rapid capacity loss associated with more Mn dissolution and worse kinetics [295].

To summarize, despite the limited information available, academic researchers and battery companies have reported the development of various AFBs in multilayer format. This fact shows that AFB technology has good prospects in terms of scalability. Interestingly, most of the reported chemistries are lithium, the cathode is a Ni‐rich NMC and the cell format is a pouch cell. There is no clear electrolyte favorite as AFBs with sulfides, solid oxide, solid polymer and liquid electrolytes have been reported. The developed cell prototypes with both liquid and solid electrolytes demonstrated very promising electrochemical performance. The prototypes with an energy density of >1000 Wh L^−1^ and cyclability >1000 cycles have so far been shown by cells based on solid electrolytes.

## 7 Conclusions and Outlook

AFBs were first introduced as a proof of concept more than 30 years ago. AFBs based on Li or Na metal anodes, which are electrodeposited in situ during the first formation cycle, with liquid or solid electrolytes, have already shown promising electrochemical performance at the pouch cell level, with favorable scalability to Ah capacities. While both liquid and solid electrolytes have demonstrated promising performance, only solid‐state systems have so far achieved an energy density >1000 Wh L^−^^1^ and cycle life beyond 1000 cycles, albeit at low charging rates and elevated temperatures. We emphasize that there is an urgent need to conduct systematic and comprehensive studies investigating practical anode‐free pouch cells with relevant capacities (>1 Ah) under relevant operational conditions. Furthermore, production‐friendly configurations, manufacturing processes, and robust forming procedures for AFBs with liquid and solid electrolytes need to be developed.

The reported successful results have sparked interest in this topic among the academic community, battery manufacturers, EV manufacturers, and OEMs. Based on the limited information available, several relevant players, including start‐ups, are actively working on the development of advanced anode‐free batteries. The intense activity in the field of intellectual property protection also demonstrates a concrete commitment to this area.

The anode‐free battery design offers several advantages. It significantly increases the volumetric and gravimetric energy density compared to today's LIBs and even compared to LMBs with >15‐20 µm thick Li metal anodes (in the ultimate configuration when using uncoated current collector). It eliminates the need for anode materials with a high environmental footprint (e.g., graphite and silicon) and avoids the production and handling of reactive alkali metal foils. AFB technology has the potential to reduce both material and processing costs and improve overall sustainability (by eliminating Li metal, which is considered a critical raw material in the EU), increase supply chain stability (by reducing vulnerability to export and geopolitical restrictions), while remaining compatible with existing lithium‐ion battery manufacturing lines.

Significant challenges remain for both electrolyte systems. For solid‐state systems, the challenges include achieving high ionic conductivity at room temperature, reducing interfacial resistance, preventing dendrites formation, and manufacturing solid electrolytes on a large scale. Liquid‐based systems, on the other hand, can be implemented in existing LIB production lines. However, challenges remain in achieving application‐relevant electrochemical performance. As evidenced by the current literature, the focus to date has primarily been on electrolyte composition and interface properties, particularly the SEI. Although these aspects are critical, we believe that the CC interface, specifically its surface chemistry, lithiophilicity, and role in governing the electrodeposition mechanism, deserves greater attention.

Both liquid and solid electrolyte AFB configurations suffer from similar degradation mechanisms, mainly due to Li inventory loss, with dendrites formation, interface instability, and soft short circuits playing an important role. While Li loss due to SEI breakdown and healing has been extensively studied, there is little research on the interplay between these mechanisms—in particular, the combined effects of Li galvanic corrosion, dead Li formation, and degradation caused by short circuits.

Despite significant advances in AFB technology, a better understanding of the Li metal plating and stripping process (or alloying/dealloying in hybrid scenarios) and the identification of promising lithiophilic materials remain essential for practical realization of AFBs. Particular attention should be paid to the initial formation cycle, as the conditions under which the metal anode forms greatly influence anode‐free cell performance, reversibility, and long‐term stability.

Comparing fundamental studies and semi‐commercial AFB prototypes remains difficult due to inconsistent reporting of key metrics such as Coulombic efficiency, cathode loading, areal capacity, cycling rate, depth of charge, Li/Na reservoir, and effective N/P ratio. Consequently, meaningful quantitative comparisons are largely impossible, and the field remains limited to qualitative, case‐by‐case assessments of full cell AFB demonstrators. Moreover, raw data is typically not accessible, limiting the ability to use contemporary automation tools to survey and predict the most promising configurations. We consider this a major obstacle to the development of AFBs, as it hinders understanding of the state‐of‐the‐art and complicates the design of effective technological improvements.

It is noteworthy that many of the highest‐performing AFB cells reported to date are based on complex architectures with multiple modifications, including custom current collectors with special coatings, which however may reduce energy density, compromise sustainability, complicate reproducibility, and makes it difficult to extract or identify key performance factors.

In addition to fundamental understanding, product‐specific detailed life cycle analysis (LCA) and life cycle cost analysis (LCC) should be conducted to evaluate the impact of switching from LIB and LMB to AFB and to demonstrate the feasibility of this technology, taking into account the necessary modifications on the anode side. In particular, the impact of the type and amount of lithophilic coating, as well as the use of lithophilic current collectors with new architectures, should be carefully assessed through LCA and LCC.

While AFBs may not be able to completely replace lithium‐ion batteries, they have the potential to have a significant impact on the most energy‐intensive battery applications with relatively short lifespan. Furthermore, it is important to recognize the immense potential of beyond‐Li AFB technology. This is particularly true in the context of the emergence of Na‐based AFBs, which, although still less developed, appear promising.

## Funding

A.K., S.C., E.A., and D.O. are grateful to the European Union for the financial support of the work performed within the HEU project SOLVE (Grant no. 101147094). The views and opinions expressed are those of the author(s) only and do not necessarily reflect those of the European Union. Neither the European Union nor the granting authority (CINEA) can be held responsible for them. As a part of the DESTINY PhD program, this project received funding from the EU's Horizon2020 research and innovation program under the Marie Sklodowska Curie Actions COFUND (Grant Agreement #945357). Grant PID2022‐137626OB‐C33 and PLEC2022‐009412 funded By MICIU/AEI/10.13039/501100011033 and By “ERDF/EU”. S.M. and X.S. fratefully acknowledge funding by the Royal Society University Research Fellowship (URF, URF∖R1∖231513), Faraday Institution Degradation (FIRG082) and SafeBatt (FIRG086) projects, and Yusuf Hamied Department of Chemistry, University of Cambridge. ABG gratefully acknowledge funding from the Icelandic Research Fund, grant numbers 217896 and 2511109. P.S., M.F. and D.FR. gratefully acknowledge funding by the Federal Ministry of Research, Technology and Space under the project HIPOBAT (13XP0611A), 13XP0510A (CatSE2), 13XP0434A and 13XP0432B (FestBatt 2‐Oxid).

## Conflicts of Interest

The authors declare no conflicts of interest.

## Data Availability

The authors have nothing to report.

## References

[1] European Commission, “Critical Raw Materials Resilience: Charting a Path towards Greater Security and Sustainability”. 2020. COM(2020) 474 final. Brussels: European Commission, https://eur‐lex.europa.eu/legal‐content/EN/TXT/?uri=celex%3A52020DC0474.

[2] T. Li , R. Gao , X. Wang , et al., “Precise Chemical Lithiation: a Pathway to Superior Li‐Enriched Li_1+x_NCM523 Cathodes for Long Life Anode‐Free Li Metal Batteries,” Journal of the American Chemical Society 147 (2025): 29895–29907, 10.1021/jacs.5c06681. 40765375

[3] S. Wang , Y. Wang , Z. Ouyang , et al., “Molecular Engineering of Two‐dimensional Polyamide Interphase Layers for Anode‐free Lithium Metal Batteries,” Nature Materials 24 (2025): 1957–1967, 10.1038/s41563-025-02339-y. 40921748

[4] J. Xu , K. Qu , X. Li , et al., “Highly Reversible Anode‐Free Lithium Metal Batteries Enabled by Porous Organic Cages with Subnano Lithiophilic Triangular Windows,” ACS Nano 19 (2025): 2936–2943, 10.1021/acsnano.4c16906. 39779299

[5] Z. Ouyang , S. Wang , Y. Wang , et al., “An Ultralight Composite Current Collector Enabling High‐Energy‐Density and High‐Rate Anode‐Free Lithium Metal Battery,” Advanced Materials 36 (2024): 2407648, 10.1002/adma.202407648. 38900369

[6] Y. Chen , S. Wang , T. Wang , X. Wang , H. Sun , and C. Zhu , “Preparation of Cyclic Olefin Polymers via Group Transfer Radical Cyclopolymerization for High Performance in Anode‐Free Batteries,” Angewandte Chemie International Edition 64 (2025): 202507557, 10.1002/anie.202507557. 40305124

[7] Proteo Data Analytics. 2024, CIDETEC. Proteo Data Analytics: Data Analytics Module of the PROTEO Battery Development Platform. CIDETEC Energy Storage. Donostia‐San Sebastián, Spain. https://cidetec.es/en/proteo‐a‐tool‐that‐integrates‐the‐digital‐era‐of‐batteries‐with‐the‐real‐world/?utm_source=chatgpt.com.

[8] M. Doyle , T. F. Fuller , and J. Newman , “Modeling of Galvanostatic Charge and Discharge of the Lithium/Polymer/Insertion Cell,” Journal of The Electrochemical Society 140 (1993): 1526–1533, 10.1149/1.2221597.

[9] S. Menkin , C. A. O'Keefe , A. B. Gunnarsdóttir , et al., “Toward an Understanding of SEI Formation and Lithium Plating on Copper in Anode‐Free Batteries,” The Journal of Physical Chemistry C 125 (2021): 16719–16732, 10.1021/acs.jpcc.1c03877. PMC8392351 34476038

[10] A. J. Sanchez , E. Kazyak , Y. Chen , K.‐H. Chen , E. R. Pattison , and N. P. Dasgupta , “Plan‐View Operando Video Microscopy of Li Metal Anodes: Identifying the Coupled Relationships among Nucleation, Morphology, and Reversibility,” ACS Energy Letters 5 (2020): 994–1004, 10.1021/acsenergylett.0c00215.

[11] A. Pei , G. Zheng , F. Shi , Y. Li , and Y. Cui , “Nanoscale Nucleation and Growth of Electrodeposited Lithium Metal,” Nano Letters 17 (2017): 1132–1139, 10.1021/acs.nanolett.6b04755. 28072543

[12] E. R. Cooper , M. Li , I. Gentle , Q. Xia , and R. Knibbe , “A Deeper Understanding of Metal Nucleation and Growth in Rechargeable Metal Batteries through Theory and Experiment,” Angewandte Chemie 135 (2023): 202309247, 10.1002/ange.202309247. 37735095

[13] B. Scharifker and G. Hills , “Theoretical and Experimental Studies of Multiple Nucleation,” Electrochimica Acta 28 (1983): 879–889, 10.1016/0013-4686(83)85163-9.

[14] C. Fang , X. Wang , and Y. S. Meng , “Key Issues Hindering a Practical Lithium‐metal Anode,” Trends in Chemistry 1 (2019): 152–158, 10.1016/j.trechm.2019.02.015.

[15] S. Nanda , A. Gupta , and A. Manthiram , “Anode‐Free Full Cells: a Pathway to High‐Energy Density Lithium‐Metal Batteries,” Advanced Energy Materials (2020), 10.1002/aenm.202000804.

[16] C. W. Gustavo , M. Hobold , K. Steinberg , Y. Li , and B. M. Gallant , “High Lithium Oxide Prevalence in the Lithium Solid–electrolyte Interphase for High Coulombic Efficiency",” Nature Energy 9 (2024): 580–591, 10.1038/s41560-024-01494-x.

[17] S. Menkin , J. B. Fritzke , R. Larner , et al., “Insights into Soft Short Circuit‐Based Degradation of Lithium Metal Batteries,” Faraday Discussions 248 (2024): 277–297, 10.1039/D3FD00101F. 37870402 PMC10823489

[18] J. B. Fritzke , J. H. J. Ellison , L. Brazel , G. Horwitz , S. Menkin , and C. P. Grey , “Spiers Memorial Lecture: Lithium Air Batteries–tracking Function and Failure,” Faraday Discussions 248 (2024): 9–28, 10.1039/D3FD00154G. 38105743 PMC10823487

[19] K. S. C. Michael , J. Counihan , P. Barai , et al., “The Phantom Menace of Dynamic Soft‐shorts in Solid‐state Battery Research,” Joule 8 (2024): 64–90, 10.1016/j.joule.2023.11.007.

[20] D. Fouchard and L. Lechner , “Analysis of Safety and Reliability in Secondary Lithium Batteries,” Electrochimica acta 38 (1993): 1193–1198, 10.1016/0013-4686(93)80049-6.

[21] S. B. Paul Albertus , S. Litzelman , and A. Newman , “Status and Challenges in Enabling the Lithium Metal Electrode for High‐energy and Low‐cost Rechargeable Batteries,” Nature Energy 3 (2018): 16–21, 10.1038/s41560-017-0047-2.

[22] S. Drvarič Talian , G. Kapun , J. Moškon , R. Dominko , and M. Gaberšček , “Operando Impedance Spectroscopy with Combined Dynamic Measurements and Overvoltage Analysis in Lithium Metal Batteries,” Nature Communications 16 (2025): 2030, 10.1038/s41467-025-57256-0. PMC11868597 40016234

[23] G. Wang , Y. Sun , K. Pang , B. Li , X. Han , and Y. Zheng , “Quantitative Diagnosis of the Soft Short Circuit for LiFePO4 Battery Packs between Voltage Plateaus,” Journal of Energy Storage 61 (2023): 106683, 10.1016/j.est.2023.106683.

[24] Y. Xu , X. Ge , W. Shen , and R. Yang , “A Soft Short‐circuit Diagnosis Method for Lithium‐Ion Battery Packs in Electric Vehicles,” IEEE Transactions on Power Electronics 37 (2022): 8572–8581, 10.1109/TPEL.2022.3151620.

[25] I. Yoshimatsu , T. Hirai , and J. Yamaki , “Lithium Electrode Morphology during Cycling in Lithium Cells,” Cheminform 20 (1989): 2422–2427, 10.1149/1.2095351.

[26] J. Steiger , D. Kramer , and R. Mönig , “Microscopic Observations of the Formation, Growth and Shrinkage of Lithium Moss during Electrodeposition and Dissolution,” Electrochimica Acta 136 (2014): 529–536, 10.1016/j.electacta.2014.05.120.

[27] Y. S. Cohen , Y. Cohen , and D. Aurbach , “Micromorphological Studies of Lithium Electrodes in Alkyl Carbonate Solutions Using in Situ Atomic Force Microscopy,” The Journal of Physical Chemistry B 104 (2000): 12282–12291, 10.1021/jp002526b.

[28] S. Jurng , Z. L. Brown , J. Kim , and B. L. Lucht , “Effect of Electrolyte on the Nanostructure of the Solid Electrolyte Interphase (SEI) and Performance of Lithium Metal Anodes,” Energy & Environmental Science 11 (2018): 2600–2608, 10.1039/C8EE00364E.

[29] X.‐Q. Zhang , X. Chen , X.‐B. Cheng , et al., “Highly Stable Lithium Metal Batteries Enabled by Regulating the Solvation of Lithium Ions in Nonaqueous Electrolytes,” Angewandte Chemie International Edition 57 (2018): 5301–5305, 10.1002/anie.201801513. 29465827

[30] J. Qian , W. Xu , P. Bhattacharya , et al., “Dendrite‐free Li Deposition Using Trace‐amounts of Water as an Electrolyte Additive,” Nano Energy 15 (2015): 135–144, 10.1016/j.nanoen.2015.04.009.

[31] J. Zheng , M. H. Engelhard , D. Mei , et al., “Electrolyte Additive Enabled Fast Charging and Stable Cycling Lithium Metal Batteries,” Nature Energy 2 (2017): 1–8, 10.1038/nenergy.2017.12.

[32] E. Markevich , G. Salitra , F. Chesneau , M. Schmidt , and D. Aurbach , “Very Stable Lithium Metal Stripping–plating at a High Rate and High Areal Capacity in Fluoroethylene Carbonate‐based Organic Electrolyte Solution,” ACS Energy Letters 2 (2017): 1321–1326, 10.1021/acsenergylett.7b00300.

[33] K. N. Wood , M. Noked , and N. P. Dasgupta , “Lithium Metal Anodes: Toward an Improved Understanding of Coupled Morphological, Electrochemical, and Mechanical Behavior,” ACS Energy Letters 2 (2017): 664–672, 10.1021/acsenergylett.6b00650.

[34] B. Liu , J.‐G. Zhang , and W. Xu , “Advancing Lithium Metal Batteries,” Joule 2 (2018): 833–845, 10.1016/j.joule.2018.03.008.

[35] E. Peled and S. Menkin , “SEI: Past, Present and Future,” Journal of The Electrochemical Society 164 (2017): A1703, 10.1149/2.1441707jes.

[36] J. Zheng , M. S. Kim , Z. Tu , S. Choudhury , T. Tang , and L. A. Archer , “Regulating Electrodeposition Morphology of Lithium: towards Commercially Relevant Secondary Li Metal Batteries,” Chemical Society Reviews 49 (2020): 2701–2750, 10.1039/C9CS00883G. 32232259

[37] X. Wang , M. Zhang , J. Alvarado , et al., “New Insights on the Structure of Electrochemically Deposited Lithium Metal and Its Solid Electrolyte Interphases via Cryogenic TEM,” Nano letters 17 (2017): 7606–7612, 10.1021/acs.nanolett.7b03606. 29090936

[38] M. He , R. Guo , G. M. Hobold , H. Gao , and B. M. Gallant , “The Intrinsic Behavior of Lithium Fluoride in Solid Electrolyte Interphases on Lithium,” Proceedings of the National Academy of Sciences 117 (2020): 73–79, 10.1073/pnas.1911017116. PMC6955333 31848237

[39] H. J. Chang , A. J. Ilott , N. M. Trease , M. Mohammadi , A. Jerschow , and C. P. Grey , “Correlating Microstructural Lithium Metal Growth with Electrolyte Salt Depletion in Lithium Batteries Using 7Li MRI,” Journal of the American Chemical Society 137 (2015): 15209–15216, 10.1021/jacs.5b09385. 26524078

[40] K. Nishikawa , T. Mori , T. Nishida , Y. Fukunaka , and M. Rosso , “Li Dendrite Growth and Li^+^ Ionic Mass Transfer Phenomenon",” Journal of electroanalytical chemistry 661 (2011): 84–89, 10.1016/j.jelechem.2011.06.035.

[41] G. Yoon , S. Moon , G. Ceder , and K. Kang , “Deposition and Stripping Behavior of Lithium Metal in Electrochemical System: Continuum Mechanics Study,” Chemistry of Materials 30 (2018): 6769–6776, 10.1021/acs.chemmater.8b02623.

[42] K. Nishikawa , T. Mori , T. Nishida , Y. Fukunaka , M. Rosso , and T. Homma , “In Situ Observation of Dendrite Growth of Electrodeposited Li Metal,” Journal of The Electrochemical Society 157 (2010): A1212–A1217, 10.1149/1.3486468.

[43] A. Kushima , K. P. So , C. Su , et al., “Liquid Cell Transmission Electron Microscopy Observation of Lithium Metal Growth and Dissolution: Root Growth, Dead Lithium and Lithium Flotsams,” Nano Energy 32 (2017): 271–279, 10.1016/j.nanoen.2016.12.001.

[44] O. Crowther and A. C. West , “Effect of Electrolyte Composition on Lithium Dendrite Growth,” Journal of the Electrochemical Society 155 (2008): A806–A811, 10.1149/1.2969424.

[45] J.‐I. Yamaki , S.‐I. Tobishima , K. Hayashi , Keiichi Saito , Y. Nemoto , and M. Arakawa , “A Consideration of the Morphology of Electrochemically Deposited Lithium in an Organic Electrolyte,” Journal of Power Sources 74 (1998): 219–227, 10.1016/S03787753(98)000676.

[46] D. Lu , Y. Shao , T. Lozano , et al., “Failure Mechanism for Fast‐Charged Lithium Metal Batteries with Liquid Electrolytes,” Advanced Energy Materials 5 (2015): 1400993, 10.1002/aenm.201400993.

[47] K.‐H. Chen , K. N. Wood , E. Kazyak , et al., “Dead Lithium: Mass Transport Effects on Voltage, Capacity, and Failure of Lithium Metal Anodes,” Journal of Materials Chemistry A 5 (2017): 11671–11681, 10.1039/C7TA00371D.

[48] C.‐J. Huang , B. Thirumalraj , H.‐C. Tao , et al., “Decoupling the Origins of Irreversible Coulombic Efficiency in Anode‐free Lithium Metal Batteries,” Nature Communications 12 (2021): 1452, 10.1038/s41467-021-21683-6. PMC7933276 33664259

[49] W. Dachraoui , R.‐S. Kühnel , C. Battaglia , and R. Erni , “Nucleation, Growth and Dissolution of Li Metal Dendrites and the Formation of Dead Li in Li‐ion Batteries Investigated by Operando Electrochemical Liquid Cell Scanning Transmission Electron Microscopy,” Nano Energy 130 (2024): 110086, 10.1016/j.nanoen.2024.110086.

[50] I. Lindsey , C. Mondl , and X. Meng , “In Situ and Operando Microscopy Studies on Lithium Metal Anodes: a Review,” Energy Advances (2026), 10.1039/D5YA00240K.

[51] T. L. Chengbin Jin , O. Sheng , M. Li , et al., “Rejuvenating Dead Lithium Supply in Lithium Metal Anodes by Iodine Redox,” Nature Energy 6 (2021): 378–387, 10.1038/s41560-021-00789-7.

[52] T. L. Mengting Zheng , J. Wu , X. Tao , Z. Li , S. Zhang , and J. Lu , “Voltage‐Induced Bromide Redox Enables Capacity Restoration of Fast‐Charging Batteries,” Advanced Materials 37 (2025): 2414207–2414217, 10.1002/adma.202414207. 39707696

[53] M. Weret , S.‐K. JiangKassie , N. Shitaw , et al., “Reviving Inactive Lithium and Stabilizing Lithium Deposition for Improving the Performance of Anode‐Free Lithium–Sulfur Batteries,” ACS Energy Letters 8 (2023): 2817–2823, 10.1021/acsenergylett.3c00622.

[54] L.‐L. Su , M.‐X. Wu , S.‐Y. Sun , et al., “Long‐Cycling Lithium–Sulfur Batteries Enabled by Reactivating Inactive Lithium,” ACS Energy Letters 10 (2025): 313–319, 10.1021/acsenergylett.4c03177.

[55] H. L. Qianya Li , F. Wu , L. Li , Y. Ye , and R. Chen , “Recent Advances and Opportunities in Reactivating Inactive Lithium in Batteries,” Angewandte Chemie 63 (2024): 1–12, 10.1002/anie.202404554. 38563638

[56] A. B. Gunnarsdóttir , C. V. Amanchukwu , S. Menkin , and C. P. Grey , “Noninvasive in Situ NMR Study of “Dead Lithium” Formation and Lithium Corrosion in Full‐Cell Lithium Metal Batteries,” Journal of the American Chemical Society 142 (2020): 20814–20827, 10.1021/jacs.0c10258. 33226793 PMC7729915

[57] D. Lin , Y. Liu , Y. Li , et al., “Fast Galvanic Lithium Corrosion Involving a Kirkendall‐type Mechanism,” Nature Chemistry 11 (2019): 382–389, 10.1038/s4155701802038. 30664717

[58] A. Kolesnikov , M. Kolek , J. F. Dohmann , et al., “Galvanic Corrosion of Lithium‐powder‐based Electrodes,” Advanced Energy Materials 10 (2020): 2000017, 10.1002/aenm.202000017.

[59] Z. Yu , Y. Cui , and Z. Bao , “Design Principles of Artificial Solid Electrolyte Interphases for Lithium‐metal Anodes,” Cell Reports Physical Science 1, no. 7 (2020): 100119, 10.1016/j.xcrp.2020.100119.

[60] D. T. Boyle , W. Huang , H. Wang , et al., “Corrosion of Lithium Metal Anodes during Calendar Ageing and Its Microscopic Origins,” Nature Energy 6 (2021): 487–494, 10.1038/s41560021007879.

[61] S. T. Oyakhire , S. C. Kim , W. Zhang , S. B. Shuchi , Y. Cui , and S. F. Bent , “Galvanic Corrosion Underlies Coulombic Efficiency Differences in High‐performing Lithium Metal Battery Electrolytes,” Energy & Environmental Science 18 (2025): 4847–4858, 10.1039/D5EE00071H.

[62] W. Zhang , P. Sayavong , X. Xiao , et al., “Recovery of Isolated Lithium Through Discharged state Calendar Ageing,” Nature 626 (2024): 306–312, 10.1038/s41586-023-06992-8. 38326593

[63] S. G. R. Laura , C. Merrill , K. L. Jungjohann , and K. L. Harrison , “Uncovering the Relationship between Aging and Cycling on Lithium Metal Battery Self‐Discharge,” ACS Applied Energy Materials 4 (2021): 7589–7598, 10.1021/acsaem.1c00874.

[64] C. Z. Qidi Wang , S. Wang , J. Wang , et al., “Interphase Design for Lithium‐Metal Anodes,” Journal of the American Chemical Society 147 (2025): 9365–9377, 10.1021/jacs.4c15759. 40053684

[65] K. L. Jungjohann , R. N. Gannon , S. Goriparti , et al., “Cryogenic Laser Ablation Reveals Short‐Circuit Mechanism in Lithium Metal Batteries,” ACS Energy Letters 6 (2021): 2138–2144, 10.1021/acsenergylett.1c00509.

[66] M. Guan , Z. Wei , R. Liu , et al., “Systematic Assessment of Coulombic Efficiency in Anode‐Free and Lithium Metal Batteries,” Small Methods 10, no. 2 (2025): 2500923, 10.1002/smtd.202500923. 40599129

[67] M. Abdollahifar and A. Paolella , “Anode‐Free Batteries: the Energy Density Prize and the Stability Paradox,” ACS Energy Letters 11 (2025): 1323–1348, 10.1021/acsenergylett.5c03401.

[68] Y. Wang , Y. Liu , M. Nguyen , et al., “Stable Anode‐Free All‐Solid‐State Lithium Battery through Tuned Metal Wetting on the Copper Current Collector,” Advanced Materials 35 (2023): 2206762, 10.1002/adma.202206762. 36445936

[69] Z. Liu , S. Bai , S. Burke , et al., “FIB‐SEM: Emerging Multimodal/Multiscale Characterization Techniques for Advanced Battery Development,” Chemical Reviews 125 (2025): 5228–5281, 10.1021/acs.chemrev.4c00831. 40311073

[70] T. Fuchs , T. Ortmann , J. Becker , et al., “Imaging the Microstructure of Lithium and Sodium Metal in Anode‐free Solid‐state Batteries Using Electron Backscatter Diffraction,” Nature Materials 23 (2024): 1678–1685, 10.1038/s41563-024-02006-8. 39313556 PMC11599044

[71] Z. Li , X. Huang , L. Kong , et al., “Gradient Nano‐recipes to Guide Lithium Deposition in a Tunable Reservoir for Anode‐free Batteries,” Energy Storage Materials 45 (2022): 40–47, 10.1016/j.ensm.2021.11.037.

[72] Y. Li , Y. Li , A. Pei , et al., “Atomic Structure of Sensitive Battery Materials and Interfaces Revealed by Cryo–electron Microscopy,” Science 358 (2017): 506–510, 10.1126/science.aam6014. 29074771

[73] S. Wang , S. Li , X. Chen , et al., “Depth‐Resolved Probing of Native Solid Electrolyte Interphase Formation and Dynamics in Li Metal Batteries by Cryogenic X‐Ray Photoelectron Spectroscopy,” Journal of the American Chemical Society 147 (2025): 38069–38077, 10.1021/jacs.5c09519. 40833285

[74] C. Fang , J. Li , M. Zhang , et al., “Quantifying Inactive Lithium in Lithium Metal Batteries,” Nature 572 (2019): 511–515, 10.1038/s41586-019-1481-z. 31435056

[75] G. M. Gallant , “Quantifying Capacity Loss Mechanisms of Li Metal Anodes beyond Inactive Li0,” ACS Energy Letters 7 (2022): 3458–3466, 10.1021/acsenergylett.2c01845.

[76] M. A. Hope , B. L. D. Rinkel , A. B. Gunnarsdóttir , et al., “Selective NMR Observation of the SEI–metal Interface by Dynamic Nuclear Polarisation from Lithium Metal,” Nature Communications 11 (2224), 10.1038/s41467-020-16114-x. PMC7203113 32376916

[77] A. Svirinovsky‐Arbeli , M. Juelsholt , R. May , Y. Kwon , and L. E. Marbella , “Using NMR Spectroscopy to Link Structure to Function at the Li Solid Electrolyte Interphase,” Joule 8 (2024): 1919–1935, 10.1016/j.joule.2024.04.016.

[78] Y.‐C. Hsieh , M. Leißing , S. Nowak , B.‐J. Hwang , M. Winter , and G. Brunklaus , “Quantification of Dead Lithium via in Situ Nuclear Magnetic Resonance Spectroscopy,” Cell Reports Physical Science 1 (2020): 100139, 10.1016/j.xcrp.2020.100139.

[79] A. S.‐A. Yongbeom Kwon , J. C. Hestenes , P. J. Buitrago , et al., “Elucidating the Role of Cathode Identity: Voltage‐dependent Reversibility of Anode‐free Batteries,” Chemistry (Weinheim An Der Bergstrasse, Germany) 10 (2024): 3159–3183, 10.1016/j.chempr.2024.06.008.

[80] J.‐H. Cheng , A. A. Assegie , C.‐J. Huang , et al., “Visualization of Lithium Plating and Stripping via in Operando Transmission X‐ray Microscopy,” The Journal of Physical Chemistry C 121 (2017): 7761–7766, 10.1021/acs.jpcc.7b01414.

[81] M. Sadd , S. Xiong , J. R. Bowen , F. Marone , and A. Matic , “Investigating Microstructure Evolution of Lithium Metal during Plating and Stripping via Operando X‐ray Tomographic Microscopy,” Nature Communications 14 (2023): 854, 10.1038/s41467-023-36568-z. PMC9931753 36792892

[82] S. G. Yoon , B. S. Vishnugopi , D. L. Nelson , et al., “Interface Morphogenesis with a Deformable Secondary Phase in Solid‐state Lithium Batteries,” Science 388 (2025): 1062–1068, 10.1126/science.adt5229. 40472090

[83] J. A. Lewis , F. J. Q. Cortes , Y. Liu , et al., “Linking Void and Interphase Evolution to Electrochemistry in Solid‐state Batteries Using Operando X‐ray Tomography,” Nature Materials 20 (2021): 503–510, 10.1038/s41563-020-00903-2. 33510445

[84] S. E. Sandoval , D. L. Nelson , H. Sridhara , et al., “Visualizing Diverse Lithium Growth and Stripping Behaviors in Anode‐free Solid‐state Batteries with Operando X‐ray Tomography,” EES Batteries 1 (2025): 1809–1821, 10.1039/D5EB00111K.

[85] J. S. Lowe , U. Janakiraman , G. Less , R. Kerns , and N. S. Muyanja , “Using X‐ray Microscopy to Probe Failure Mechanisms in Anode‐free Cells: an Industry Perspective,” ECS Advances 3 (2024): 040501, 10.1149/2754-2734/ad959c.

[86] C.‐J. Huang , J. A. S. Oh , M. Vicencio , et al., “X‐ray Micro‐Computed Tomography for Structural Analysis of all‐Solid‐State Battery at Pouch Cell Level,” ACS Energy Letters 10 (2025): 3459–3470, 10.1021/acsenergylett.5c00956. 40672128 PMC12261322

[87] J. O. Majasan , J. B. Robinson , R. E. Owen , et al., “Recent Advances in Acoustic Diagnostics for Electrochemical Power Systems,” Journal of Physics: Energy 3 (2021): 032011, 10.1088/2515-7655/abfb4a.

[88] R. E. Owen , E. Wisniewska , M. Braglia , et al., “Operando Ultrasonic Monitoring of the Internal Temperature of Lithium‐ion Batteries for the Detection and Prevention of Thermal Runaway,” Journal of The Electrochemical Society 171 (2024): 040525, 10.1149/1945-7111/ad3beb.

[89] W. Chang , G. Thorsteinsson , U. Janakiraman , et al., “Relating Chemo‐Mechanical Hysteresis and Formation Protocols for Anode‐Free Lithium Metal Batteries,” Journal of The Electrochemical Society 171 (2024): 040506, 10.1149/1945-7111/ad36e3.

[90] Y. Kim , J. Gjerde , H. Zheng , et al., “Operando Detection of Void Formation during Lithium Stripping in Solid‐State Batteries Using Single‐Frequency Impedance Analysis,” ACS Electrochemistry (2026), 10.1021/acselectrochem.5c00417.

[91] M. Dabiri Havigh , K. Marcoen , B. de la Fuente , et al., “Operando ORP‐EIS for Monitoring SEI Formation of Anode‐Free Li Metal Batteries,” ACS Applied Energy Materials 8 (2025): 12940–12951, 10.1021/acsaem.5c02142.

[92] Q. Wang , C. Zhao , S. Wang , et al., “Clarifying the Relationship between the Lithium Deposition Coverage and Microstructure in Lithium Metal Batteries,” Journal of the American Chemical Society 144 (2022): 21961–21971, 10.1021/jacs.2c08849. 36416753 PMC9732870

[93] J. S. Yoon , D. W. Liao , S. M. Greene , et al., “Thermodynamics, Adhesion, and Wetting at Li/Cu (‐Oxide) Interfaces: Relevance for Anode‐Free Lithium–Metal Batteries,” ACS Applied Materials & Interfaces 16 (2024): 18790–18799, 10.1021/acsami.3c19034. 38587488

[94] C. Heubner , S. Maletti , H. Auer , et al., “From Lithium‐Metal toward Anode‐Free Solid‐State Batteries: Current Developments, Issues, and Challenges,” Advanced Functional Materials 31 (2021): 2106608, 10.1002/adfm.202106608.

[95] H. He , Y. Liu , Q. Liu , et al., “Failure Investigation of LiFePO4 Cells in over‐Discharge Conditions,” Journal of The Electrochemical Society 160 (2013): A793, 10.1149/2.039306jes.

[96] L. Guo , D. B. Thornton , M. A. Koronfel , I. E. Stephens , and M. P. Ryan , “Degradation in Lithium Ion Battery Current Collectors,” Journal of Physics: Energy 3 (2021): 032015, 10.1088/25157655/ac0c04.

[97] P. Arora , R. E. White , and M. Doyle , “Capacity Fade Mechanisms and Side Reactions in Lithium‐Ion Batteries,” Journal of The Electrochemical Society 145 (3647), 10.1149/1.1838857.

[98] M. Zhao , H. D. Dewald , F. R. Lemke , and R. J. Staniewicz , “Electrochemical Stability of Graphite‐Coated Copper in Lithium‐Ion Battery Electrolytes,” Journal of The Electrochemical Society 147 (2000): 3983, 10.1149/1.1394007.

[99] P. G. Kitz , M. J. Lacey , P. Novák , and E. J. Berg , “Operando EQCM‐D with Simultaneous in Situ EIS: New Insights into Interphase Formation in Li Ion Batteries,” Analytical Chemistry 91 (2019): 2296–2303, 10.1021/acs.analchem.8b04924. 30569698

[100] F. Baakes , D. Witt , and U. Krewer , “Impact of Electrolyte Impurities and SEI Composition on Battery Safety,” Chemical Science 14 (2023): 13783–13798, 10.1039/D3SC04186G. 38075652 PMC10699578

[101] S. J. An , J. Li , Z. Du , C. Daniel , and D. L. Wood , “Fast Formation Cycling for Lithium Ion Batteries,” Journal of Power Sources 342 (2017): 846–852, 10.1016/j.jpowsour.2017.01.011.

[102] K. Darwaish , Y.‐S. Wu , S.‐H. Wu , et al., “In‐situ Formed Li_2_O and an Artificial Protective Layer on Copper Current Collectors to Enhance the Cycling Stability of Lithium Metal Anode Batteries,” Journal of Energy Storage 100 (2024): 113508, 10.1016/j.est.2024.113508.

[103] I. Platzman , R. Brener , H. Haick , and R. Tannenbaum , “Oxidation of Polycrystalline Copper Thin Films at Ambient Conditions,” The Journal of Physical Chemistry C 112 (2008): 1101–1108, 10.1021/jp076981k.

[104] C. Gattinoni and A. Michaelides , “Atomistic Details of Oxide Surfaces and Surface Oxidation: the Example of Copper and Its Oxides,” Surface Science Reports 70 (2015): 424–447, 10.1016/j.surfrep.2015.07.001.

[105] R. Guo and B. M. Gallant , “Li2O solid Electrolyte Interphase: Probing Transport Properties at the Chemical Potential of Lithium,” Chemistry of Materials 32 (2020): 5525–5533, 10.1021/acs.chemmater.0c00333.

[106] D. Strmcnik , I. E. Castelli , J. G. Connell , et al., “Electrocatalytic Transformation of HF Impurity to H_2_ and LiF in Lithium‐ion Batteries,” Nature Catalysis 1 (2018): 255–262, 10.1038/s41929-018-0047-z.

[107] H. L. Shuo Zhang , Z.‐Q. Liu , C. Yan b , and J.‐Q. Huang , “Re‐evaluating the Nano‐sized Inorganic Protective Layer on Cu Current Collector for Anode Free Lithium Metal Batteries,” Chinese Chemical Letters 35 (2024): 1–4, 10.1016/j.cclet.2023.109284.

[108] W. Shin and A. Manthiram , “A Facile Potential Hold Method for Fostering an Inorganic Solid‐electrolyte Interphase for Anode‐free Lithium‐metal Batteries,” Angewandte Chemie 134 (2022): 202115909, 10.1002/anie.202115909. 35043528

[109] M. Yeddala , L. Rynearson , and B. L. Lucht , “Modification of Carbonate Electrolytes for Lithium Metal Electrodes,” ACS Energy Letters 8 (2023): 4782–4793, 10.1021/acsenergylett.3c01709.

[110] S. A. Delp , O. Borodin , M. Olguin , C. G. Eisner , J. L. Allen , and T. R. Jow , “Importance of Reduction and Oxidation Stability of High Voltage Electrolytes and Additives,” Electrochimica Acta 209 (2016): 498–510, 10.1016/j.electacta.2016.05.100.

[111] Y. Wang and H. Noguchi , “Li Plating/Stripping Efficiency in Ether‐based Dilute Electrolyte for Anode‐free Lithium‐metal Batteries: Effect of Operating Potential Range on Subsequent SEI Layer Structure,” Batteries & Supercaps 6 (2023): 202300359, 10.1002/batt.202300359.

[112] Y. Cai , B. Qin , C. Li , et al., “Stable Lithium Metal Anode Achieved by Shortening Diffusion Path on Solid Electrolyte Interface Derived from Cu_2_O Lithiophilic Layer,” Chemical Engineering Journal 433 (2022): 133689, 10.1016/j.cej.2021.133689.

[113] N. Yamakawa , M. Jiang , and C. P. Grey , “Investigation of the Conversion Reaction Mechanisms for Binary Copper (II) Compounds by Solid‐state NMR Spectroscopy and X‐ray Diffraction,” Chemistry of Materials 21 (2009): 3162–3176, 10.1021/cm900581b.

[114] B. L. Rinkel , D. S. Hall , I. Temprano , and C. P. Grey , “Electrolyte Oxidation Pathways in Lithium‐ion Batteries,” Journal of the American Chemical Society 142 (2020): 15058–15074, 10.1021/jacs.0c06363. 32697590

[115] Q. Liu , A. Cresce , M. Schroeder , et al., “Insight on Lithium Metal Anode Interphasial Chemistry: Reduction Mechanism of Cyclic Ether Solvent and SEI Film Formation,” Energy Storage Materials 17 (2019): 366–373, 10.1016/j.ensm.2018.09.024.

[116] S. S. Zhang , X. Fan , and C. Wang , “An in‐situ Enabled Lithium Metal Battery by Plating Lithium on a Copper Current Collector,” Electrochemistry Communications 89 (2018): 23–26, 10.1016/j.elecom.2018.02.011.

[117] C.‐J. Huang , Y.‐C. Hsu , K. N. Shitaw , et al., “Lithium Oxalate as a Lifespan Extender for Anode‐free Lithium Metal Batteries,” ACS Applied Materials & Interfaces 14 (2022): 26724–26732, 10.1021/acsami.2c04693. 35639111

[118] V. Pande and V. Viswanathan , “Computational Screening of Current Collectors for Enabling Anode‐free Lithium Metal Batteries,” ACS Energy Letters 4 (2019): 2952–2959, 10.1021/acsenergylett.9b02306.

[119] J. Seo , J. Lim , H. Chang , et al., “Sustaining Surface Lithiophilicity of Ultrathin Li‐Alloy Coating Layers on Current Collector for Zero‐Excess Li‐Metal Batteries,” Small 20 (2024): 2402988, 10.1002/smll.202402988. 38982943

[120] L. Wichmann , S.‐K. Jiang , J. H. Thienenkamp , et al., “Origins of Lithium Inventory Reversibility with an Alloying Functional Layer in Anode‐free Lithium Metal Batteries,” Nature Communications 16 (7216), 10.1038/s41467-025-62289-6. PMC12325613 40764294

[121] S. Pyo , S. Ryu , Y. J. Gong , et al., “Lithiophilic Wetting Agent Inducing Interfacial Fluorination for Long‐Lifespan Anode‐Free Lithium Metal Batteries,” Advanced Energy Materials 13 (2023): 2203573, 10.1002/aenm.202203573.

[122] X. Xiong , R. Zhi , Q. Zhou , et al., “A Binary PMMA/PVDF Blend Film Modified Substrate Enables a Superior Lithium Metal Anode for Lithium Batteries,” Materials Advances 2 (2021): 4240–4245, 10.1039/D1MA00121C.

[123] Y. Liu , S. Zhou , Z. Ma , et al., “Lithiophilic Zn–Ag Alloy Film‐modified Cu Current Collector Prepared by Magnetron Sputtering for Stable Lithium Metal Batteries,” Journal of Materials Chemistry A 13 (2025): 36729–36740, 10.1039/D5TA05802C.

[124] S. Zhou , Y. Liu , Z. Ma , et al., “Anode‐free Lithium Metal Batteries with Enhanced Durability: Magnetron‐sputtered Lithiophilic Bimetallic Coatings on Cu Current Collectors,” Chemical Engineering Journal 522 (2025): 167522, 10.1016/j.cej.2025.167522.

[125] M. Liu , C. Wang , Z. Cheng , et al., “Controlling the Lithium‐metal Growth to Enable Low‐lithium‐metal‐excess all‐solid‐state Lithium‐metal Batteries,” ACS Materials Letters 2 (2020): 665–670, 10.1021/acsmaterialslett.0c00152.

[126] S. Liu , X. Zhang , R. Li , L. Gao , and J. Luo , “Dendrite‐free Li Metal Anode by Lowering Deposition Interface Energy with Cu99Zn Alloy Coating,” Energy Storage Materials 14 (2018): 143–148, 10.1016/j.ensm.2018.03.004.

[127] D. Zhang , A. Dai , M. Wu , et al., “Lithiophilic 3D Porous CuZn Current Collector for Stable Lithium Metal Batteries,” ACS Energy Letters 5 (2019): 180–186, 10.1021/acsenergylett.9b01987.

[128] Q. Li , H. Pan , W. Li , et al., “Homogeneous Interface Conductivity for Lithium Dendrite‐free Anode,” ACS Energy Letters 3 (2018): 2259–2266, 10.1021/acsenergylett.8b01244.

[129] L. Yu , N. L. Canfield , S. Chen , et al., “Enhanced Stability of Lithium Metal Anode by Using a 3D Porous Nickel Substrate,” ChemElectroChem 5 (2018): 761–769, 10.1002/celc.201701250.

[130] N. Li , T. Jia , Y. Liu , et al., “Super‐three‐dimensional Lithiophilic Cu‐based Current Collector for Anode‐free Lithium Metal Battery,” Materials Today Energy 36 (2023): 101341, 10.1016/j.mtener.2023.101341.

[131] Z. Zhang , Y. Jin , Y. Zhao , et al., “Homogenous Lithium Plating/Stripping Regulation by a Mass‐producible Zn Particles Modified Li‐metal Composite Anode,” Nano Research 14 (2021): 3999–4005, 10.1007/s122740213326y.

[132] C. Chen , Y. Yang , and H. Shao , “Enhancement of the Lithium Cycling Capability Using Li–Zn Alloy Substrate for Lithium Metal Batteries",” Electrochimica Acta 137 (2014): 476–483, 10.1016/j.electacta.2014.06.006.

[133] H. Ye , Z.‐J. Zheng , H.‐R. Yao , et al., “Guiding Uniform Li Plating/Stripping through Lithium–aluminum Alloying Medium for Long‐life Li Metal Batteries,” Angewandte Chemie International Edition 58 (2019): 1094–1099, 10.1002/anie.201811955. 30447094

[134] S. Cui , P. Zhai , W. Yang , et al., “Large‐scale Modification of Commercial Copper Foil with Lithiophilic Metal Layer for Li Metal Battery,” Small 16 (2020): 1905620, 10.1002/smll.201905620. 31943735

[135] K. Fu , Y. Gong , Z. Fu , et al., “Transient Behavior of the Metal Interface in Lithium Metal–garnet Batteries,” Angewandte Chemie International Edition 56 (2017): 14942–14947, 10.1002/anie.201708637. 28994191

[136] Y.‐G. Lee , S. Fujiki , C. Jung , et al., “High‐energy Long‐cycling All‐solid‐state Lithium Metal Batteries Enabled by Silver–carbon Composite Anodes,” Nature Energy 5 (2020): 299–308, 10.1038/s415600200575z.

[137] Y. Wang , Z. Qu , S. Geng , et al., “Anode‐Free Lithium Metal Batteries Based on an Ultrathin and Respirable Interphase Layer,” Angewandte Chemie 135 (2023): 202304978, 10.1002/anie.202304978. 37139890

[138] H. H. Weldeyohannes , L. H. Abrha , Y. Nikodimos , et al., “Guiding Lithium‐ion Flux to Avoid Cell's Short Circuit and Extend Cycle Life for an Anode‐free Lithium Metal Battery,” Journal of Power Sources 506 (2021): 230204, 10.1016/j.jpowsour.2021.230204.

[139] Z. Zhang , H. Luo , Z. Liu , et al., “A Chemical Lithiation Induced Li_4.4_Sn Lithiophilic Layer for Anode‐free Lithium Metal Batteries,” Journal of Materials Chemistry A 10 (2022): 9670–9679, 10.1039/D2TA00167E.

[140] Y.‐X. Song , W.‐Y. Lu , Y.‐J. Chen , et al., “Coating Highly Lithiophilic Zn on Cu Foil for High‐performance Lithium Metal Batteries,” Rare Metals (2022): 1–10, 10.1007/s12598021018113.34539132

[141] A. Shao , X. Tang , M. Zhang , M. Bai , and Y. Ma , “Challenges, Strategies, and Prospects of the Anode‐Free Lithium Metal Batteries,” Advanced Energy and Sustainability Research 3 (2022): 2100197, 10.1002/aesr.202100197.

[142] S. Liu , A. Wang , Q. Li , et al., “Crumpled Graphene Balls Stabilized Dendrite‐free Lithium Metal Anodes,” Joule 2 (2018): 184–193, 10.1016/j.joule.2017.11.004.

[143] C. Yang , Y. Yao , S. He , H. Xie , E. Hitz , and L. Hu , “Ultrafine Silver Nanoparticles for Seeded Lithium Deposition toward Stable Lithium Metal Anode,” Advanced materials 29 (2017): 1702714, 10.1002/adma.201702714. 28833607

[144] P. Xue , S. Liu , and X. Shi , “A Hierarchical Silver‐nanowire–graphene Host Enabling Ultrahigh Rates and Superior Long‐term Cycling of Lithium‐metal Composite Anodes,” Advanced Materials 30 (2018): 1804165, 10.1002/adma.201804165. 30247780

[145] B. Hong , H. Fan , X.‐B. Cheng , et al., “Spatially Uniform Deposition of Lithium Metal in 3D Janus Hosts,” Energy Storage Materials 16 (2019): 259–266, 10.1016/j.ensm.2018.04.032.

[146] R. Weber , M. Genovese , A. J. Louli , et al., “Long Cycle Life and Dendrite‐free Lithium Morphology in Anode‐free Lithium Pouch Cells Enabled by a Dual‐salt Liquid Electrolyte,” Nature Energy 4 (2019): 683–689, 10.1038/s4156001904289.

[147] A. Eldesoky , A. Louli , A. Benson , and J. Dahn , “Cycling Performance of NMC811 Anode‐free Pouch Cells with 65 Different Electrolyte Formulations,” Journal of The Electrochemical Society 168 (2021): 120508, 10.1149/19457111/ac39e3.

[148] P. Liang , H. Sun , C.‐L. Huang , et al., “A Nonflammable High‐Voltage 4.7 V Anode‐Free Lithium Battery,” Advanced Materials 34 (2022): 2207361, 10.1002/adma.202207361. 36193778

[149] T. Pathirana , R. Kerr , M. Forsyth , and P. C. Howlett , “Application of Super‐concentrated Phosphonium Based Ionic Liquid Electrolyte for Anode‐free Lithium Metal Batteries,” Sustainable Energy & Fuels 5 (2021): 4141–4152, 10.1039/D1SE00724F.

[150] Z. Lu , H. Yang , Q. H. Yang , P. He , and H. Zhou , “Building a Beyond Concentrated Electrolyte for High‐Voltage Anode‐Free Rechargeable Sodium Batteries,” Angewandte Chemie International Edition 61 (2022): 202200410, 10.1002/anie.202200410. 35226757

[151] H. Li , C. Yan , and S. Wang , “Solvation Chemistry in Liquid Electrolytes for Rechargeable Lithium Batteries at Low Temperatures,” EcoEnergy 3 (2025): 387–421, 10.1002/ece2.94.

[152] Y. Zhou , P. Wang , K. Wang , et al., “Developing High‐Performance Anode‐Free Lithium Batteries: Challenges, Strategies, and Opportunities,” Advanced Functional Materials 35 (2025): 2424022, 10.1002/adfm.202424022.

[153] T. Wang , R. Wan , Z. Tang , et al., “Dual‐Salts Localized High‐Concentration Electrolyte for Li‐ and Mn‐Rich High‐Voltage Cathodes in Lithium Metal Batteries,” Small 20 (2024): 2401364, 10.1002/smll.202401364. 38874055

[154] S. Chen , J. Zheng , D. Mei , et al., “High‐Voltage Lithium‐Metal Batteries Enabled by Localized High‐Concentration Electrolytes,” Advanced Materials 30 (2018): 1706102, 10.1002/adma.201706102. 29575163

[155] X. Chen and H. Yu , “A Computational Review on Localized High‐Concentration Electrolytes in Lithium Batteries,” ChemElectroChem 11 (2024): 202400444, 10.1002/celc.202400444.

[156] X. Ren , L. Zou , X. Cao , et al., “Enabling High‐Voltage Lithium‐Metal Batteries under Practical Conditions,” Joule 3 (2019): 1662–1676, 10.1016/j.joule.2019.05.006.

[157] P. Ma , R. Kumar , K.‐H. Wang , and C. V. Amanchukwu , “Active Learning Accelerates Electrolyte Solvent Screening for Anode‐free Lithium Metal Batteries,” Nature Communications 16 (8396), 10.1038/s41467-025-63303-7. PMC12462480 40998777

[158] M. H. F. Jordi Sastre , L. Pompizi , A. Aribia , et al., “Blocking Lithium Dendrite Growth in Solid‐state Batteries with an Ultrathin Amorphous Li‐La‐Zr‐O Solid Electrolyte,” Communications Materials 2 (2021): 1–10, 10.1038/s43246-021-00177-4.

[159] S. J. An , J. Li , C. Daniel , D. Mohanty , S. Nagpure , and D. L. Wood , “The state of Understanding of the Lithium‐ion‐battery Graphite Solid Electrolyte Interphase (SEI) and Its Relationship to Formation Cycling,” Carbon 105 (2016): 52–76, 10.1016/j.carbon.2016.04.008.

[160] F. Wang , B. Wang , J. Li , et al., “Prelithiation: a Crucial Strategy for Boosting the Practical Application of Next‐Generation Lithium Ion Battery,” ACS Nano 15 (2021): 2197–2218, 10.1021/acsnano.0c10664. 33570903

[161] W. Lee , Y. S. Byeon , S. Lee , S. Kong , M.‐S. Park , and W.‐S. Yoon , “Over‐ and Hyper‐Lithiated Oxides as Sacrificial Cathodes for Lithium‐Ion Batteries,” Advanced Energy Materials (2024), 10.1002/aenm.202304533.

[162] J. M. Tarascon and D. Guyomard , “Li Metal‐Free Rechargeable Batteries Based on Li_1 + x_Mn_2_O_4_ Cathodes ( 0 ≤ x ≤ 1 ) and Carbon Anodes",” Journal of The Electrochemical Society 138 (2019): 2864–2868, 10.1149/1.2085331.

[163] W. M. Dose , S. Kim , Q. Liu , et al., “Dual Functionality of Over‐lithiated NMC for High Energy Silicon‐based Lithium‐ion Batteries,” Journal of Materials Chemistry A 9 (2021): 12818–12829, 10.1039/d1ta01290h.

[164] Y. K. Yirui Zhang , R. Tatara , L. Giordano , et al., “Revealing Electrolyte Oxidation via Carbonate Dehydrogenation on Ni‐based Oxides in Li‐ion Batteries by in Situ Fourier Transform Infrared Spectroscopy,” Energy & Environmental Science 13 (2020): 183–199, 10.1039/c9ee02543j.

[165] S. K. Heiskanen , J. Kim , and B. L. Lucht , “Generation and Evolution of the Solid Electrolyte Interphase of Lithium‐Ion Batteries,” Joule 3 (2019): 2322–2333, 10.1016/j.joule.2019.08.018.

[166] J. P. V. Bernardine , L. D. Rinkel , N. Garcia‐Araez , and C. P. Grey , “Two Electrolyte Decomposition Pathways at Nickel‐rich Cathode Surfaces in Lithium‐ion Batteries,” Energy & Environmental Science 15 (2022): 3416, 10.1039/d1ee04053g. 36091097 PMC9368649

[167] M. A. W. Kassie Nigus Shitaw , Y. Nikodimos , T. M. Tekaligne , et al., “Fundamental Phenomena in Anode‐free Coin Cells and Pouch Cells Configured with Imide Salt‐based Ether Electrolytes,” Materials Today Energy 39 (2024): 101461, 10.1016/j.mtener.2023.101461.

[168] C. Zhou , A. J. Samson , M. A. Garakani , and V. Thangadurai , “Communication—Anode‐Free Lithium Metal Batteries: a Case Study of Compression Effects on Coin Cell Performance",” Journal of The Electrochemical Society (2021): 168, 10.1149/1945-7111/ac0998.

[169] C. Niu , H. Lee , S. Chen , et al., “High‐energy Lithium Metal Pouch Cells with Limited Anode Swelling and Long Stable Cycles,” Nature Energy 4 (2019): 551–559, 10.1038/s41560-019-0390-6.

[170] A. J. Louli , A. Eldesoky , R. Weber , et al., “Diagnosing and Correcting Anode‐free Cell Failure via Electrolyte and Morphological Analysis,” Nature Energy 5 (2020): 693–702, 10.1038/s41560-020-0668-8.

[171] Z. Yu , H. Wang , X. Kong , et al., “Molecular Design for Electrolyte Solvents Enabling Energy‐dense and Long‐cycling Lithium Metal Batteries,” Nature Energy 5 (2020): 526–533, 10.1038/s41560-020-0634-5.

[172] J. Qian , B. D. Adams , J. Zheng , et al., “Anode‐Free Rechargeable Lithium Metal Batteries,” Advanced Functional Materials 26 (2016): 7094–7102, 10.1002/adfm.201602353.

[173] J.‐J. Woo , V. A. Maroni , G. Liu , et al., “Symmetrical Impedance Study on Inactivation Induced Degradation of Lithium Electrodes for Batteries beyond Lithium‐Ion,” Journal of The Electrochemical Society 161 (2014): A827, 10.1149/2.089405jes.

[174] Y.‐H. Lin , L.‐T. Wu , Y.‐T. Zhan , et al., “Self‐assembly Formation of Solid‐electrolyte Interphase in Gel Polymer Electrolytes for High Performance Lithium Metal Batteries,” Energy Storage Materials 61 (2023): 102868, 10.1016/j.ensm.2023.102868.

[175] L. Ye , C. Zhang , Y. Zhou , B. Ülgüt , Y. Zhao , and J. Qian , “Guided Lithium Nucleation and Growth on Lithiophilic Tin‐decorated Copper Substrate,” Journal of Energy Chemistry 74 (2022): 412–419, 10.1016/j.jechem.2022.07.027.

[176] X.‐L. Zhang , L. Ma , Y.‐P. Cai , J. Fransaer , and Q. Zheng , “A Low‐Fermi‐level Current Collector Enables Anode‐free Lithium Metal Batteries with Long Cycle Life,” Matter 7 (2024): 583–602, 10.1016/j.matt.2023.11.017.

[177] N. Li , G. Kang , S. Huang , et al., “Protonated Polyaniline‐Modified Copper Current Collector for Anode‐Free Lithium Metal Battery,” ACS Applied Materials & Interfaces 17 (2025): 36627–36638, 10.1021/acsami.5c04173. 40493616

[178] N. A. Sahalie , Z. T. Wondimkun , W.‐N. Su , et al., “Multifunctional Properties of Al_2_O_3_/Polyacrylonitrile Composite Coating on Cu to Suppress Dendritic Growth in Anode‐Free Li‐Metal Battery,” ACS Applied Energy Materials 3 (2020): 7666–7679, 10.1021/acsaem.0c01080.

[179] R. Marrache , T. Mukra , P. Shekhter , and E. Peled , “Enhancing Performance of Anode‐free Li‐metal Batteries by Addition of Ceramic Nanoparticles Part II,” Journal of Solid State Electrochemistry 26 (2022): 2027–2038, 10.1007/s10008-022-05163-5.

[180] M. C. Baptista , B. M. Gomes , A. B. Vale , and M. H. Braga , “In‐series all‐solid‐state Anode‐less Cells,” Journal of Energy Storage 102 (2024): 113983, 10.1016/j.est.2024.113983.

[181] J. Oh , Y. Sohn , and J. W. Choi , “High‐performance Anode‐less all‐solid‐state Batteries Enabled by Multisite Nucleation and an Elastic Network,” EES Batteries 1 (2025): 566–575, 10.1039/D5EB00050E. 40255540 PMC12004216

[182] N. Lee , J. Oh , and J. W. Choi , “Anode‐less All‐solid‐State Batteries: Recent Advances and Future Outlook,” Materials Futures 2 (2023): 013502, 10.1088/2752-5724/acb3e8.

[183] M. Ma , M. Zhang , B. Jiang , Y. Du , B. Hu , and C. Sun , “A Review of all‐solid‐state Electrolytes for Lithium Batteries: High‐voltage Cathode Materials, Solid‐state Electrolytes and Electrode–electrolyte Interfaces, Solid‐state Electrolytes and Electrode–electrolyte Interfaces,” Materials Chemistry Frontiers 7 (2023): 1268–1297, 10.1039/D2QM01071B.

[184] A. Joshi , D. K. Mishra , R. Singh , J. Zhang , and Y. Ding , “A Comprehensive Review of Solid‐state Batteries,” Applied Energy 386 (2025): 125546, 10.1016/j.apenergy.2025.125546.

[185] J. Becker , T. Fuchs , T. Ortmann , et al., “Microstructure of Lithium Metal Electrodeposited at the Steel|Li6PS5Cl Interface in “Anode‐Free” Solid‐State Batteries,” Advanced Energy Materials 15 (2025): 2404975, 10.1002/aenm.202404975.

[186] Y. Duan , X. Bai , T. Yu , et al., “Research Progress and Prospect in Typical Sulfide Solid‐state Electrolytes,” Journal of Energy Storage 55 (2022): 105382, 10.1016/j.est.2022.105382.

[187] N. Zamperlin , R. Cid , V. Kekkonen , et al., “Advanced Manufacturing of Thin‐film Lithium Metal Anode by Pulsed‐laser Deposition for next‐generation Solid‐state Batteries,” Journal of Power Sources 655 (2025): 237986, 10.1016/j.jpowsour.2025.237986.

[188] A. Orue Mendizabal , M. Cheddadi , A. Tron , A. Beutl , and P. López‐Aranguren , “Understanding Interfaces at the Positive and Negative Electrodes on Sulfide‐Based Solid‐State Batteries,” ACS Applied Energy Materials 6 (2023): 11030–11042, 10.1021/acsaem.3c01894. 38020742 PMC10646897

[189] J.‐M. Doux , Y. Yang , D. H. S. Tan , et al., “Pressure Effects on Sulfide Electrolytes for all Solid‐state Batteries,” Journal of Materials Chemistry A 8 (2020): 5049–5055, 10.1039/C9TA12889A.

[190] A. Sharafi , H. M. Meyer , J. Nanda , J. Wolfenstine , and J. Sakamoto , “Characterizing the Li–Li_7_La_3_Zr_2_O_12_ Interface Stability and Kinetics as a Function of Temperature and Current Density",” Journal of Power Sources 302 (2016): 135–139, 10.1016/j.jpowsour.2015.10.053.

[191] C. Haslam and J. Sakamoto , “Stable Lithium Plating in “Lithium Metal‐free” Solid‐state Batteries Enabled by Seeded Lithium Nucleation,” Journal of the Electrochemical Society 170 (2023): 040524, 10.1149/19457111/accab4.

[192] A. O. Grazia Accardo , D. Chatzogiannakis , P. Gluchowski , M. Casas‐Cabanas , and P. Lopez‐Aranguren , “Fast and Low‐temperature Densification of Highly Conductive Li_7_La_3_Zr_2_O_12_ Ceramic Electrolytes for Solid‐state Batteries",” Journal of Power Sources 585 (2023): 233632, 10.1016/j.jpowsour.2023.233632.

[193] A. Sazvar , S. Ghahramani , O. Banapour Ghaffari , S. A. Zargar , and M. Golmohammad , “Review of Advances and Challenges in Li_7_La_3_Zr_2_O_12_ Solid Electrolytes: from Processing to Performance",” Journal of Power Sources 657 (2025): 238150, 10.1016/j.jpowsour.2025.238150.

[194] X. Tang , F. Xie , Y. Lu , et al., “Halide‐based Solid Electrolytes: Opportunities and Challenges in the Synergistic Development of all‐solid‐state Li/Na Batteries,” EES Batteries 1 (2025): 1481–1501, 10.1039/D5EB00064E.

[195] S. L. Pierre Lannelongue , E. Gonzalo , A. Golov , et al., “Stable Cycling of Halide Solid state Electrolyte Enabled by a Dynamic Layered Solid Electrolyte Interphase between Li Metal and Li_3_YCl_4_Br_2_",” Energy Storage Materials 72 (2024): 103733, 10.1016/j.ensm.2024.103733.

[196] C. Rosa , A. Pesce , P. Lannelongue , et al., “Understanding Interfacial Stability and Ionic Transport in Ethanol‐synthesized Li3InCl6 Solid Electrolyte for all‐solid‐state Batteries,” Journal of Physics and Chemistry of Solids 209 (2026): 113327, 10.1016/j.jpcs.2025.113327.

[197] J. Mu , S. Liao , L. Shi , et al., “Solid‐state Polymer Electrolytes in Lithium Batteries: Latest Progress and Perspective,” Polymer Chemistry 15 (2024): 473–499, 10.1039/D3PY01311A.

[198] S. C. Sand , J. L. M. Rupp , and B. Yildiz , “A Critical Review on Li‐ion Transport, Chemistry and Structure of Ceramic–polymer Composite Electrolytes for Solid state Batteries, Chemistry and Structure of Ceramic–polymer Composite Electrolytes for Solid state Batteries,” Chemical Society Reviews 54 (2025): 178–200, 10.1039/D4CS00214H. 39552376

[199] S. Risal , C. Wu , F. Wang , et al., “Silver‐carbon Interlayers in Anode‐free Solid‐state Lithium Metal Batteries: Current Development, Interfacial Issues, and Instability Challenges,” Carbon 213 (2023): 118225, 10.1016/j.carbon.2023.118225.

[200] J. Wang and H. Zhu , “Sulfide‐Based Anode‐Free Solid‐State Batteries: Key Challenges and Emerging Solutions,” ACS Energy Letters 10 (2025): 2377–2391, 10.1021/acsenergylett.5c00517. 40370945 PMC12070463

[201] F. Xie , M. S. Diallo , H. Kim , Q. H. Tu , and G. Ceder , “The Microscopic Mechanism of Lithiation and Delithiation in the Ag/C Buffer Layer for Anode‐Free Solid‐State Batteries,” Advanced Energy Materials 14 (2024): 2302960, 10.1002/aenm.202302960.

[202] J.‐S. Kim , G. Yoon , S. Kim , et al., “Surface Engineering of Inorganic Solid‐state Electrolytes via Interlayers Strategy for Developing Long‐cycling Quasi‐all‐solid‐state Lithium Batteries,” Nature Communications 14 (2023): 782, 10.1038/s41467023364017. PMC9922298 36774375

[203] J. Lee , S. H. Choi , G. Im , et al., “Room‐Temperature Anode‐Less all‐Solid‐State Batteries via the Conversion Reaction of Metal Fluorides,” Advanced Materials 34 (2022): 2203580, 10.1002/adma.202203580. 35953451

[204] L. Fallarino , U. N. Chishti , A. Pesce , et al., “Towards Lithium‐free Solid‐state Batteries with Nanoscale Ag/Cu Sputtered Bilayer Electrodes,” Chemical Communications 59 (2023): 12346–12349, 10.1039/D3CC02942E. 37767913

[205] A. Rafique , R. Cid , A. Pesce , et al., “Engineering Alloying and Conversion Interlayers for Anode‐Less Solid‐State Batteries,” ChemElectroChem 12 (2025): 202500346, 10.1002/celc.202500346.

[206] S. K. Dong‐Su Ko , S. Lee , G. Yoon , et al., “Mechanism of Stable Lithium Plating and Stripping in a Metal‐interlayer‐inserted Anode‐less Solid‐state Lithium Metal Battery,” Nature Communications 16 (2025): 1–13, 10.1038/s41467-025-55821-1. PMC11772618 39870642

[207] J. Oh , S. H. Choi , B. Chang , et al., “Elastic Binder for High‐performance Sulfide‐based All‐solid‐state Batteries,” ACS Energy Letters 7 (2022): 1374–1382, 10.1021/acsenergylett.2c00461.

[208] J. Oh , S. H. Choi , J. Y. Kim , et al., “Anode‐Less All‐Solid‐State Batteries Operating at Room Temperature and Low Pressure,” Advanced Energy Materials 13 (2023): 2301508, 10.1002/aenm.202301508.

[209] D. Jun , S. H. Park , J. E. Jung , et al., “Ultra‐Stable Breathing Anode for Li‐Free All‐Solid‐State Battery Based on Li Concentration Gradient in Magnesium Particles,” Advanced Functional Materials 34 (2024): 2310259, 10.1002/adfm.202310259.

[210] C. Wang , R. Cao , J. Zhou , and S. Jiao , “Three‐dimensional Lithium Metal Anodes in Solid‐state Batteries,” EES Batteries 2 (2026): 42–59, 10.1039/D5EB00156K.

[211] L. F. Amna Rafique , G. Accardo , A. Pesce , et al., “Interfacial Analysis of in‐situ Anode Formation in Solid‐state Batteries with Nanometric Current Collector,” Chemical Engineering Journal 509 (2025): 160956, 10.1016/j.cej.2025.160956.

[212] C. Yang , L. Zhang , B. Liu , et al., “Continuous Plating/Stripping Behavior of Solid‐state Lithium Metal Anode in a 3D Ion‐conductive Framework,” Proceedings of the National Academy of Sciences 115 (2018): 3770–3775, 10.1073/pnas.1719758115. PMC5899457 29581262

[213] Y. Zhang , W. Luo , C. Wang , et al., “High‐capacity, Low‐tortuosity, and Channel‐guided Lithium Metal Anode,” Proceedings of the National Academy of Sciences 114 (2017): 3584–3589, 10.1073/pnas.1618871114. PMC5389307 28320936

[214] S. A.‐O. Narayanan , U. Ulissi , J. S. Gibson , Y. A. Chart , R. S. Weatherup , and M. Pasta , “Effect of Current Density on the Solid Electrolyte Interphase Formation at the Lithium∣Li_6_PS_5_Cl Interface,” Nature Communications 13 (2022): 782, 10.1038/s41467022348559. PMC9700819 36433957

[215] C. Hänsel and D. Kundu , “The Stack Pressure Dilemma in Sulfide Electrolyte Based Li Metal Solid‐State Batteries: a Case Study with Li_6_PS_5_Cl Solid Electrolyte",” Advanced Materials Interfaces 8 (2021): 2100206, 10.1002/admi.202100206.

[216] C. Lee , J. Y. Kim , K. Y. Bae , et al., “Enhancing Electrochemomechanics: How Stack Pressure Regulation Affects all‐solid‐state Batteries,” Energy Storage Materials 66 (2024): 103196, 10.1016/j.ensm.2024.103196.

[217] J. Yu , X. Sun , X. Shen , et al., “Stack Pressure‐A Critical Strategy and Challenge in Performance Optimization of Solid state Batteries,” Energy Storage Materials 76 (2025): 104134, 10.1016/j.ensm.2025.104134.

[218] T. Fuchs , T. Ortmann , J. Becker , et al., “Analysis and Control of the Microstructure of Electrodeposited Alkali‐Metals in “Anode‐free” Solid‐State Batteries Using Electron‐Backscatter Diffraction with and without Interlayers,” Microscopy and Microanalysis 31 (2025): ozaf048.641, 10.1093/mam/ozaf048.641.

[219] J. B. Bates , N. J. Dudney , G. R. Gruzalski , et al., “Fabrication and Characterization of Amorphous Lithium Electrolyte Thin Films and Rechargeable Thin‐film Batteries,” Journal of Power Sources 43 (1993): 103–110, 10.1016/0378-7753(93)80106-Y.

[220] K. C. Yaoyu Ren , R. Chen , T. Liu , Y. Zhang , and C.‐W. Nan , “Oxide Electrolytes for Lithium Batteries,” Journal of the American Ceramic Society 98 (1998): 3603–3623, 10.1111/jace.13844.

[221] N. J. Dudney , “Addition of a Thin‐film Inorganic Solid Electrolyte (Lipon) as a Protective Film in Lithium Batteries with a Liquid Electrolyte,” Journal of Power Sources 89 (2000): 176–179, 10.1016/S0378-7753(00)00427-4.

[222] C. M. Juchuan Li , M. Chi , C. Liang , and N. J. Dudney , “Solid Electrolyte: the Key for High‐Voltage Lithium Batteries,” Advanced Energy Materials 5 (2015): 1401408, 10.1002/aenm.201401408.

[223] A. S. Westover , N. J. Dudney , R. L. Sacci , and S. Kalnaus , “Deposition and Confinement of Li Metal along an Artificial Lipon–Lipon Interface",” ACS Energy Letters 4 (2019): 651–655, 10.1021/acsenergylett.8b02542.

[224] T. A. W. Diyi Cheng , X. Wang , S. Wang , et al., “Unveiling the Stable Nature of the Solid Electrolyte Interphase between Lithium Metal and LiPON via Cryogenic Electron Microscopy,” Joule 4 (2020): 2484–2500, 10.1016/j.joule.2020.08.013.

[225] K. T. Diyi Cheng , S. Rao , Z. Wang , et al., “Manufacturing Scale‐Up of Anodeless Solid‐State Lithium Thin‐Film Batteries for High Volumetric Energy Density Applications,” ACS Energy Letters 8, no. 11 (2023): 4768–4774, 10.1021/acsenergylett.3c01839.

[226] B. Neudecker , N. Dudney , and J. Bates , ““Lithium‐Free” Thin‐Film Battery with in Situ Plated Li Anode,” Journal of the Electrochemical Society 147 (2000): 517, 10.1149/1.1393226.

[227] A. Müller , L. Paravicini , J. Morzy , et al., “Influence of Au, Pt, and C Seed Layers on Lithium Nucleation Dynamics for Anode‐Free Solid‐State Batteries,” ACS Applied Materials & Interfaces 16 (2023): 695–703, 10.1021/acsami.3c14693. 38124537 PMC10788862

[228] Z. Song , Z. Xing , J. Yang , et al., “Electrolyte Chemistry Development for Sodium‐Based Batteries: a Blueprint from Lithium or a Step towards Originality?,” Angewandte Chemie 64 (2025): 202424543, 10.1002/anie.202424543. PMC12124431 40169812

[229] A. Hemmelder , F. Tietze , S. Lux , J. Leker , L. Jahnke , and S. von Delft , “The Geostrategic Race for Leadership in Future Electric Vehicle Battery Technologies,” Energy & Environmental Science 18 (2025): 6117–6130, 10.1039/D5EE00301.

[230] Y.‐S. Hu and Y. Lu , “2019 Nobel Prize for the Li‐Ion Batteries and New Opportunities and Challenges in Na‐Ion Batteries,” ACS Energy Letters 4 (2019): 2689–2690, 10.1021/acsenergylett.9b02190.

[231] Y. Yung‐Fang Yu and J. T. Kummer , “Ion Exchange Properties of and Rates of Ionic Diffusion in Beta‐alumina,” Journal of Inorganic and Nuclear Chemistry 29 (1967): 2453–2475, 10.1016/0022-1902(67)80301-4.

[232] J. L. Sudworth , “Zebra Batteries,” Journal of Power Sources 51 (1994): 105–114, 10.1016/0378-7753(94)01967-3.

[233] J. B. Goodenough , H. Y. P. Hong , and J. A. Kafalas , “Fast Na^+^‐ion Transport in Skeleton Structures",” Materials Research Bulletin 11 (1976): 203–220, 10.1016/0025-5408(76)90077-5.

[234] Y. Nishi , Lithium‐Ion Batteries, ed. G. Pistoia , (Elsevier, 2014), 21–39.

[235] M. Schmidt , U. M. V. D. Bastian , and C. Kresse 2023. Rohstoffrisikobewertung – Lithium. DERA Rohstoffinformationen 54. Berlin: Deutsche Rohstoffagentur (DERA) in der Bundesanstalt für Geowissenschaften und Rohstoffe (BGR). ISBN 978‑3‑948532‑70‑3 (PDF), ISSN 2193‑5319.

[236] Systems, R.‐R.M.I. Lithium‐based batteries supply chain challenges , https://rmis.jrc.ec.europa.eu/analysis‐of‐supply‐chain‐challenges‐49b749.

[237] S. Bhattacharyya , S. Roy , X. Lin , et al., “Graphite: the New Critical Mineral,” Nature Reviews Materials 11 (2025): 65–78, 10.1038/s41578-025-00848-5.

[238] N. Yabuuchi , K. Kubota , M. Dahbi , and S. Komaba , “Research Development on Sodium‐Ion Batteries,” Chemical Reviews 114 (2014): 11636–11682, 10.1021/cr500192f. 25390643

[239] P. Voß , B. Gruber , M. Mitterfellner , et al., “Benchmarking state‐of‐the‐art Sodium‐ion Battery Cells—Modeling Energy Density and Carbon Footprint at the Gigafactory‐scale,” Energy & Environmental Science 18 (2025): 8104–8129, 10.1039/D5EE00415B.

[240] K. M. Abraham , “How Comparable Are Sodium‐Ion Batteries to Lithium‐Ion Counterparts?,” ACS Energy Letters 5 (2020): 3544–3547, 10.1021/acsenergylett.0c02181.

[241] W.‐Z. Huang , P. Xu , X.‐Y. Huang , et al., “Lithium Metal Anode: Past, Present, and Future,” MetalMat 1 (2024): 6, 10.1002/metm.6.

[242] M. Titirici , P. Johansson , M. C. Ribadeneyra , et al., “2024 roadmap for Sustainable Batteries,” Journal of Physics: Energy 6 (2024): 041502, 10.1088/2515-7655/ad6bc0.

[243] A. N. Dey , “Electrochemical Alloying of Lithium in Organic Electrolytes,” Journal of The Electrochemical Society 118 (1971): 1547, 10.1149/1.2407783.

[244] R. Shannon , “Revised Effective Ionic Radii and Systematic Studies of Interatomic Distances in Halides and Chalcogenides,” Acta Crystallographica Section A 32 (1976): 751–767, 10.1107/S0567739476001551.

[245] A. Robles‐Navarro , P. Jerabek , and P. Schwerdtfeger , “Tipping the Balance between the Bcc and Fcc Phase within the Alkali and Coinage Metal Groups,” Angewandte Chemie International Edition 63 (2024): 202313679, 10.1002/anie.202313679. 37877444

[246] H. Brooks , “Cohesive Energy of Alkali Metals,” Physical Review 91 (1953): 1027–1028, 10.1103/PhysRev.91.1027.

[247] N. Narayana and J. D. R. Burgess , Melting Points and Boiling Points for the Alkali Metals (National Institute of Standards and Technology, 2024), 10.6028/NIST.TN.2273.

[248] A. Masias , N. Felten , R. Garcia‐Mendez , J. Wolfenstine , and J. Sakamoto , “Elastic, Plastic, and Creep Mechanical Properties of Lithium Metal,” Journal of Materials Science 54 (2019): 2585–2600, 10.1007/s10853-018-2971-3.

[249] M. J. Wang , J.‐Y. Chang , J. B. Wolfenstine , and J. Sakamoto , “Analysis of Elastic, Plastic, and Creep Properties of Sodium Metal and Implications for Solid‐state Batteries,” Materialia 12 (2020): 100792, 10.1016/j.mtla.2020.100792.

[250] J. Jorné and C. W. Tobias , “Electrodeposition of the Alkali Metals from Propylene Carbonate,” Journal of Applied Electrochemistry 5 (1974): 279–290, 10.1007/BF00608791.

[251] F. Huang , P. Xu , G. Fang , and S. Liang , “In‐Depth Understanding of Interfacial Na^+^ Behaviors in Sodium Metal Anode: Migration, Desolvation, and Deposition,” Advanced Materials 36 (2024): 2405310, 10.1002/adma.202405310. 39152941

[252] M. Mandl , J. Becherer , D. Kramer , et al., “Sodium Metal Anodes: Deposition and Dissolution Behaviour and SEI Formation,” Electrochimica Acta 354 (2020): 136698, 10.1016/j.electacta.2020.136698.

[253] B. Sayahpour , W. Li , S. Bai , et al., “Quantitative Analysis of Sodium Metal Deposition and Interphase in Na Metal Batteries,” Energy & Environmental Science 17 (2024): 1216–1228, 10.1039/D3EE03141A.

[254] Y. Deng , J. Zheng , A. Warren , et al., “On the Reversibility and Fragility of Sodium Metal Electrodes,” Advanced Energy Materials 9 (2019): 1901651, 10.1002/aenm.201901651.

[255] Z. Hu , L. Liu , X. Wang , et al., “Current Progress of Anode‐Free Rechargeable Sodium Metal Batteries: Origin, Challenges, Strategies, and Perspectives,” Advanced Functional Materials (2024): 2313823, 10.1002/adfm.202313823.

[256] Y. Dong , C. Xu , H. Zhao , et al., “Interface Engineering Strategies for Realizing Anode‐Free Sodium Batteries: a Review,” Advanced Energy Materials (2025): 2500407, 10.1002/aenm.202500407.

[257] H. Li , F. Wu , J. Wang , et al., “Anode‐free Sodium Metal Batteries: Optimisation of Electrolytes and Interphases,” Energy & Environmental Science 18 (2025): 3887–3916, 10.1039/D5EE00136F.

[258] Z. Wang , J. Song , X. Li , et al., “Advanced Current Collector Design for High Energy Density Anode‐Free Sodium‐Ion Batteries,” Electron 3, no. 4 (2025): 70013, 10.1002/elt2.70013.

[259] C. Xie , K. Liang , H. Wu , et al., “Revealing the Formation Mechanism of Inactive Sodium in Anode‐Free Sodium Batteries: Crystal Mismatch and Weak Lattice Force,” Advanced Energy Materials 15 (2025): 2500351, 10.1002/aenm.202500351.

[260] J. Liu , L. Hou , A. Shao , et al., “A Chemically Pre‐sodiated Na_15_Sn_4_ Interphase Enables High‐reversibility Anode‐less Sodium‐metal Batteries,” Chemical Communications 61 (2025): 13888–13891, 10.1039/D5CC02976G. 40813563

[261] Z. Hu , L. Liu , X. Wang , et al., “In Situ Integration of Rapid Ion‐Diffusion Interlayers on Cu Current Collectors toward Ultrafast Anode‐Free Sodium Metal Batteries,” ACS Nano 19 (2025): 23193–23208, 10.1021/acsnano.5c05043. 40519174

[262] P. Xu , Z. Liu , S. Guo , et al., “In Situ Construction of NaF‐rich Solid Electrolyte Interphase with Metallic Ce Sites for Stable Anode‐Free Sodium Metal Batteries,” Angewandte Chemie International Edition 64, no. 49 (2025): 202515566, 10.1002/anie.202515566. 41041934

[263] J. Ruan , J. Hu , Q. Li , et al., “Current Collector Interphase Design for High‐energy and Stable Anode‐less Sodium Batteries,” Nature Sustainability 8 (2025): 530–541, 10.1038/s41893-025-01545-5.

[264] J. Shi , D. Wang , Q. Liu , Z. Yu , J.‐Q. Huang , and B. Zhang , “Intermetallic Layers with Tuned Na Nucleation and Transport for Anode‐Free Sodium Metal Batteries,” Nano Letters 25 (2025): 1800–1807, 10.1021/acs.nanolett.4c04282. 39870493 PMC11803737

[265] M. Li , X. Gong , Y. Hu , et al., “A Eutectic Aluminum–Tin Alloy Substrate for Anode‐Free Na Battery",” Small 21 (2025): 2411901, 10.1002/smll.202411901. 40166855

[266] Z. Wang , R. Tian , H. Jiang , et al., “A k Descriptor to Design of Current Collectors for Anode‐Free Sodium Batteries,” Advanced Materials 37 (2025): 2504760, 10.1002/adma.202504760. 40696969

[267] Y. An , Z. Pei , D. Luan , and X. W. Lou , “Foldable Anode‐free Sodium Batteries Enabled by N,P‐codoped Carbon Macroporous Fibers Incorporated with CoP Nanoparticles,” Science Advances 11 (2025): adv, 10.1126/sciadv.adv2007. PMC12063667 40344062

[268] H. Zhu , L. Peng , J. Wu , et al., “Fluorine‐doped Micropore‐covered Mesoporous Carbon Nanofibers for Long‐lasting Anode‐free Sodium Metal Batteries,” Nature Communications 16 (2025): 5494, 10.1038/s41467-025-60168-8. PMC12216415 40593488

[269] L. Peng , X. Huang , Q. Zeng , et al., “Fabrication of a Rigid‐Flexible and Dual‐Conductive Interphase on an Aluminum Current Collector for Ultra‐Stable Anode‐Free Sodium Batteries,” Small (2025): 08205, 10.1002/smll.202508205. 41026740

[270] L. Schafzahl , I. Hanzu , M. Wilkening , and S. A. Freunberger , “An Electrolyte for Reversible Cycling of Sodium Metal and Intercalation Compounds,” Chemsuschem 10 (2017): 401–408, 10.1002/cssc.201601222. 27860417

[271] Z. Xu , C. Lin , J. Qiu , and Z. Wang , “Polymer‐Regulated Solvation and Interphase Engineering for Long‐Life and Safe Quasi‐Solid‐State Anode‐Free Sodium Batteries,” Advanced Materials 37 (2025): 2506037, 10.1002/adma.202506037. 40504728

[272] Y. Zhao , Z. Ni , Z. Wang , et al., “From Liquid to Solid: Advanced Electrolyte Design Strategies for next‐generation High‐performance Anode‐free Lithium/Sodium/Potassium Batteries,” Energy Storage Materials 82 (2025): 104608, 10.1016/j.ensm.2025.104608.

[273] D. M. C. Ould , S. Menkin , H. E. Smith , et al., “Sodium Borates: Expanding the Electrolyte Selection for Sodium‐Ion Batteries,” Angewandte Chemie International Edition 61 (2022): 202202133, 10.1002/anie.202202133. PMC9401571 35415950

[274] E. S. Flitz , N. R. Singstock , C. Tezak , et al., “A Low‐cost, Fluorine‐free Electrolyte for Improved Sodium Batteries,” Joule 9 (2025): 102045, 10.1016/j.joule.2025.102045.

[275] Q. Ma and F. Tietz , “Solid‐State Electrolyte Materials for Sodium Batteries: towards Practical Applications,” ChemElectroChem 7 (2020): 2693–2713, 10.1002/celc.202000164.

[276] Q. Zhao , S. Stalin , C.‐Z. Zhao , and L. A. Archer , “Designing Solid‐state Electrolytes for Safe, Energy‐dense Batteries,” Nature Reviews Materials 5 (2020): 229–252, 10.1038/s41578-019-0165-5.

[277] Y. Li , X. Tang , Q. Li , et al., “Halide Solid‐State Electrolytes for all‐Solid‐State Sodium Batteries: Progress and Perspectives,” ACS Energy Letters 10 (2025): 5520–5541, 10.1021/acsenergylett.5c02748.

[278] K. Shi , B. Guan , Z. Zhuang , et al., “Recent Progress and Prospects on Sodium‐Ion Battery and all‐Solid‐State Sodium Battery: a Promising Choice of Future Batteries for Energy Storage,” Energy & Fuels 38 (2024): 9280–9319, 10.1021/acs.energyfuels.4c00980.

[279] Y.‐S. Hu and F. Xie , “Making Na‐Ion Batteries Solid,” ACS Energy Letters 9 (2024): 6081–6083, 10.1021/acsenergylett.4c03230.

[280] A. M. Skundin and T. L. Kulova , “All‐Solid‐State Anode‐Free Sodium Batteries: Challenges and Prospects,” Batteries 11 (2025): 292, 10.3390/batteries11080292.

[281] T. Ortmann , T. Fuchs , J. K. Eckhardt , Z. Ding , and Q. Ma , “Deposition of Sodium Metal at the Copper‐NaSICON Interface for Reservoir‐Free Solid‐State Sodium Batteries,” Advanced Energy Materials 14 (2024): 2302729, 10.1002/aenm.202302729.

[282] G. Deysher , J. A. Sam Oh , Y.‐T. Chen , et al., “Design Principles for Enabling an Anode‐free Sodium all‐solid‐state Battery,” Nature Energy 9 (2024): 1161–1172, 10.1038/s41560-024-01569-9.

[283] X. Liao , D. Liu , and J. Liu , “Anode‐Free Design with Pelletized Aluminium Current Collector Enables High‐Energy‐Density Sodium All‐Solid‐State Batteries,” Energy & Environmental Materials 8 (2025): 12883, 10.1002/eem2.12883.

[284] J. Huang , K. Wu , G. Xu , M. Wu , S. Dou , and C. Wu , “Recent Progress and Strategic Perspectives of Inorganic Solid Electrolytes: Fundamentals, Modifications, and Applications in Sodium Metal Batteries,” Chemical Society Reviews 52 (2023): 4933–4995, 10.1039/D2CS01029A. 37365900

[285] M.‐C. Bay , M. Wang , R. Grissa , M. V. F. Heinz , J. Sakamoto , and C. Battaglia , “Sodium Plating from Na‐β″‐Alumina Ceramics at Room Temperature, Paving the Way for Fast‐Charging all‐Solid‐State Batteries,” Advanced Energy Materials 10 (2020): 1902899, 10.1002/aenm.201902899.

[286] D. Spencer Jolly , Z. Ning , J. E. Darnbrough , et al., “Sodium/Na β″ Alumina Interface: Effect of Pressure on Voids,” ACS Applied Materials & Interfaces 12 (2020): 678–685, 10.1021/acsami.9b17786. 31815414

[287] S. E. Sandoval , C. G. Haslam , B. S. Vishnugopi , et al., “Electro‐chemo‐mechanics of Anode‐free Solid‐state Batteries,” Nature Materials 24 (2025): 673–681, 10.1038/s41563-024-02055-z. 39748055

[288] N.‐Y. Park , H.‐U. Lee , T.‐Y. Yu , et al., “High‐energy, Long‐life Ni‐rich Cathode Materials with Columnar Structures for all‐solid‐state Batteries,” Nature Energy 10 (2025): 479–489, 10.1038/s41560-025-01726-8.

[289] QuantumScape Corporation. 2024. Letter to Shareholders: Q3 Fiscal 2024. October 23, 2024. SAN JOSE, CA: QuantumScape Corporation. PDF available at https://s29.q4cdn.com/884415011/files/doc_financials/2024/q3/QS‐Shareholder‐Letter‐Q3‐2024.pdf.

[290] O. Garcia‐Calvo , A. Gutiérrez‐Pardo , I. Combarro , et al., “Selection and Surface Modifications of Current Collectors for Anode‐free Polymer‐based Solid‐state Batteries,” Frontiers in Chemistry 10 (2022): 934365, 10.3389/fchem.2022.934365. 35873050 PMC9300918

[291] A. J. Louli , A. Eldesoky , J. deGooyer , et al., “Different Positive Electrodes for Anode‐free Lithium Metal Cells,” Journal of The Electrochemical Society 169 (2022): 040517, 10.1149/19457111/ac62c4.

[292] A. J. Louli , M. Coon , M. Genovese , J. deGooyer , A. Eldesoky , and J. R. Dahn , “Optimizing Cycling Conditions for Anode‐free Lithium Metal Cells,” Journal of the Electrochemical Society 168 (2021): 020515, 10.1149/19457111/abe089.

[293] L. Liu and J. Wang , “Overcoming Copper Substrate Thermodynamic Limitations in Anode‐Free Lithium Pouch Cells via in Situ Seed Implantation,” Nano Letters 23 (2023): 10251–10258, 10.1021/acs.nanolett.3c02777. 37781986

[294] Q. Zhu , D. Yu , J. Chen , et al., “A 110 Wh Kg^−1^ Ah‐level Anode‐free Sodium Battery at −40° C",” Joule 8 (2024): 482–495, 10.1016/j.joule.2024.01.010.

[295] M. O. Ashley Willow , S. Kiani , N. Reynolds , et al., “Design and Validation of Anode‐Free Sodium‐Ion Pouch Cells Employing Prussian White Cathodes,” Batteries 11 (2025): 1–14, 10.3390/batteries11030097.

[296] G. Bree , D. Proprentner , V. Majherova , et al., “LiMnxFe_1−X_PO_4_ Anode Free Batteries: a Scalable, Low Cost, Energy Dense Lithium Cell Design,” Batteries & Supercaps (2025): 202500507, 10.1002/batt.202500507.

[297] Q. L. t. s. holders, https://www.quantumscape.com/technology.

